# AGR2 suppresses ferroptosis via the p53/FPN1 regulatory axis and drives therapeutic vulnerabilities in pancreatic cancer

**DOI:** 10.1038/s41419-025-08263-y

**Published:** 2025-12-01

**Authors:** Ziying Jian, Jialin Song, Liangqi Lu, Tao Pan, Hanzhe Zhang, Qi Zhang, Weiyu Zhang, Renjie Li, Jizhou Gong, Naipeng Shi, Shi Zuo, Tao Cheng, Yuhong Yang, Zhiheng Zhang

**Affiliations:** 1 Department of Hematology and Oncology, Zhongda Hospital, Medical School, Southeast University, Nanjing, China; 2 Medical School, Southeast University, Nanjing, China; 3 Center of Interventional Radiology and Vascular Surgery, Department of Radiology, Zhongda Hospital, Medical School, Southeast University, Nanjing, China; 4 Department of General Surgery, Zhongda Hospital, Medical School, Southeast University, Nanjing, China; 5 Department of Urology, Northern Jiangsu People’s Hospital, Yangzhou, China; 6 Department of Hepatobiliary Surgery, The Affiliated Hospital of Guizhou Medical University, Guiyang, China; 7 Department of Endocrinology, The First Affiliated Hospital with Nanjing Medical University, Nanjing, China; 8 Division of Hepatobiliary and Transplantation Surgery, Department of General Surgery, Nanjing Drum Tower Hospital, Affiliated Hospital of Medical School, Nanjing University, Nanjing, China

**Keywords:** Pancreatic cancer, Tumour immunology

## Abstract

Ferroptosis has emerged as a potential therapeutic target in cancer. This study shows the critical role of AGR2 in ferroptosis suppression across pancreatic cancer cell lines and in vivo models. Notably, human pancreatic cancer cells exhibit dose-dependent AGR2 upregulation upon exposure to ferroptosis inducers. Genetic ablation of AGR2 significantly sensitizes cells to ferroptosis through a p53-mediated mechanism, while p53 knockdown effectively rescues ferroptosis resistance. Mechanistic investigations demonstrate that AGR2 deficiency activates p53 signaling, downregulating the iron exporter SLC40A1 (encoding ferroportin/FPN1), inducing intracellular iron overload and consequent ferroptosis. Clinically, we find a positive correlation between AGR2 and FPN1 expression in PDAC specimens, with co-elevation of both markers predicting unfavorable patient prognosis. Therapeutically, administration of an AGR2-targeting peptide synergizes with ferroptosis inducers, significantly enhancing cell death in PDAC models. Our findings not only elucidate a novel AGR2/p53/FPN1 regulatory axis in ferroptosis control but also propose innovative combination strategies for pancreatic cancer treatment.

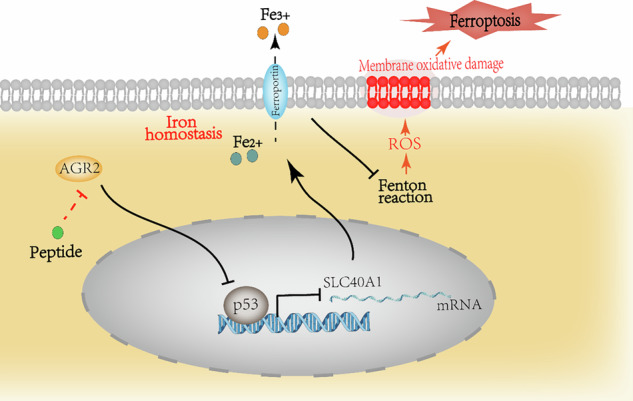

## Highlights

- AGR2 is upregulated during ferroptosis in pancreatic cancer.
- AGR2 is a negative regulator of ferroptosis in pancreatic cancer.
- AGR2 inhibits ferroportin expression by modulating p53.
- Ferroportin inhibited ferroptosis in pancreatic cancer.
- Peptide treatment sensitized PDAC cells to ferroptosis.

## Introduction

Pancreatic ductal adenocarcinoma (PDAC) ranks among the most lethal malignancies, with a five-year survival rate below 10%, underscoring an urgent need to unravel the molecular drivers of its aggressiveness and therapeutic resistance [1]. While dysregulation of oncogenic and tumor-suppressive pathways, such as KRAS activation and TP53 inactivation, has been extensively studied, emerging players like Anterior Gradient 2 (AGR2) are gaining attention for their roles in shaping PDAC biology [2–4]. AGR2, a protein disulfide isomerase overexpressed in pancreatic cancer, is implicated in metastasis, chemoresistance, and endoplasmic reticulum (ER) stress adaptation [2, 3, 5–8]. However, the mechanisms by which AGR2 intersects with canonical tumor-suppressor networks, particularly p53, and influences cell death pathways remain poorly defined.

Recent advances have positioned ferroptosis as a therapeutic vulnerability in apoptosis-resistant cancers like PDAC [9, 10]. Central to ferroptosis regulation is cellular iron homeostasis, governed by iron transporters such as ferroportin (FPN1), the sole iron exporter [11–13]. Intriguingly, the tumor suppressor p53, beyond its canonical roles in cell cycle arrest and apoptosis, has been shown to modulate ferroptosis through transcriptional control of iron metabolism genes [14, 15]. Yet, how oncogenic factors like AGR2 interface with p53 to disrupt iron flux and ferroptosis sensitivity in PDAC is unknown, representing a critical gap in understanding the disease’s adaptive mechanisms.

This study identifies AGR2 as a novel regulator of the p53-ferroportin axis in pancreatic cancer. We demonstrate that AGR2 suppresses p53 activity, leading to the transcriptional downregulation of FPN and subsequent iron retention, which shields PDAC cells from ferroptosis. This AGR2-driven axis not only promotes tumor survival under stress but also correlates with poor patient outcomes and resistance to standard-of-care therapies. Our work reveals a previously unrecognized crosstalk between AGR2, p53, and iron metabolism, positioning AGR2 as a rheostat of ferroptosis susceptibility.

## Results

### AGR2 is upregulated during ferroptosis in pancreatic cancer

**Fig. 1:**
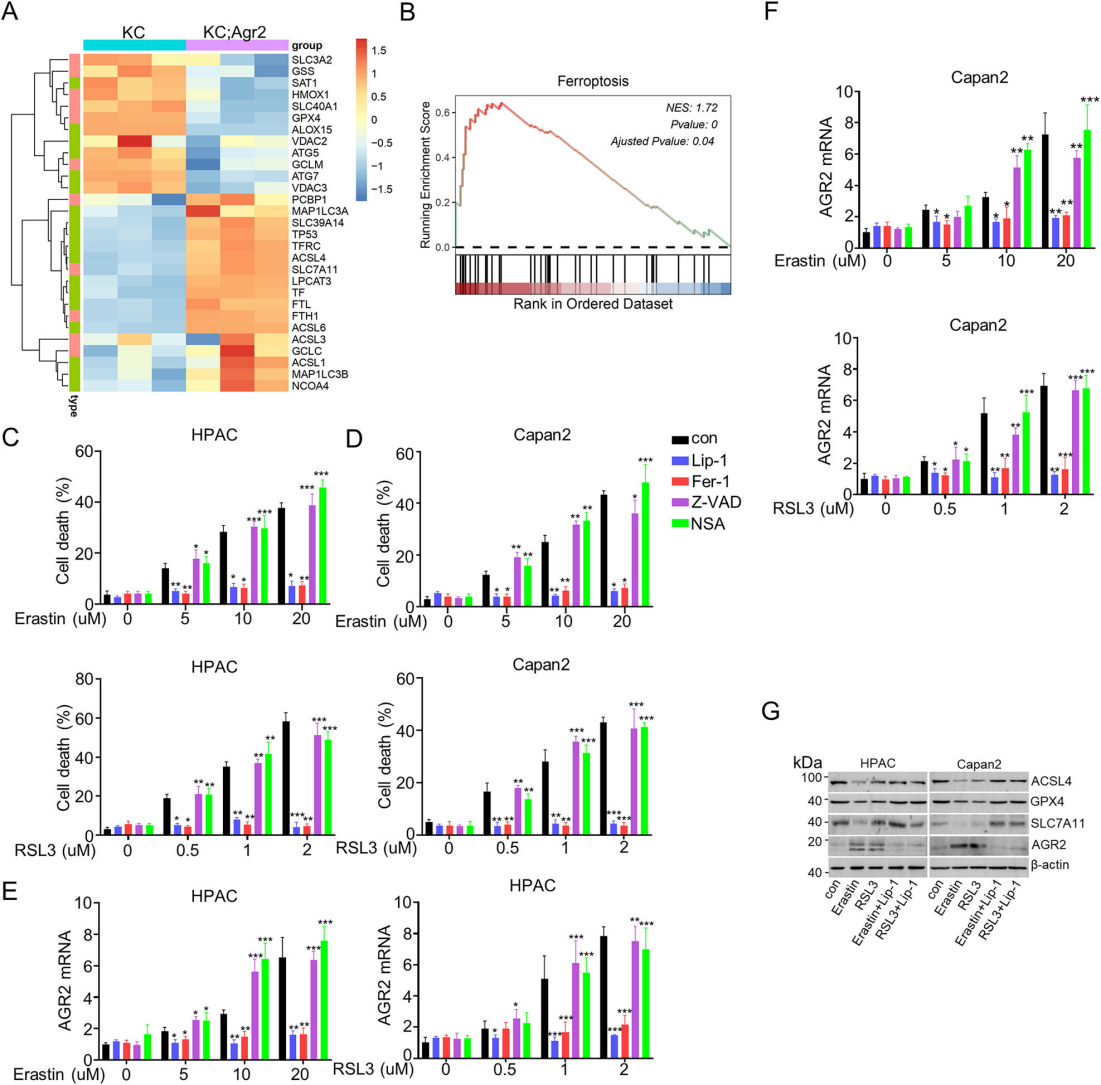
AGR2 is upregulated in pancreatic cancer during ferroptosis. **A** Heatmap showing the expression levels of ferroptosis-related genes in pancreatic tissues from KC and KC; Agr2^−/−^ mice. **B** Gene set enrichment analysis indicating significant enrichment of ferroptosis-related genes in pancreatic cancer. **C**–**F** Quantitative analysis showed the cell death rate and the AGR2 mRNA expression of Capan2/ HPAC cells treated with different concentrations of Erastin (0–20 µM) or RSL3 (0–2 µM). **G** Protein expression levels of ACSL4, GPX4, SLC7A11, and AGR2 in HPAC and Capan2 cells treated with Erastin, RSL3, Lip-1. Data are presented as the mean of 3 independent experiments ± SEM. *P < 0.1, **P < 0.01, ***P < 0.001.

Our previous studies have proved the role of AGR2 in pancreatic carcinogenesis and progression [2]. Further analysis of sequencing data of the pancreas of KC verse KC;Agr2^−/−^ mice (PRJEB40643) proved that ferroptosis might be involved in the pancreatic cancer progression (Fig. 1A). GSEA analysis presented that AGR2 knockout induced the enrichment of ferroptosis (Fig. 1B). Thus, we hypothesize that AGR2 might regulate pancreatic cancer progression by regulating ferroptosis. Firstly, we analyzed the relationship between cell death and mRNA expression of AGR2 in PDAC cell lines (HPAC and Capan2). Indeed, treatment with erastin or RSL3 induced cell death in a dose-dependent manner, and this effect was significantly attenuated by the ferroptosis-specific inhibitors liproxstatin-1 and ferrostatin-1 (Fig. 1C, D, Supplementary fig. 1A, B). Pharmacological inhibition of necroptosis (via necrosulfonamide [NSA]) and apoptosis (via Z-VAD-FMK) did not attenuate RSL3- or erastin-induced cell death in HPAC and Capan2 cell lines (Fig. 1C, D, Supplementary fig. 1A, B). Transcriptional profiling revealed erastin- and RSL3-mediated upregulation of AGR2 mRNA expression in these cells, a phenomenon suppressed by the ferroptosis inhibitors liproxstatin-1 and ferrostatin-1 but unaffected by Z-VAD-FMK or NSA (Fig. 1E, F, Supplementary fig. 1C, D). Subsequent western blot analysis corroborated these findings, demonstrating that liproxstatin-1 abolished erastin/RSL3-induced elevation of AGR2 protein levels (Fig. 1G, Supplementary fig. 1E). Consistent with established ferroptosis markers, treatment with erastin or RSL3 inhibited protein expression of the pro-ferroptotic enzyme acyl-CoA synthetase long-chain family member 4 (ACSL4), glutathione peroxidase 4 (GPX4), and solute carrier family 7 member 11 (SLC7A11) expression (Fig. 1G, Supplementary fig. 1E). Furthermore, we check the expression of AGR2 on the tumor of KPC tissues, the immunoblot and immunohistochemistry showed the high expression of AGR2 in the tumor (Supplementary fig. 1F, G). These collective results implicate AGR2 is highly upregulated during ferroptosis.

### AGR2 is a negative regulator of ferroptosis in pancreatic cancer

**Fig. 2:**
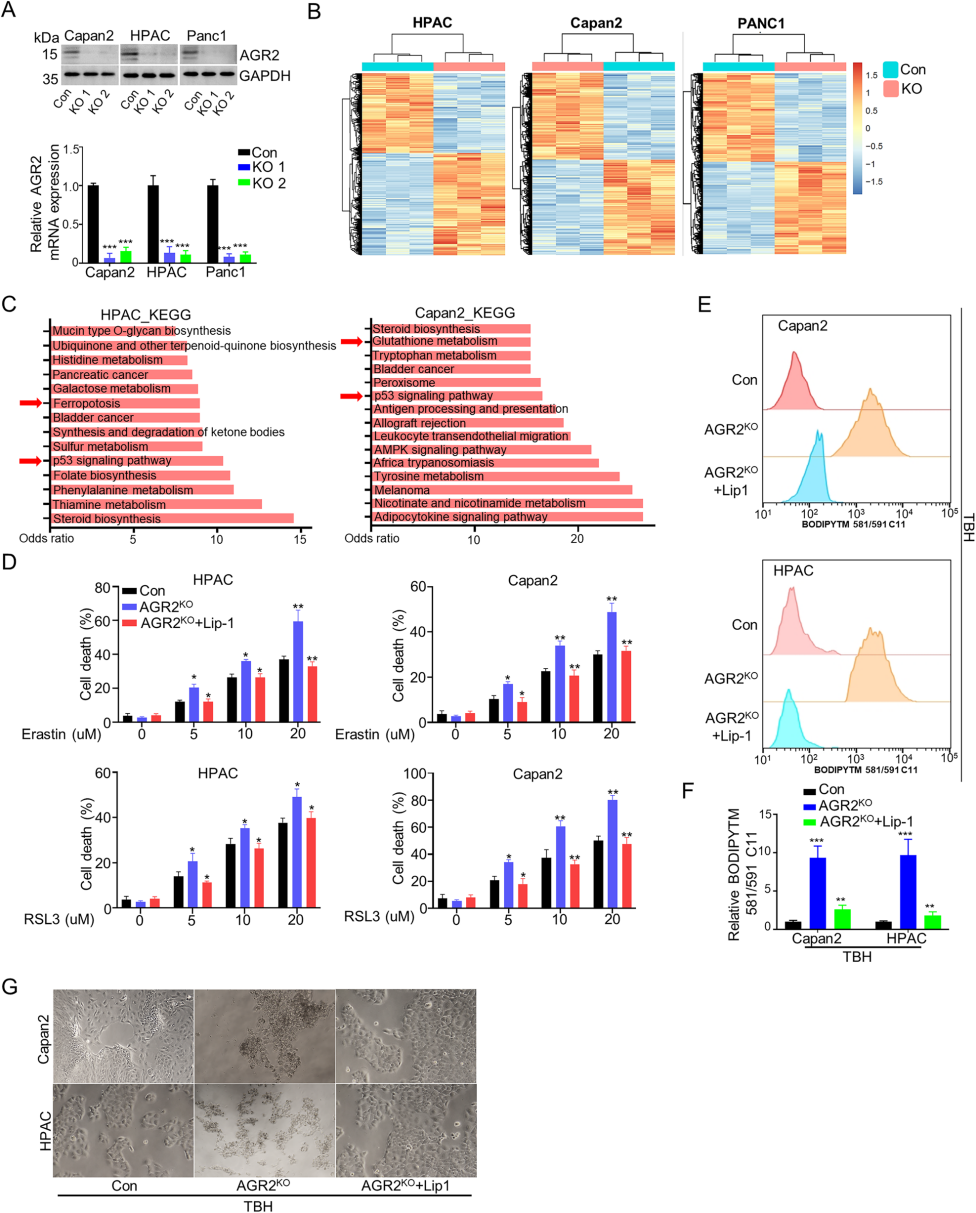
AGR2 sensitizes PDAC to ferroptosis in vitro. **A** Western blot analysis demonstrated expression of AGR2 protein in AGR2 knockout groups compared to control cells in Capan2, HPAC, and PANC1 cells; Quantification of AGR2 mRNA expression relative to GAPDH is shown beneath the blot. **B** Heatmap representation of dysregulated genes expression in AGR2 knockout groups compared to control cells of HPAC, Capan2, and PANC1 cells. **C** KEGG pathway enrichment analysis of differentially expressed genes in HPAC and Capan2 cells upon AGR2 knockout. **D** Quantitative analysis showed the cell death rate of Capan2 and HPAC cells with or without AGR2 knockout, treated with different concentrations of Erastin (0–20 µM) or RSL3 (0–20 µM). **E** BODIPYTM 581/591 C11 levels of Capan2 and HPAC cells with or without AGR2 knockout, treated with or without the Lip-1. **F** Quantification of BODIPY 581/591 C11 fluorescence in Capan2 and HPAC cells. **G** Representative bright-field images of HPAC and Capan2 cells treated with TBH to induce ferroptosis in control, AGR2 KO, and AGR2 KO + Lip1 groups. Scale bar, 250 µm. Data are presented as the mean of 3 independent experiments ± SEM. *P < 0.1, **P < 0.01, ***P < 0.001.

As AGR2 was identified as an oncogene in cancers, we hypothesized that AGR2 might be a negative regulator of ferroptosis. AGR2 knockout (KO) cell lines were established (Fig. 2A, Supplementary fig. 2A). RNA sequencing and KEGG analysis show ferroptosis might be involved in the AGR2 KO cellular process (Fig. 2B, C). And the ferroptosis might associated with the p53 pathways. Indeed, AGR2 KO sensitized RSL3 or erastin-induced cell death in PDAC cells with wold-type p53 (p53wt) (HPAC and Capan2) (Fig. 2D); While for the PDAC cells with mutated p53 (p53mut) (PANC1, Miacapa2, and AsPC-1), While AGR2 KO alone did not induce increased cell death. And AGR2 KO did not sensitized RSL3 or erastin-induced cell death (Supplementary fig. 2B, C). Lipid peroxidation level, GSH/GSSG ratio, mRNA of prostaglandin-endoperoxide synthase 2 (PTSG2) and MDA level were induced with AGR2 KO and were inhibited by Lip-1 in p53wt cells, but not in p53mut cells (Fig. 2E, Supplementary fig. 2D–G). To investigate the cytoprotective role of AGR2 in ferroptosis regulation, AGR2 KO combined with TBH treatment induced morphological hallmarks of ferroptosis, including cellular shrinkage and membrane disintegration, in pancreatic cancer cells (Fig. 2G). This result demonstrates that AGR2 functions as a negative regulator of ferroptosis in pancreatic malignancies and is associated with p53 status.

### AGR2 KO inhibits the tumor development of orthotopic PDAC partly through ferroptosis

**Fig. 3:**
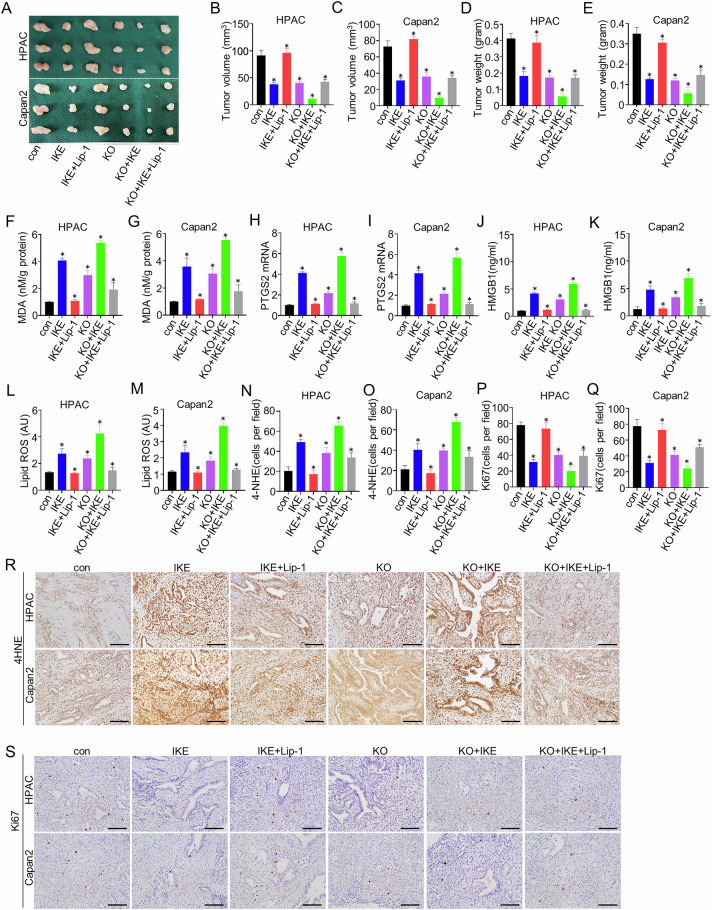
AGR2 inhibits PDAC ferroptosis in vivo. **A** Gross appearance of the tumor in the con, IKE, IKE+Lip- 1, AGR2 KO, AGR2 KO + IKE, and AGR2 KO + IKE+Lip-1 groups of HPAC and Capan2 cells. **B**, **C** Tumor volume of mouse model in the con, IKE, IKE+Lip-1, AGR2 KO, AGR2 KO + IKE, and AGR2 KO + IKE+Lip-1 groups of HPAC and Capan2 cells. **D**, **E** Tumor weight of mouse model in the con, IKE, IKE+Lip-1, AGR2 KO, AGR2 KO + IKE, and AGR2 KO + IKE+Lip-1 groups of HPAC and Capan2 cells. **F**, **G** The bar chart shows the MDA levels of tumors in the con, IKE, IKE+Lip-1, AGR2 KO, AGR2 KO + IKE, and AGR2 KO + IKE+Lip-1 groups of HPAC and Capan2 cells. **H**, **I** The bar chart shows the mRNA expression of PTGS2 of tumors in the con, IKE, IKE+Lip-1, AGR2 KO, AGR2 KO + IKE, and AGR2 KO + IKE+Lip-1 groups of HPAC and Capan2 cells. **J**, **K** The bar chart shows the HMGB1 levels of tumors in the con, IKE, IKE+Lip-1, AGR2 KO, AGR2 KO + IKE, and AGR2 KO + IKE+Lip-1 groups of HPAC and Capan2 cells. **L**, **M** The bar chart shows the Lipid ROS levels of tumors in the con, IKE, IKE+Lip-1, AGR2 KO, AGR2 KO + IKE, and AGR2 KO + IKE+Lip-1 groups of HPAC and Capan2 cells. **N**–**Q** Bar charts show the number of 4-NHE and Ki67 cells of tumors in the con, IKE, IKE+Lip-1, AGR2 KO, AGR2 KO + IKE, and AGR2 KO + IKE+Lip-1 groups of HPAC and Capan2 cells. **R**, **S** Representative IHC staining of 4HNE and Ki67 of tumors in the con, IKE, IKE+Lip-1, AGR2 KO, AGR2 KO + IKE, and AGR2 KO + IKE+Lip-1 groups of HPAC and Capan2 cells. Scale bar = 100 µm. Data are presented as the mean of 3 independent experiments ± SEM. *P < 0.1, **P < 0.01, ***P < 0.001.

To evaluate the therapeutic synergy between ferroptosis induction and AGR2 ablation, we conducted assessments of IKE—a metabolically stabilized derivative of the ferroptosis inducer erastin—in orthotopic pancreatic cancer models. By in vivo results, AGR2 KO or IKE treatment significantly inhibited tumor growth, and the combination of AGR2 KO and IKE treatment significantly suppressed tumor growth (Fig. 3A–E). The MDA levels, the mRNA expression of PTGS2, lipid ROS levels, and HMGB1 protein level were also increased, all of which were inhibited by the ferroptosis inhibitor Lip-1 (Fig. 3F–M). And 4HNE and ki67 staining proved that AGR2 KO and IKE treatment induced ferroptosis and inhibited tumor proliferation, all of which were reversed by the ferroptosis inhibitor Lip-1 (Fig. 3N–S). These in vivo studies supported the hypothesis that AGR2 KO inhibits the tumor development of orthotopic PDAC partly through ferroptosis

### AGR2 acts as a p53 repressor to regulate ferroptosis

**Fig. 4:**
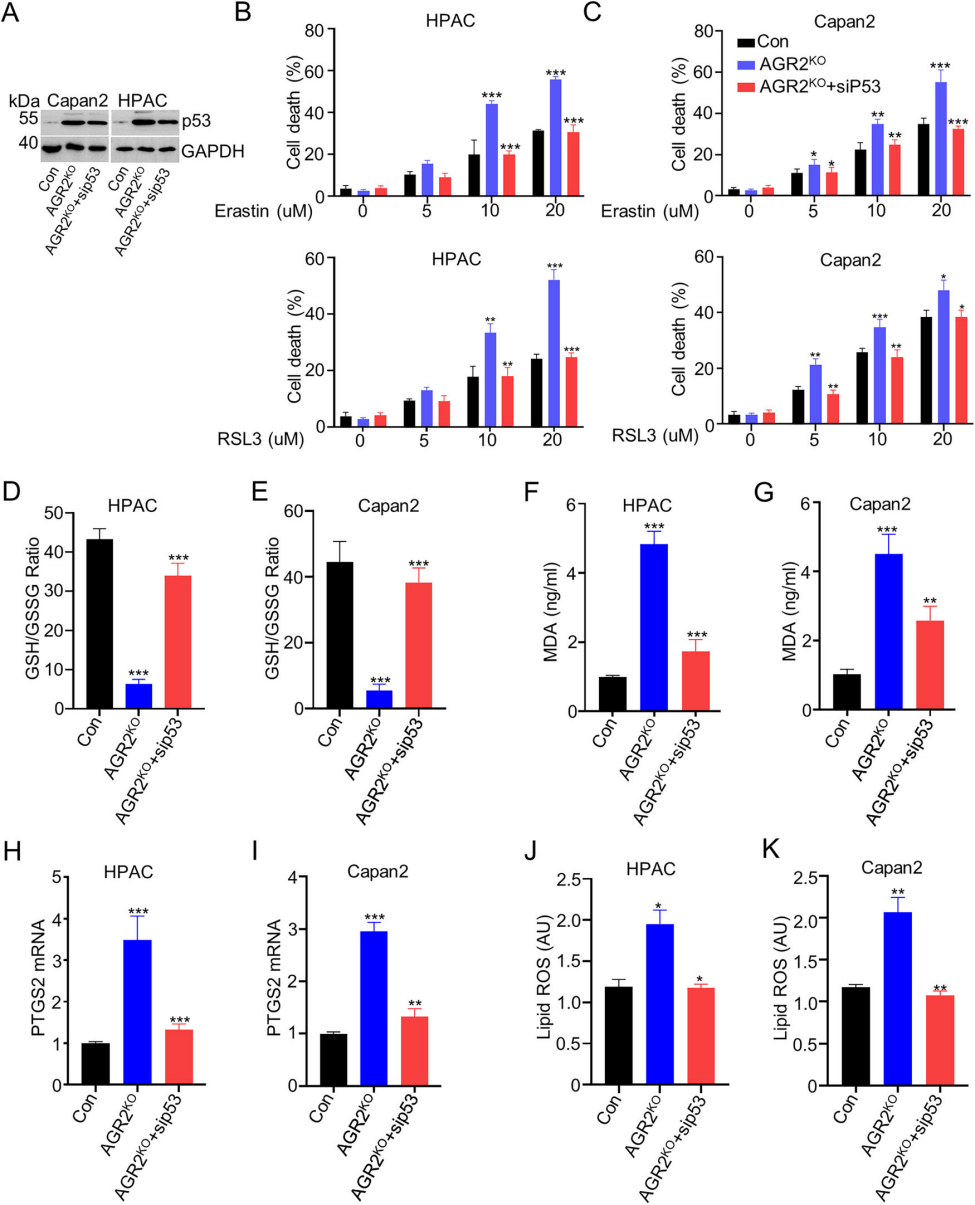
Agr2 inhibits ferroptosis in a p53-independent manner. **A** Western blot analysis shows p53 and GAPDH expression in Con, AGR2 KO, AGR2 KO+sip53 group of HPAC and Capan2 cells. **B**, **C** Quantitative analysis showed the cell death rate of Capan2 and HPAC cells treated with different concentrations of Erastin (0–20 µM) or RSL3 (0–20 µM). **D**, **E** Bar charts show the GSH/GSSG ratios in the AGR2 KO, AGR2 KO+sip53 groups of HPAC and Capan2 cells. **F**, **G** Bar charts show the MDA levels in the AGR2 KO, AGR2 KO+sip53 group of HPAC and Capan2 cells. **H**, **I** Bar charts show the mRNA expression of PTGS2 in the AGR2 KO, AGR2 KO+sip53 groups of HPAC and Capan2 cells. **J**, **K** Bar charts show the lipid ROS levels in the AGR2 KO, AGR2 KO+sip53 groups of HPAC and Capan2 cells. Data are presented as the mean of 3 independent experiments ± SEM. *P < 0.1, **P < 0.01, ***P < 0.001.

Then, we investigated the potential molecular mechanism of AGR2 in ferroptosis. Our study identified p53 as the downstream target of AGR2 [2]. And the KEGG analysis of RNA-sequencing data showed involvement of p53 in ferroptosis (Fig. 2C). Previous studies also identified the role of p53 in ferroptosis [16, 17]. Thus, we hypothesized that AGR2 regulates ferroptosis by p53 pathway. Then, p53 was knocked down (KD) in HPAC and Capan2 cell lines with siRNA (Fig. 4A). As expected, p53 KD rescued RSL3 or erastin-induced cell death in HPAC and Capan2 cells upon erastin or RSL3 treatment (Fig. 4B, C). Lipid peroxidation level, MDA level, PTGS2 mRNA level, and lipid ROS level were induced with AGR2 KO and were reversed by p53 KD (Fig. 4D–K). These results revealed that AGR2 acts as a p53 suppressor to regulate ferroptosis.

### AGR2 inhibits ferroportin expression by modulating p53

**Fig. 5:**
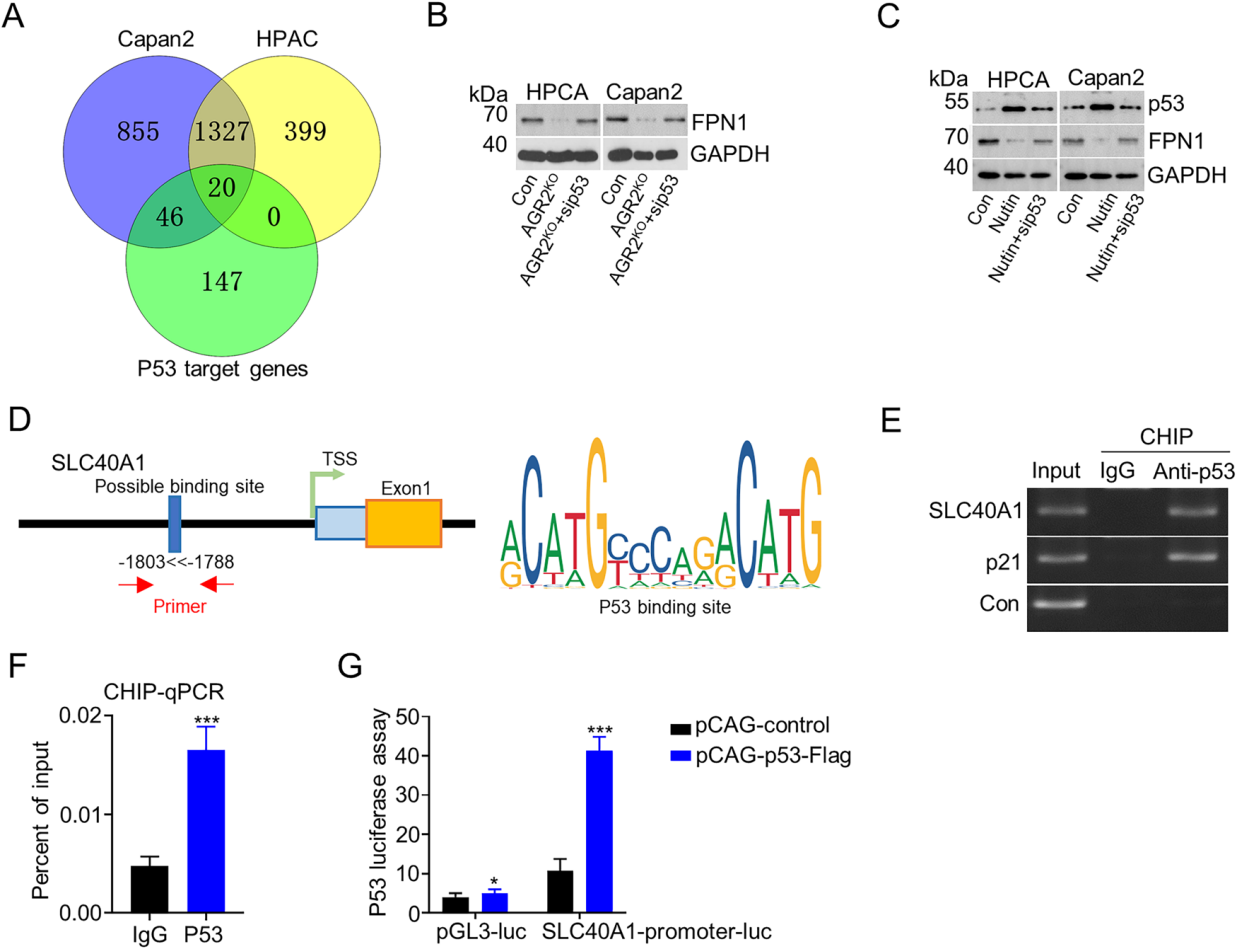
Agr2 inhibit SLC40A1 expression by modulating p53. **A** Venn diagram illustrating the overlap between P53 target genes and two datasets: Capan2 and HPAC. **B** Western blot shows the FPN1 expression in con, AGR2KO, and AGR2KO+sip53 groups of HPAC and Capan2 cells. **C** Western blot shows the p53 and FPN1 expression in con, Nutin, and Nutin+sip53 groups of HPAC and Capan2 cells. **D** Schematic representation of the SLC40A1 promoter region, showing the possible p53 binding site and primer positions. **E** ChIP-PCR analysis shows binding of p53 to the SLC40A1 promoter region in Capan2 cells. **F** Bar chart shows the ChIP-qPCR result of the percentage of input of p53 binding to the SLC40A1 promoter. **G** Luciferase reporter assays demonstrated p53-mediated activation of the SLC40A1 promoter in Capan2 cells transfected with pCAG-control or pCAG-p53-Flag plasmids. Data are presented as the mean of 3 independent experiments ± SEM. *P < 0.1, **P < 0.01, ***P < 0.001.

To elucidate potential mechanisms of the AGR2-p53 axis in regulating ferroptosis in pancreatic cancer models, we re-analyzed transcriptomic data of Capan2 KO cells. Dysregulated genes were investigated. Among these, we focused on SLC40A1 (encoding ferroportin, FPN1), a critical iron exporter (Fig. 5A). Genetic perturbation experiments demonstrated that AGR2 KO significantly suppressed FPN1 expression, while p53 KD effectively rescued FPN1 suppression (Fig. 5B). Pharmacological activation with Nutlin-3a (a p53 activator) confirmed the result, upregulated p53 expression inhibited FPN1 protein level, and this effect was reversed by concurrent P53 knockdown (Fig. 5C). Given that p53’s established role is as a transcription factor, we hypothesized that p53 directly transcriptional transcription of FPN1. Indeed, transcription prediction showed that p53 might bind to the promoter area of SLC40A1 genes (Fig. 5D). Chromatin immunoprecipitation (ChIP) assays validated physical interaction between p53 and the SLC40A1 promoter region (Fig. 5D, E). Subsequent functional validation through dual-luciferase reporter assays demonstrated dose-dependent transcriptional repression of SLC40A1 by p53 (Fig. 5F). Furthermore, the pancreatic transgenic mouse model proved that AGR2 KO significantly induced p53 activation and downregulation of FPN1 (Supplementary fig. 3A–C). And the level of MDA, PTGS2 mRNA level, and HMGB1 level were also upregulated (Supplementary fig. 2D). These results confirmed that AGR2 inhibited FPN1 expression through p53.

### Ferroportin inhibited ferroptosis in pancreatic cancer

**Fig. 6:**
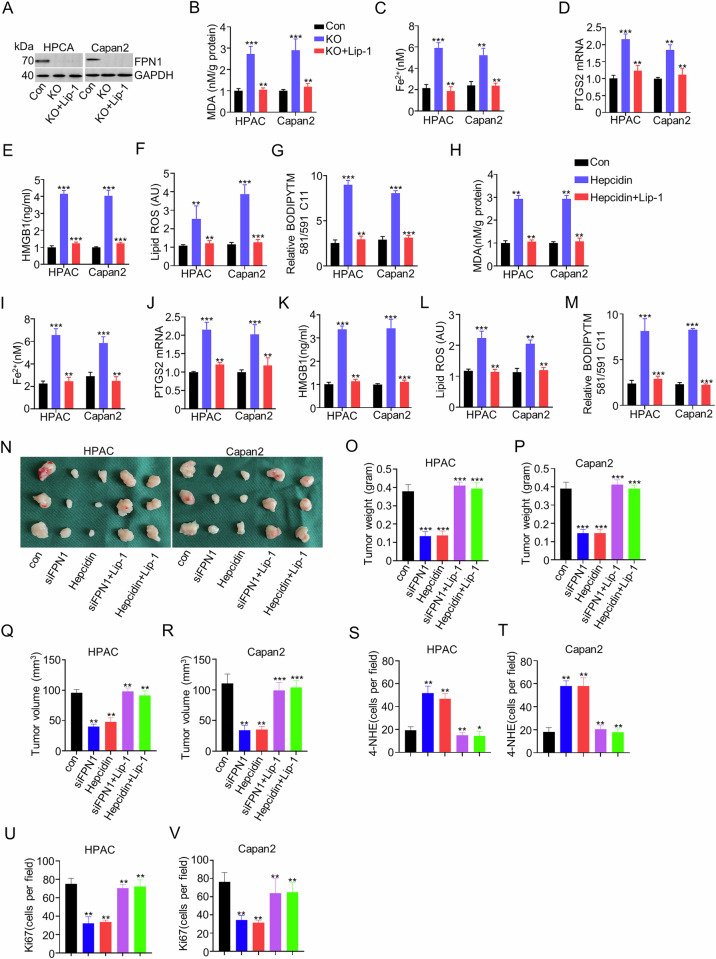
FPN1 inhibition induced iron overload, leading to ferroptosis in pancreatic cancer cells. **A** Western blots show the expression of FPN1, GAPDH in the control and FPN1 knockout group of Capan2 and HPAC cells. **B**–**G** Bar charts show the level of MDA, Ferrous iron, HMGB1, lipid ROS, BODIPYTM 581/591 C11 levels, and the mRNA expression of PTGS2 in control and FPN1 knockout and FPN1 knockout+Lip-1 groups of Capan2 and HPAC cells. **H**–**M** Bar charts show the level of MDA, Iron ion, HMGB1, lipid ROS, BODIPYTM 581/591 C11 levels, and the mRNA expression of PTGS2 in control and hepcidin and hepcidin+Lip-1 groups of Capan2 and HPAC cells. **N** Representative images of tumors from HPAC and Capan2 xenografts in mice under different treatment conditions. **O**, **P** Tumor weight of the tumor in the control, siFPN1, hepcidin, siFPN1+Lip-1, and siFPN1+Lip-1 groups of HPAC and Capan2 cells. **Q**, **R** Tumor volume of the tumor in the control, siFPN1, hepcidin, siFPN1+Lip-1, and siFPN1+Lip-1 groups of HPAC and Capan2 cells. **S**–**V** Bar charts show the number of 4-NHE and Ki67 cells of tumors in the control, siFPN1, hepcidin, siFPN1+Lip-1, and siFPN1+Lip-1 groups of HPAC and Capan2 cells.

Previous studies have identified the role of FPN1 in ferroptosis [18, 19], while it remains unclear in PDAC. Firstly, FPN1 was knocked down in HPAC and Capan2 cells (Fig. 6A). Further analysis proved that FPN1 KD significantly upregulated MDA levels, Fe^2+^(ferrous iron), mRNA expression of PTGS2, HMGB1, and lipid ROS, all of which were reversed by Lip-1 (Fig. 6B-G); In addition, the hepcidin (FPN1 inhibitor) was used. Intrusively, hepcidin significantly induced up-expression of MDA, Fe^2+^, PTGS2, HMGB1, Relative BODIPYTM 581/591 C11, and lipid ROS, all of which were down-regulated after Lip-1 treatment (Fig. 6H–M). We next investigated the function of FPN1 in vivo. Consistent with in vitro results, FPN1 KD or hepcidin treatment blocked tumor growth compared with the control group; this inhibitory effect was reversed by Lip-1 treatment (Fig. 6N–R). Moreover, the further immunohistochemistry staining of 4-HNE and Ki-67 of tumors supported our in vivo results (Fig. 6S–V, Supplementary fig. 4). These preclinical animal studies supported the hypothesis that FPN1 inhibited ferroptosis in pancreatic cancer.

### AGR2 is positively correlated with SLC40A1 expression in pancreatic cancer

**Fig. 7:**
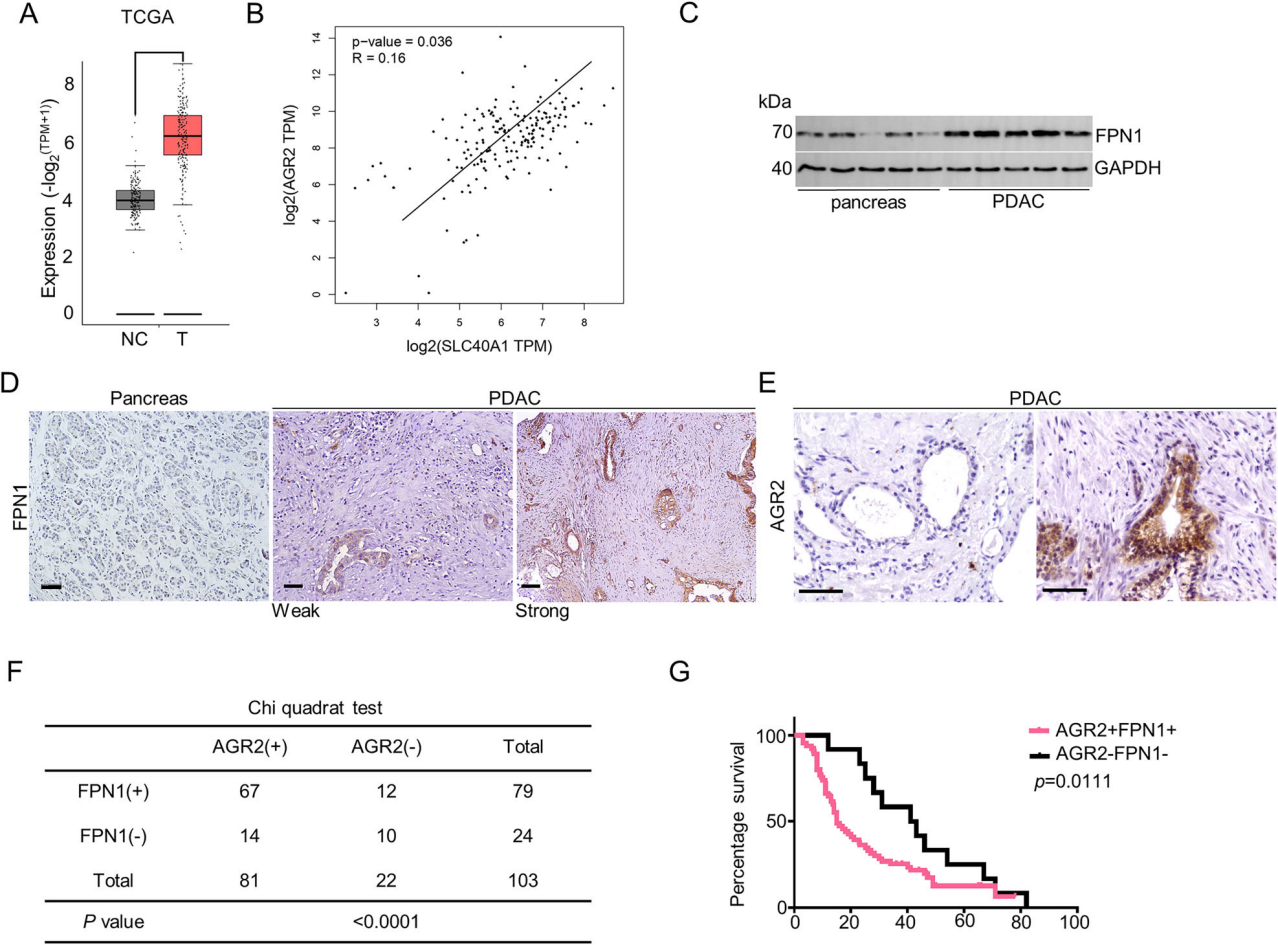
Increased FPN1 expression is associated with poor PDAC patient outcomes. **A** Boxplot shows the expression of FPN1 in normal and PDAC tissues from the TCGA database. **B** Scatter plot shows the correlation between SLC40A1 and AGR2 in PDAC from the TCGA database. **C** Western blot analysis of FPN1 expression in normal pancreas and PDAC tissue. **D** Representative immunohistochemical staining shows the expression of FPN1 in pancreatic and PDAC tissues. Scale bar = 100 µm. **E** Representative immunohistochemical staining shows the expression of AGR2 in PDAC tissues. **F** Chi-square test shows the relationship between FPN1 and AGR2 expression in PDAC. **G** The correlation between AGR2/FPN1 double positive patients and the AGR2/FPN1 double negative group was analyzed.

Building on these findings, this study investigated the co-expression patterns of AGR2 and SLC40A1/FPN1 using both TCGA database analysis and our institutional PDAC patient cohort. The TCGA dataset showed that SLC40A1 was upregulated in PDAC tumors compared with normal pancreas (Fig. 7A). Transcriptomic analysis revealed a positive correlation between AGR2 and SLC40A1 mRNA expression levels (Fig. 7B; Supplementary fig. 5A–C). As 50–70 percent of PDAC patients harbor a mutation in p53, the rest of PDAC patients acquired wild-type p53 [20]. And subgroup analysis also showed positive correlation between two genes independent status of p53 (Supplementary fig. 5D–G). Further immunoblot analysis proved high expression of FPN1 in PDAC tumors (Fig. 7C). Subsequent immunohistochemical validation confirmed a positive correlation of AGR2 and FPN1 expression (Fig. 7D–F). The statues of p53 were listed in the Tables 1, Table 2. All the patients were divided into several groups according to the p53 status. For the patients with wild-type p53, the expression of AGR2 was positively correlated with the expression of the FPN1 (Supplementary fig. 5H); And for the patients with frameshift variant and stop gained p53, the expression of FPN1 did not correlated with AGR2 expression (Supplementary fig. 5I, J), which might due to the small number of patients in this group. For the patients with missense variant p53, the expression of FPN1 did positively correlate with AGR2 expression (Supplementary fig. 5J). Furthermore, survival analysis through Kaplan-Meier curves with log-rank testing demonstrated that high FPN1 and high AGR2 expression were significantly associated with reduced overall survival (OS) in PDAC patients (Fig. 7G). These results indicate that FPN1 is correlated with AGR2 expression in PDAC patients and is a marker for survival.

**Table 1:** The level of AGR2, SLC40A1 and statues of TP53 of PDAC patients.

sample	AGR2	SLC40A1	effect
1	—	+	missense_variant
2	+	+	frameshift_variant
3	+	+	no variant
4	+	+	missense_variant
5	+	+	no variant
6	+	+	stop_gained
7	+	+	missense_variant
8	+	+	no variant
9	-	+	no variant
10	+	+	missense_variant
11	+	+	no variant
12	-	-	no variant
13	+	+	no variant
14	+	+	missense_variant
15	+	+	no variant
16	-	-	frameshift_variant
17	+	-	no variant
18	+	+	missense_variant
19	+	+	no variant
20	-	-	no variant
21	+	+	missense_variant
22	+	+	no variant
23	+	+	stop_gained
24	-	+	missense_variant
25	+	-	no variant
26	+	+	frameshift_variant
27	+	+	missense_variant
28	+	+	no variant
29	+	-	missense_variant
30	+	+	no variant
31	-	+	stop_gained
32	-	+	no variant
33	+	+	missense_variant
34	+	+	no variant
35	+	-	frameshift_variant
36	+	+	missense_variant
37	+	+	no variant
38	-	-	no variant
39	+	+	stop_gained
40	+	-	missense_variant
41	+	+	no variant
42	+	+	no variant
43	-	-	missense_variant
44	+	+	frameshift_variant
45	+	+	missense_variant
46	+	+	no variant
47	-	-	missense_variant
48	+	-	no variant
49	+	+	missense_variant
50	+	+	no variant
51	+	-	frameshift_variant
52	+	+	missense_variant
53	+	+	no variant
54	+	+	missense_variant
55	-	+	no variant
56	-	-	missense_variant
57	+	+	stop_gained
58	-	-	stop_gained
59	+	+	no variant
60	+	+	frameshift_variant
61	+	+	missense_variant
62	+	-	no variant
63	-	+	missense_variant
64	+	+	missense_variant
65	+	+	no variant
66	+	+	stop_gained
67	-	+	no variant
68	+	+	missense_variant
69	+	+	no variant
70	-	+	frameshift_variant
71	+	+	no variant
72	+	+	missense_variant
73	-	-	no variant
74	+	+	no variant
75	+	-	stop_gained
76	+	+	no variant
77	+	+	no variant
78	+	-	missense_variant
79	+	+	frameshift_variant
80	+	+	missense_variant
81	+	-	no variant
82	-	-	missense_variant
83	+	+	missense_variant
84	+	+	no variant
85	+	+	stop_gained
86	+	-	missense_variant
87	+	+	no variant
88	+	+	missense_variant
89	-	-	no variant
90	-	-	missense_variant
91	+	+	no variant
92	+	+	stop_gained
93	-	+	missense_variant
94	+	-	no variant
95	+	+	missense_variant
96	+	+	no variant
97	+	+	frameshift_variant
98	+	+	no variant
99	+	+	missense_variant
100	-	+	no variant
101	+	+	missense_variant
102	+	+	no variant
103	+	+	no variant

**Table 2:** The level of AGR2, and statues of TP53 in PDAC patients from TCGA database.

sample	samples	ENSG00000106541.12 (AGR2)	ENSG00000138449.11 (SLC40A1)	chr	start	end	gene	reference	alt	altGene	effect	aminoAcid	rnaVaf	dnaVaf
TCGA-HZ-7289-01A	TCGA-HZ-7289-01A	13.13	5.933	no variant	no variant	no variant	no variant	no variant	no variant	no variant	no variant	no variant	no variant	no variant
TCGA-FB-AAPP-01A	TCGA-FB-AAPP-01A	11.23	6.123	no variant	no variant	no variant	no variant	no variant	no variant	no variant	no variant	no variant	no variant	no variant
TCGA-US-A776-01A	TCGA-US-A776-01A	11.02	4.859	no variant	no variant	no variant	no variant	no variant	no variant	no variant	no variant	no variant	no variant	no variant
TCGA-HZ-8638-01A	TCGA-HZ-8638-01A	10.38	6.742	no variant	no variant	no variant	no variant	no variant	no variant	no variant	no variant	no variant	no variant	no variant
TCGA-FB-AAPU-01A	TCGA-FB-AAPU-01A	10.01	5.945	no variant	no variant	no variant	no variant	no variant	no variant	no variant	no variant	no variant	no variant	no variant
TCGA-HZ-A8P1-01A	TCGA-HZ-A8P1-01A	9.829	7.076	no variant	no variant	no variant	no variant	no variant	no variant	no variant	no variant	no variant	no variant	no variant
TCGA-IB-A5SP-01A	TCGA-IB-A5SP-01A	9.722	6.662	chr17	7674241	7674241	TP53	G	A		missense_variant	p.S241F		0.51
TCGA-S4-A8RM-01A	TCGA-S4-A8RM-01A	9.637	7.959	no variant	no variant	no variant	no variant	no variant	no variant	no variant	no variant	no variant	no variant	no variant
TCGA-HZ-7924-01A	TCGA-HZ-7924-01A	9.625	7.208	no variant	no variant	no variant	no variant	no variant	no variant	no variant	no variant	no variant	no variant	no variant
TCGA-HV-A7OP-01A	TCGA-HV-A7OP-01A	9.473	6.315	no variant	no variant	no variant	no variant	no variant	no variant	no variant	no variant	no variant	no variant	no variant
TCGA-YY-A8LH-01A	TCGA-YY-A8LH-01A	9.329	6.459	no variant	no variant	no variant	no variant	no variant	no variant	no variant	no variant	no variant	no variant	no variant
TCGA-HZ-A8P0-01A	TCGA-HZ-A8P0-01A	9.315	5.37	no variant	no variant	no variant	no variant	no variant	no variant	no variant	no variant	no variant	no variant	no variant
TCGA-LB-A8F3-01A	TCGA-LB-A8F3-01A	9.288	5.369	no variant	no variant	no variant	no variant	no variant	no variant	no variant	no variant	no variant	no variant	no variant
TCGA-HZ-A9TJ-06A	TCGA-HZ-A9TJ-06A	9.266	5.424	chr17	7675088	7675088	TP53	C	T		missense_variant	p.R175H		0.4317
TCGA-IB-7887-01A	TCGA-IB-7887-01A	9.25	5.839	chr17	7674888	7674888	TP53	T	C		missense_variant	p.S215G		0.1491
TCGA-PZ-A5RE-01A	TCGA-PZ-A5RE-01A	9.244	5.563	chr17	7674950	7674950	TP53	A	T		missense_variant	p.L194H		0.2709
TCGA-3A-A9IZ-01A	TCGA-3A-A9IZ-01A	9.236	7.052	chr17	7674241	7674241	TP53	G	T		missense_variant	p.S241Y		0.3674
TCGA-HZ-A9TJ-01A	TCGA-HZ-A9TJ-01A	9.114	7.359	chr17	7675088	7675088	TP53	C	T		missense_variant	p.R175H		0.4835
TCGA-HZ-8317-01A	TCGA-HZ-8317-01A	9.114	5.838	no variant	no variant	no variant	no variant	no variant	no variant	no variant	no variant	no variant	no variant	no variant
TCGA-FB-AAQ0-01A	TCGA-FB-AAQ0-01A	9.013	5.388	chr17	7674917	7674917	TP53	T	G		missense_variant	p.Y205S		0.4593
TCGA-2L-AAQI-01A	TCGA-2L-AAQI-01A	8.99	6.995	chr17	7673772	7673772	TP53	C	G		missense_variant	p.R283P		0.1818
TCGA-FB-AAPQ-01A	TCGA-FB-AAPQ-01A	8.988	8.095	chr17	7673813	7673814	TP53	GC	-		frameshift_variant	p.S269Ifs		0.1915
TCGA-F2-6879-01A	TCGA-F2-6879-01A	8.926	6.493	chr17	7674872	7674872	TP53	T	C		missense_variant	p.Y220C		0.4552
TCGA-3E-AAAZ-01A	TCGA-3E-AAAZ-01A	8.92	5.966	no variant	no variant	no variant	no variant	no variant	no variant	no variant	no variant	no variant	no variant	no variant
TCGA-IB-7652-01A	TCGA-IB-7652-01A	8.868	7.188	chr17	7675088	7675088	TP53	C	T		missense_variant	p.R175H		0.2318
TCGA-US-A779-01A	TCGA-US-A779-01A	8.806	5.782	chr17	7675055	7675055	TP53	T	-		frameshift_variant;splice_region_variant	p.D186Vfs		0.3975
TCGA-IB-7886-01A	TCGA-IB-7886-01A	8.756	7.159	chr17	7676200	7676201	TP53	-	TT		frameshift_variant	p.D57Kfs		0.4219
TCGA-HV-AA8X-01A	TCGA-HV-AA8X-01A	8.734	7.213	chr17	7674187	7674187	TP53	T	A		missense_variant	p.D259V		0.3982
TCGA-IB-7644-01A	TCGA-IB-7644-01A	8.687	6.928	chr17	7676040	7676040	TP53	C	A		missense_variant	p.R110L		0.4267
TCGA-H8-A6C1-01A	TCGA-H8-A6C1-01A	8.681	6.12	chr17	7676398	7676398	TP53	G	-		frameshift_variant	p.P27Lfs		0.1692
TCGA-IB-7888-01A	TCGA-IB-7888-01A	8.68	5.687	chr17	7674957	7674957	TP53	G	A		stop_gained	p.Q192		0.04281
TCGA-US-A77G-01A	TCGA-US-A77G-01A	8.634	6.728	no variant	no variant	no variant	no variant	no variant	no variant	no variant	no variant	no variant	no variant	no variant
TCGA-HV-A7OL-01A	TCGA-HV-A7OL-01A	8.616	5.719	chr17	7675220	7675220	TP53	T	A		missense_variant	p.N131I		0.2809
TCGA-IB-7889-01A	TCGA-IB-7889-01A	8.611	6.093	chr17	7673728	7673728	TP53	C	A		stop_gained	p.E298		0.09459
TCGA-XD-AAUL-01A	TCGA-XD-AAUL-01A	8.522	5.226	chr17	7675076	7675076	TP53	T	C		missense_variant	p.H179R		0.2197
TCGA-H6-8124-01A	TCGA-H6-8124-01A	8.522	4.974	chr17	7674250	7674250	TP53	C	T		missense_variant	p.C238Y		0.5762
TCGA-HZ-A49I-01A	TCGA-HZ-A49I-01A	8.475	5.828	chr17	7674226	7674226	TP53	A	C		missense_variant	p.M246R		0.1596
TCGA-FB-A78T-01A	TCGA-FB-A78T-01A	8.465	6.559	chr17	7674220	7674220	TP53	C	A		missense_variant	p.R248L		0.2937
TCGA-2J-AAB1-01A	TCGA-2J-AAB1-01A	8.45	6.299	no variant	no variant	no variant	no variant	no variant	no variant	no variant	no variant	no variant	no variant	no variant
TCGA-HZ-A49H-01A	TCGA-HZ-A49H-01A	8.448	6.302	no variant	no variant	no variant	no variant	no variant	no variant	no variant	no variant	no variant	no variant	no variant
TCGA-IB-AAUU-01A	TCGA-IB-AAUU-01A	8.444	6.833	chr17	7676002	7676003	TP53	-	C		frameshift_variant	p.T123Dfs		0.1765
TCGA-2J-AAB8-01A	TCGA-2J-AAB8-01A	8.443	5.971	chr17	7675115	7675115	TP53	G	T		stop_gained	p.S166		0.2059
TCGA-FB-AAPY-01A	TCGA-FB-AAPY-01A	8.443	5.776	chr17	7674240	7674240	TP53	G	-		frameshift_variant	p.C242Afs		0.1123
TCGA-2J-AABF-01A	TCGA-2J-AABF-01A	8.416	6.268	chr17	7674237	7674237	TP53	G	T		stop_gained	p.C242		0.1776
TCGA-IB-A7M4-01A	TCGA-IB-A7M4-01A	8.408	5.129	chr17	7674214	7674214	TP53	G	A		missense_variant	p.P250L		0.3198
TCGA-OE-A75W-01A	TCGA-OE-A75W-01A	8.386	6.142	chr17	7673704	7673704	TP53	G	A		stop_gained	p.R306		0.4112
TCGA-3A-A9IH-01A	TCGA-3A-A9IH-01A	8.375	5.173	chr17	7675184	7675184	TP53	A	T		missense_variant	p.V143E		0.3176
TCGA-2J-AABK-01A	TCGA-2J-AABK-01A	8.35	5.864	chr17	7673805	7673805	TP53	A	G		missense_variant	p.V272A		0.1311
TCGA-Q3-AA2A-01A	TCGA-Q3-AA2A-01A	8.336	7.076	no variant	no variant	no variant	no variant	no variant	no variant	no variant	no variant	no variant	no variant	no variant
TCGA-IB-A5SO-01A	TCGA-IB-A5SO-01A	8.315	5.384	chr17	7674945	7674945	TP53	G	A		stop_gained	p.R196*		0.07619
TCGA-2J-AABH-01A	TCGA-2J-AABH-01A	8.299	5.715	chr17	7676185	7676185	TP53	C	-		frameshift_variant	p.E62Kfs		0.2641
TCGA-FB-AAQ6-01A	TCGA-FB-AAQ6-01A	8.287	7.272	chr17	7674220	7674220	TP53	C	T		missense_variant	p.R248Q		0.2065
TCGA-HZ-7918-01A	TCGA-HZ-7918-01A	8.281	7.203	no variant	no variant	no variant	no variant	no variant	no variant	no variant	no variant	no variant	no variant	no variant
TCGA-HZ-7926-01A	TCGA-HZ-7926-01A	8.265	6.363	chr17	7673717	7673718	TP53	-	G		frameshift_variant	p.G302Rfs		0.1432
TCGA-HV-A5A6-01A	TCGA-HV-A5A6-01A	8.257	5.516	chr17	7670685	7670685	TP53	G	-		frameshift_variant	p.R342Efs		0.4344
TCGA-2J-AABO-01A	TCGA-2J-AABO-01A	8.207	6.441	chr17	7673823	7673823	TP53	C	A		missense_variant	p.G266V		0.3553
TCGA-2L-AAQA-01A	TCGA-2L-AAQA-01A	8.202	6.423	chr17	7675088	7675088	TP53	C	T		missense_variant	p.R175H		0.463
TCGA-IB-7645-01A	TCGA-IB-7645-01A	8.2	6.32	chr17	7674945	7674945	TP53	G	A		stop_gained	p.R196		0.08882
TCGA-2L-AAQJ-01A	TCGA-2L-AAQJ-01A	8.177	7.928	no variant	no variant	no variant	no variant	no variant	no variant	no variant	no variant	no variant	no variant	no variant
TCGA-2J-AABA-01A	TCGA-2J-AABA-01A	8.157	6.76	no variant	no variant	no variant	no variant	no variant	no variant	no variant	no variant	no variant	no variant	no variant
TCGA-HZ-A77O-01A	TCGA-HZ-A77O-01A	8.145	4.469	chr17	7675232	7675232	TP53	G	A		missense_variant	p.S127F		0.3675
TCGA-HZ-8636-01A	TCGA-HZ-8636-01A	8.086	6.271	chr17	7675093	7675094	TP53	-	ACG		inframe_insertion	p.V173dup		0.3162
TCGA-S4-A8RO-01A	TCGA-S4-A8RO-01A	8.071	5.466	chr17	7673776	7673776	TP53	G	A		missense_variant	p.R282W		0.5955
TCGA-FB-AAPZ-01A	TCGA-FB-AAPZ-01A	8.061	5.999	chr17	7674894	7674894	TP53	G	A		stop_gained	p.R213		0.6823
TCGA-IB-7646-01A	TCGA-IB-7646-01A	8.054	4.084	chr17	7674199	7674199	TP53	A	G		missense_variant	p.I255T		0.2689
TCGA-HZ-7919-01A	TCGA-HZ-7919-01A	8.044	6.497	no variant	no variant	no variant	no variant	no variant	no variant	no variant	no variant	no variant	no variant	no variant
TCGA-F2-A8YN-01A	TCGA-F2-A8YN-01A	8.028	6.753	chr17	7674862	7674872	TP53	AGGCGGCTCAT	-		frameshift_variant	p.Y220		0.2294
TCGA-HZ-A4BK-01A	TCGA-HZ-A4BK-01A	7.999	6.844	chr17	7675161	7675161	TP53	G	T		missense_variant	p.P151T		0.1789
TCGA-IB-7647-01A	TCGA-IB-7647-01A	7.963	6.415	chr17	7674221	7674221	TP53	G	A		missense_variant	p.R248W		0.1547
TCGA-YB-A89D-01A	TCGA-YB-A89D-01A	7.892	6.184	no variant	no variant	no variant	no variant	no variant	no variant	no variant	no variant	no variant	no variant	no variant
TCGA-YH-A8SY-01A	TCGA-YH-A8SY-01A	7.884	5.156	chr17	7675088	7675088	TP53	C	T		missense_variant	p.R175H		0.1929
TCGA-FB-A7DR-01A	TCGA-FB-A7DR-01A	7.869	5.897	no variant	no variant	no variant	no variant	no variant	no variant	no variant	no variant	no variant	no variant	no variant
TCGA-IB-AAUW-01A	TCGA-IB-AAUW-01A	7.861	6.598	no variant	no variant	no variant	no variant	no variant	no variant	no variant	no variant	no variant	no variant	no variant
TCGA-S4-A8RP-01A	TCGA-S4-A8RP-01A	7.843	7.348	no variant	no variant	no variant	no variant	no variant	no variant	no variant	no variant	no variant	no variant	no variant
TCGA-HZ-7922-01A	TCGA-HZ-7922-01A	7.813	6.2	chr17	7674945	7674945	TP53	G	A		stop_gained	p.R196		0.3964
TCGA-3A-A9IC-01A	TCGA-3A-A9IC-01A	7.787	5.697	chr17	7676003	7676004	TP53	CA	-		frameshift_variant	p.V122Dfs		0.1633
TCGA-HV-A5A4-01A	TCGA-HV-A5A4-01A	7.755	6.298	no variant	no variant	no variant	no variant	no variant	no variant	no variant	no variant	no variant	no variant	no variant
TCGA-2J-AABR-01A	TCGA-RB-A7B8-01A	7.732	6.549	no variant	no variant	no variant	no variant	no variant	no variant	no variant	no variant	no variant	no variant	no variant
TCGA-RB-A7B8-01A	TCGA-2J-AABR-01A	7.732	6.062	no variant	no variant	no variant	no variant	no variant	no variant	no variant	no variant	no variant	no variant	no variant
TCGA-3A-A9J0-01A	TCGA-3A-A9J0-01A	7.708	6.557	chr17	7675052	7675052	TP53	C	T		splice_donor_variant	p.X187_splice		0.4231
TCGA-HV-A5A3-01A	TCGA-HV-A5A3-01A	7.708	6.315	chr17	7674221	7674221	TP53	G	A		missense_variant	p.R248W		0.3435
TCGA-2J-AABV-01A	TCGA-2J-AABV-01A	7.675	4.747	no variant	no variant	no variant	no variant	no variant	no variant	no variant	no variant	no variant	no variant	no variant
TCGA-F2-A44G-01A	TCGA-F2-A44G-01A	7.664	6.882	no variant	no variant	no variant	no variant	no variant	no variant	no variant	no variant	no variant	no variant	no variant
TCGA-3A-A9IV-01A	TCGA-3A-A9IV-01A	7.659	5.378	no variant	no variant	no variant	no variant	no variant	no variant	no variant	no variant	no variant	no variant	no variant
TCGA-FB-AAQ3-01A	TCGA-FB-AAQ3-01A	7.658	6.711	chr17	7675079	7675080	TP53	-	G		frameshift_variant	p.H178Pfs		0.1934
TCGA-3A-A9I7-01A	TCGA-3A-A9I7-01A	7.637	5.995	no variant	no variant	no variant	no variant	no variant	no variant	no variant	no variant	no variant	no variant	no variant
TCGA-2L-AAQE-01A	TCGA-2L-AAQE-01A	7.624	5.503	chr17	7676257	7676257	TP53	G	A		stop_gained	p.Q38		0.3487
TCGA-IB-7649-01A	TCGA-IB-7649-01A	7.571	6.622	chr17	7675993	7675993	TP53	C	T		splice_donor_variant	p.X125_splice		0.1336
TCGA-HZ-A77P-01A	TCGA-HZ-A77P-01A	7.564	6.47	no variant	no variant	no variant	no variant	no variant	no variant	no variant	no variant	no variant	no variant	no variant
TCGA-2L-AAQL-01A	TCGA-2L-AAQL-01A	7.545	5.585	chr17	7675237	7675237	TP53	C	A		splice_acceptor_variant	p.X126_splice		0.3083
TCGA-HV-AA8V-01A	TCGA-HV-AA8V-01A	7.54	5.467	chr17	7675160	7675160	TP53	G	C		missense_variant	p.P151R		0.07639
TCGA-HZ-8001-01A	TCGA-HZ-8001-01A	7.498	4.842	chr17	7674220	7674220	TP53	C	T		missense_variant	p.R248Q		0.08351
TCGA-2J-AAB4-01A	TCGA-2J-AAB4-01A	7.487	5.558	chr17	7675236	7675236	TP53	A	T		missense_variant;splice_region_variant	p.Y126N		0.1942
TCGA-IB-7654-01A	TCGA-IB-7654-01A	7.421	6.654	chr17	7673776	7673776	TP53	G	A		missense_variant	p.R282W		0.2333
TCGA-IB-AAUM-01A	TCGA-US-A77E-01A	7.404	6.449	no variant	no variant	no variant	no variant	no variant	no variant	no variant	no variant	no variant	no variant	no variant
TCGA-US-A77E-01A	TCGA-IB-AAUM-01A	7.404	5.88	no variant	no variant	no variant	no variant	no variant	no variant	no variant	no variant	no variant	no variant	no variant
TCGA-IB-AAUQ-01A	TCGA-IB-AAUQ-01A	7.398	4.327	chr17	7675077	7675077	TP53	G	A		missense_variant	p.H179Y		0.0963
TCGA-HZ-8315-01A	TCGA-HZ-8315-01A	7.395	6.184	chr17	7675076	7675076	TP53	T	C		missense_variant	p.H179R		0.144
TCGA-US-A774-01A	TCGA-US-A774-01A	7.347	6.27	no variant	no variant	no variant	no variant	no variant	no variant	no variant	no variant	no variant	no variant	no variant
TCGA-3A-A9I5-01A	TCGA-3A-A9I5-01A	7.211	6.342	no variant	no variant	no variant	no variant	no variant	no variant	no variant	no variant	no variant	no variant	no variant
TCGA-IB-AAUP-01A	TCGA-IB-AAUP-01A	7.201	6.045	no variant	no variant	no variant	no variant	no variant	no variant	no variant	no variant	no variant	no variant	no variant
TCGA-IB-AAUN-01A	TCGA-IB-AAUN-01A	7.185	5.677	chr17	7670700	7670700	TP53	G	A		missense_variant	p.R337C		0.4674
TCGA-3E-AAAY-01A	TCGA-3E-AAAY-01A	7.155	6.19	chr17	7673740	7673740	TP53	C	A		stop_gained	p.E294		0.1011
TCGA-IB-7885-01A	TCGA-IB-7885-01A	7.151	6.104	no variant	no variant	no variant	no variant	no variant	no variant	no variant	no variant	no variant	no variant	no variant
TCGA-3A-A9IU-01A	TCGA-3A-A9IU-01A	7.129	5.153	chr17	7676161	7676161	TP53	C	T		missense_variant	p.A70T		0.4317
TCGA-HZ-A4BH-01A	TCGA-HZ-A4BH-01A	7.126	5.482	chr17	7675208	7675208	TP53	C	T		missense_variant	p.C135Y		0.1653
TCGA-LB-A9Q5-01A	TCGA-LB-A9Q5-01A	7.039	5.466	no variant	no variant	no variant	no variant	no variant	no variant	no variant	no variant	no variant	no variant	no variant
TCGA-F2-A44H-01A	TCGA-F2-A44H-01A	7.025	5.304	no variant	no variant	no variant	no variant	no variant	no variant	no variant	no variant	no variant	no variant	no variant
TCGA-XD-AAUI-01A	TCGA-XD-AAUI-01A	6.988	6.079	chr17	7674220	7674220	TP53	C	T		missense_variant	p.R248Q		0.15
TCGA-HV-A5A5-01A	TCGA-HV-A5A5-01A	6.978	6.409	chr17	7674917	7674917	TP53	T	A		missense_variant	p.Y205F		0.09524
TCGA-IB-7891-01A	TCGA-IB-7891-01A	6.977	6.342	chr17	7674229	7674229	TP53	C	A		missense_variant	p.G245V		0.1019
TCGA-XN-A8T5-01A	TCGA-XN-A8T5-01A	6.957	5.802	no variant	no variant	no variant	no variant	no variant	no variant	no variant	no variant	no variant	no variant	no variant
TCGA-2J-AABE-01A	TCGA-2J-AABE-01A	6.895	7.267	no variant	no variant	no variant	no variant	no variant	no variant	no variant	no variant	no variant	no variant	no variant
TCGA-2J-AABU-01A	TCGA-2J-AABU-01A	6.891	4.592											
TCGA-LB-A7SX-01A	TCGA-LB-A7SX-01A	6.854	6.632	chr17	7674894	7674894	TP53	G	A		stop_gained	p.R213		0.4819
TCGA-RB-AA9M-01A	TCGA-RB-AA9M-01A	6.841	6.113	chr17	7673764	7673764	TP53	C	A		stop_gained	p.E286		0.08287
TCGA-FB-A4P6-01A	TCGA-FB-A4P6-01A	6.84	6.413	no variant	no variant	no variant	no variant	no variant	no variant	no variant	no variant	no variant	no variant	no variant
TCGA-HZ-7925-01A	TCGA-HZ-7925-01A	6.835	5.991	chr17	7675207	7675207	TP53	G	T		stop_gained	p.C135		0.122
TCGA-3A-A9IB-01A	TCGA-H6-A45N-01A	6.82	5.929	chr17	7674872	7674872	TP53	T	C		missense_variant	p.Y220C		0.2877
TCGA-H6-A45N-01A	TCGA-3A-A9IB-01A	6.82	5.039	chr17	7673776	7673776	TP53	G	C		missense_variant	p.R282G		0.2328
TCGA-IB-A5SS-01A	TCGA-IB-A5SS-01A	6.813	6.197	chr17	7673776	7673776	TP53	G	A		missense_variant	p.R282W		0.3435
TCGA-XD-AAUG-01A	TCGA-XD-AAUG-01A	6.812	5.286	no variant	no variant	no variant	no variant	no variant	no variant	no variant	no variant	no variant	no variant	no variant
TCGA-IB-8127-01A	TCGA-IB-8127-01A	6.791	5.994	chr17	7673803	7673803	TP53	G	A		missense_variant	p.R273C		0.3065
TCGA-HZ-A49G-01A	TCGA-HZ-A49G-01A	6.786	5.735											
TCGA-FB-A4P5-01A	TCGA-FB-A4P5-01A	6.784	5.326											
TCGA-2J-AAB9-01A	TCGA-2J-AAB9-01A	6.779	5.235	chr17	7676059	7676059	TP53	G	A		stop_gained	p.Q104		0.07821
TCGA-IB-AAUT-01A	TCGA-IB-AAUT-01A	6.764	6.111	no variant	no variant	no variant	no variant	no variant	no variant	no variant	no variant	no variant	no variant	no variant
TCGA-F2-6880-01A	TCGA-F2-6880-01A	6.73	5.119	no variant	no variant	no variant	no variant	no variant	no variant	no variant	no variant	no variant	no variant	no variant
TCGA-IB-A6UF-01A	TCGA-IB-A6UF-01A	6.643	5.393	chr17	7673802	7673802	TP53	C	T		missense_variant	p.R273H		0.4355
TCGA-3A-A9I9-01A	TCGA-3A-A9I9-01A	6.623	5.844	chr17	7674230	7674230	TP53	C	T		missense_variant	p.G245S		0.1255
TCGA-M8-A5N4-01A	TCGA-M8-A5N4-01A	6.549	5.674	no variant	no variant	no variant	no variant	no variant	no variant	no variant	no variant	no variant	no variant	no variant
TCGA-2J-AABT-01A	TCGA-2J-AABT-01A	6.516	6.046	chr17	7674895	7674895	TP53	A	-		frameshift_variant	p.R213Dfs		0.1517
TCGA-IB-A5ST-01A	TCGA-IB-A5ST-01A	6.502	5.291	no variant	no variant	no variant	no variant	no variant	no variant	no variant	no variant	no variant	no variant	no variant
TCGA-IB-AAUO-01A	TCGA-IB-AAUO-01A	6.491	4.945	no variant	no variant	no variant	no variant	no variant	no variant	no variant	no variant	no variant	no variant	no variant
TCGA-RL-AAAS-01A	TCGA-RL-AAAS-01A	6.437	5.791	no variant	no variant	no variant	no variant	no variant	no variant	no variant	no variant	no variant	no variant	no variant
TCGA-FB-A545-01A	TCGA-FB-A545-01A	6.394	4.909											
TCGA-US-A77J-01A	TCGA-US-A77J-01A	6.384	5.39	no variant	no variant	no variant	no variant	no variant	no variant	no variant	no variant	no variant	no variant	no variant
TCGA-IB-A7LX-01A	TCGA-IB-A7LX-01A	6.379	4.647	no variant	no variant	no variant	no variant	no variant	no variant	no variant	no variant	no variant	no variant	no variant
TCGA-FB-A5VM-01A	TCGA-FB-A5VM-01A	6.278	2.935	chr17	7676267	7676267	TP53	G	-		frameshift_variant	p.L35Cfs		0.1435
TCGA-HZ-7923-01A	TCGA-HZ-7923-01A	6.251	6.802	no variant	no variant	no variant	no variant	no variant	no variant	no variant	no variant	no variant	no variant	no variant
TCGA-HZ-A77Q-01A	TCGA-HZ-A77Q-01A	6.135	6.379	chr17	7674221	7674221	TP53	G	A		missense_variant	p.R248W		0.1804
TCGA-3A-A9IX-01A	TCGA-3A-A9IX-01A	6.108	5.921	no variant	no variant	no variant	no variant	no variant	no variant	no variant	no variant	no variant	no variant	no variant
TCGA-IB-A6UG-01A	TCGA-IB-A6UG-01A	6.062	5.02	chr17	7674221	7674221	TP53	G	A		missense_variant	p.R248W		0.1238
TCGA-FB-AAQ2-01A	TCGA-FB-AAQ2-01A	6.055	2.892	chr17	7673788	7673788	TP53	G	A		missense_variant	p.P278S		0.3554
TCGA-XD-AAUH-01A	TCGA-XD-AAUH-01A	5.999	4.956	no variant	no variant	no variant	no variant	no variant	no variant	no variant	no variant	no variant	no variant	no variant
TCGA-F2-A7TX-01A	TCGA-F2-A7TX-01A	5.985	6.251	chr17	7674942	7674942	TP53	C	T		missense_variant	p.V197M		0.1848
TCGA-F2-7273-01A	TCGA-F2-7273-01A	5.984	5.825	no variant	no variant	no variant	no variant	no variant	no variant	no variant	no variant	no variant	no variant	no variant
TCGA-FB-AAQ1-01A	TCGA-FB-AAQ1-01A	5.978	4.895	chr17	7676152	7676153	TP53	-	G		frameshift_variant	p.V73Rfs		0.2356
TCGA-Z5-AAPL-01A	TCGA-Z5-AAPL-01A	5.939	5.538	chr17	7674248	7674248	TP53	T	C		missense_variant	p.N239D		0.03659
TCGA-YB-A89D-11A	TCGA-YB-A89D-11A	5.914	5.317											
TCGA-IB-AAUR-01A	TCGA-IB-AAUR-01A	5.832	5.14	no variant	no variant	no variant	no variant	no variant	no variant	no variant	no variant	no variant	no variant	no variant
TCGA-IB-A5SQ-01A	TCGA-IB-A5SQ-01A	5.707	4.907	no variant	no variant	no variant	no variant	no variant	no variant	no variant	no variant	no variant	no variant	no variant
TCGA-IB-8126-01A	TCGA-IB-8126-01A	5.691	5.945	no variant	no variant	no variant	no variant	no variant	no variant	no variant	no variant	no variant	no variant	no variant
TCGA-IB-7651-01A	TCGA-IB-7651-01A	5.651	7.08	no variant	no variant	no variant	no variant	no variant	no variant	no variant	no variant	no variant	no variant	no variant
TCGA-IB-7890-01A	TCGA-IB-7890-01A	5.641	4.732	chr17	7674936	7674936	TP53	C	A		stop_gained	p.G199		0.356
TCGA-HZ-7920-01A	TCGA-HZ-7920-01A	5.59	5.523	no variant	no variant	no variant	no variant	no variant	no variant	no variant	no variant	no variant	no variant	no variant
TCGA-HZ-8002-01A	TCGA-HZ-8002-01A	5.575	6.312	no variant	no variant	no variant	no variant	no variant	no variant	no variant	no variant	no variant	no variant	no variant
TCGA-HZ-8637-01A	TCGA-HZ-8637-01A	5.549	5.805	chr17	7674230	7674230	TP53	C	T		missense_variant	p.G245S		0.2135
TCGA-IB-AAUS-01A	TCGA-IB-AAUS-01A	5.51	5.194	no variant	no variant	no variant	no variant	no variant	no variant	no variant	no variant	no variant	no variant	no variant
TCGA-2J-AAB6-01A	TCGA-2J-AAB6-01A	5.408	2.911	chr17	7673802	7673802	TP53	C	T		missense_variant	p.R273H		0.4732
TCGA-Q3-A5QY-01A	TCGA-Q3-A5QY-01A	5.386	5.209	no variant	no variant	no variant	no variant	no variant	no variant	no variant	no variant	no variant	no variant	no variant
TCGA-2L-AAQM-01A	TCGA-2L-AAQM-01A	5.372	4.63	no variant	no variant	no variant	no variant	no variant	no variant	no variant	no variant	no variant	no variant	no variant
TCGA-XN-A8T3-01A	TCGA-XN-A8T3-01A	5.168	5.029	chr17	7673803	7673803	TP53	G	A		missense_variant	p.R273C		0.1504
TCGA-HZ-8003-01A	TCGA-HZ-8003-01A	5.112	5.538	no variant	no variant	no variant	no variant	no variant	no variant	no variant	no variant	no variant	no variant	no variant
TCGA-HZ-8005-01A	TCGA-HZ-8005-01A	5.071	3.169	chr17	7673534	7673534	TP53	C	T		splice_donor_variant	p.X331_splice		0.1624
TCGA-F2-7276-01A	TCGA-F2-7276-01A	4.946	5.484	no variant	no variant	no variant	no variant	no variant	no variant	no variant	no variant	no variant	no variant	no variant
TCGA-IB-7897-01A	TCGA-IB-7897-01A	4.772	5.815	no variant	no variant	no variant	no variant	no variant	no variant	no variant	no variant	no variant	no variant	no variant
TCGA-IB-7893-01A	TCGA-IB-7893-01A	4.743	5.569	chr17	7674202	7674202	TP53	A	C		missense_variant	p.I254S		0.2229
TCGA-2J-AABI-01A	TCGA-2J-AABI-01A	4.646	3.14	chr17	7674881	7674882	TP53	-	CC		frameshift_variant	p.V217Gfs		0.3974
TCGA-3A-A9IR-01A	TCGA-3A-A9IR-01A	4.375	4.826	no variant	no variant	no variant	no variant	no variant	no variant	no variant	no variant	no variant	no variant	no variant
TCGA-HZ-8519-01A	TCGA-HZ-8519-01A	4.371	6.685											
TCGA-FB-AAPS-01A	TCGA-FB-AAPS-01A	4.333	4.311	chr17	7675077	7675077	TP53	G	A		missense_variant	p.H179Y		0.07013
TCGA-L1-A7W4-01A	TCGA-L1-A7W4-01A	4.183	1.956											
TCGA-HV-A5A3-11A	TCGA-HV-A5A3-11A	2.692	4.934											
TCGA-3A-A9IO-01A	TCGA-3A-A9IO-01A	2.521	4.445	no variant	no variant	no variant	no variant	no variant	no variant	no variant	no variant	no variant	no variant	no variant
TCGA-H6-8124-11A	TCGA-H6-8124-11A	2.362	5.576											
TCGA-3A-A9IL-01A	TCGA-3A-A9IL-01A	2.079	5.151	no variant	no variant	no variant	no variant	no variant	no variant	no variant	no variant	no variant	no variant	no variant
TCGA-2J-AABP-01A	TCGA-2J-AABP-01A	1.803	4.787	chr17	7674229	7674229	TP53	C	T		missense_variant	p.G245D		0.5785
TCGA-IB-AAUV-01A	TCGA-IB-AAUV-01A	1.739	4.645	no variant	no variant	no variant	no variant	no variant	no variant	no variant	no variant	no variant	no variant	no variant
TCGA-3A-A9IN-01A	TCGA-3A-A9IN-01A	0.7354	4.019	no variant	no variant	no variant	no variant	no variant	no variant	no variant	no variant	no variant	no variant	no variant
TCGA-H6-A45N-11A	TCGA-H6-A45N-11A	0.04768	6.742											
TCGA-3A-A9IJ-01A	TCGA-3A-A9IJ-01A	0.01421	2.288	no variant	no variant	no variant	no variant	no variant	no variant	no variant	no variant	no variant	no variant	no variant
TCGA-3A-A9IS-01A	TCGA-3A-A9IS-01A	0.01164	3.649	no variant	no variant	no variant	no variant	no variant	no variant	no variant	no variant	no variant	no variant	no variant
TCGA-FZ-5920-01A				chr17	7675146	7675158	TP53	GGGTGCCGGGCGG	-		frameshift_variant	p.P152Afs		0.1467
TCGA-FZ-5921-01A				chr17	7675157	7675157	TP53	G	A		missense_variant	p.P152L		0.1717
TCGA-FZ-5919-01A				chr17	7675095	7675095	TP53	C	G		missense_variant	p.V173L		0.1254
TCGA-FZ-5924-01A				chr17	7674220	7674220	TP53	C	T		missense_variant	p.R248Q		0.2948
TCGA-FZ-5922-01A				no variant	no variant	no variant	no variant	no variant	no variant	no variant	no variant	no variant	no variant	no variant
TCGA-FZ-5923-01A				no variant	no variant	no variant	no variant	no variant	no variant	no variant	no variant	no variant	no variant	no variant
TCGA-FZ-5926-01A				no variant	no variant	no variant	no variant	no variant	no variant	no variant	no variant	no variant	no variant	no variant
TCGA-FZ-5919-11A														
TCGA-FZ-5920-11A														
TCGA-FZ-5922-11A														
TCGA-FZ-5923-11A														
TCGA-FZ-5924-11A														
TCGA-FZ-5926-11A														

### AGR2 blocking with peptide sensitized PDAC cells to ferroptosis

**Fig. 8:**
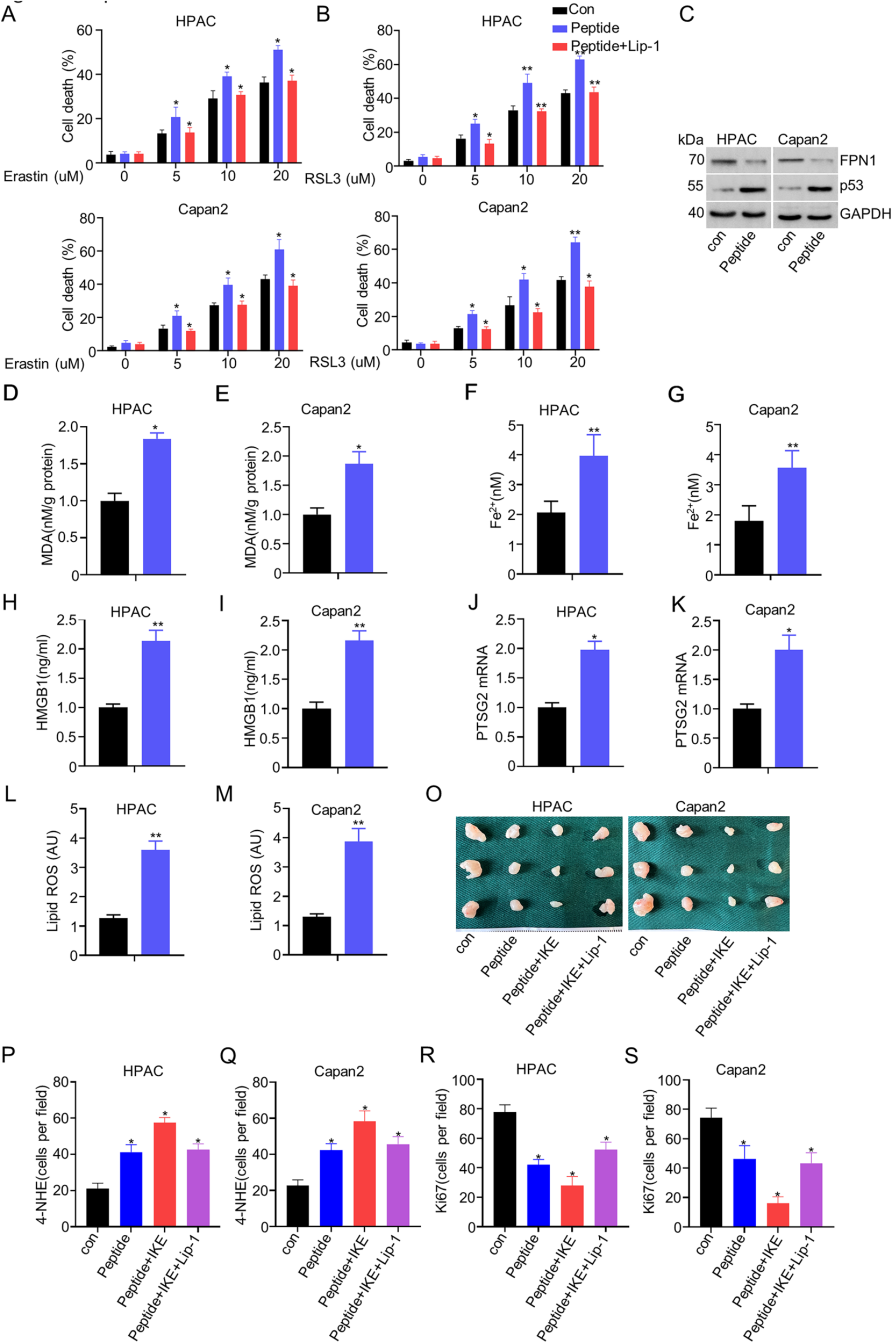
AGR2 blocking with peptide sensitized PDAC cells to ferroptosis. **A**, **B** Bar charts show the cell death percentages in HPAC and Capan2 cells treated with Erastin or RSL3 under different concentrations. **C** Western blot analysis of FPN1, p53, and GAPDH protein levels in HPAC and Capan2 cells. **D**, **E** The bar chart shows the MDA levels in HPAC and Capan2 cells treated with the peptide. **F**, **G** Bar chart shows the Fe²⁺ levels in HPAC and Capan2 cells. **H**, **I** HMGB1 secretion levels in HPAC and Capan2 cells. **J**, **K** PTSG2 mRNA expression in HPAC and Capan2 cells. **L**, **M** Lipid ROS levels in HPAC and Capan2 cells. **O** Representative images of tumor xenografts from HPAC and Capan2 cells under different treatment conditions. **P**–**S** Quantification of 4-NHE and Ki-67 levels in HPAC and Capan2 tumors under different conditions.

Previous results have identified the AGR20-p53-FPN1 axis. We then investigated whether interfering with the AGR2-p53-FPN1 axis might sensitize PDAC to ferroptosis. Our previous study had identified a novel peptide in the treatment of pancreatic carcinogenesis [2]. As expected, peptide treatment increased RSL3 or erastin-induced cell death in HPAC and Capan2 cells (Fig. 8A, B). Immunoblot analysis showed peptide treatment induced upregulation of p53 and downregulation of FPN1 (Fig. 8C). MDA level, Fe^2+^, HMGB1, PTGS2 mRNA levels, and lipid ROS level were also induced with peptide (Fig. 8D–M). In vivo results showed that the peptide significantly inhibited tumor growth and was reversed by Lip-1 treatment (Fig. 7O, Supplementary fig. 6A–D). The immunohistochemistry staining of 4-HNE and Ki-67 of tumors proved that peptide treatment induced upregulation of 4-HNE and proliferative inhibition. And peptide treatment sensitized PDAC cells to IKE treatment, while this effect was reversed by Lip-1 treatment (Fig. 8P–S, Supplementary fig. 6E, F). These results confirmed that the peptide could provide potential therapeutic targets for developing ferroptosis-based interventions against PDAC.

## Discussion

Pancreatic cancer remains one of the most lethal malignancies, characterized by dismal prognosis and the highest mortality rates among solid tumors [1, 21]. The paucity of effective therapeutic interventions constitutes a critical clinical challenge [22], compounded by limited progress in treatment strategies over recent decades. These unmet medical needs underscore the imperative to develop innovative therapeutic approaches. Emerging evidence has established ferroptosis as a promising therapeutic target in pancreatic cancer [23, 24]. Notably, AGR2 has emerged as a multifunctional oncoprotein implicated in tumorigenesis, metastatic progression, chemoresistance, and apoptosis regulation through modulation of carcinogenesis-associated signaling pathways [2, 25]. Nevertheless, the mechanistic interplay between AGR2 and ferroptosis regulation remains to be comprehensively elucidated. In the present study, we showed that ferroptosis induction upregulates AGR2 expression in PDAC cells. Importantly, our findings revealed that AGR2-mediated regulation of the p53/FPN1 axis enhances ferroptosis, positioning AGR2 as a novel regulatory target for modulating this cell death pathway in PDAC.

Ferroptosis is an iron-driven programmed cell death mechanism initiated by lipid peroxidation cascades under glutathione depletion, culminating in iron-mediated lipid peroxidation and subsequent cellular demise [26, 27]. This process is mechanistically governed by two core regulatory axes: systemic iron homeostasis and intracellular redox equilibrium [28]. As an essential micronutrient, iron’s pathophysiological impact is mediated through a network of metabolic regulators, including transferrin receptors, divalent metal transporter 1 (DMT1), and iron regulatory protein 2 (IRP2), which collectively orchestrate cellular iron flux to modulate ferroptotic susceptibility [28]. Malignant progression is frequently associated with reprogrammed iron metabolism pathways encompassing acquisition, storage, utilization, and export mechanisms – adaptations that facilitate tumorigenic iron accumulation [29]. While the complete ferroptosis effector network remains to be fully elucidated, current evidence establishes iron-driven oxidative stress and consequent membrane lipid peroxidation as the central biochemical executors of this cell death pathway [30, 31]. Of relevance, AGR2 has been implicated in cellular homeostasis maintenance through its protein disulfide isomerase activity [32]. In this study, we observed significant AGR2 upregulation in PDAC cells following treatment with canonical ferroptosis inducers (erastin and RSL3). Notably, this induction is proved specific to ferroptotic stimuli, as co-treatment with necroptosis inhibitor NSA or pan-apoptosis inhibitor Z-VAD-FMK failed to reverse AGR2 elevation. CRISPR-mediated AGR2 knockout substantially enhanced PDAC cells’ sensitivity to ferroptosis induction, prompting mechanistic exploration through our established gene expression atlas. Transcriptomic analysis of AGR2-regulated networks revealed p53 is a key nodal point, consistent with its established dual regulatory roles in ferroptosis [14, 15]. Indeed, p53 is a well-known tumor suppressor frequently mutated in PDAC; however, more than 30 percent of PDAC patients maintain WT p53 [33]. Yet, precision therapy targeting p53 in one-third of PDAC cases is not available but desired. While p53 is recognized as a context-dependent ferroptosis modulator capable of both promoting and suppressing cell death [34], our prior work demonstrated AGR2’s oncogenic function through p53 suppression in PDAC carcinogenesis [2]. Currently, we have identified p53 as a potent suppressor of AGR2 knockout-induced ferroptosis sensitization. Mechanistic interrogation uncovered SLC40A1 (encoding ferroportin/FPN1) as a novel p53 transcriptional target, with p53 overexpression significantly repressing FPN1 expression. AGR2-knockout demonstrated concomitant FPN1 downregulation and elevated ferroptosis biomarkers.

FPN1,the exclusive iron export pump in mammals, maintains intracellular iron homeostasis through transmembrane efflux [11, 12]. Its dysfunction induces pathological iron overload through Fenton chemistry-mediated redox cycling between Fe²⁺ and Fe³⁺ [35], generating reactive oxygen species (ROS) that inflict macromolecular damage across DNA, proteins, lipids, and organelles [28]. This iron-dependent oxidative cascade constitutes the biochemical cornerstone of ferroptosis, with cellular iron accumulation shown to potentiate ferroptotic sensitivity across diverse cell types [28]. Consequently, our findings established the AGR2-p53-FPN1 axis as a critical regulatory circuit governing iron homeostasis and ferroptotic vulnerability in PDAC.

To investigate the functional role of FPN1 in pancreatic cancer, we generated FPN1-KO pancreatic cancer cell lines. Genetic ablation of FPN1 triggered significant upregulation of ferroptosis biomarkers and induced intracellular iron overload. Consistent with this finding, treatment with hepcidin - a known FPN1 inhibitor - similarly promoted iron accumulation and enhanced ferroptosis marker expression. Notably, these phenotypic changes were effectively reversed by co-treatment with the ferroptosis inhibitor Lip-1. Complementary in vivo experiments corroborated the tumor-promoting function of FPN1 in pancreatic cancer progression, aligning with prior mechanistic studies [28, 36].

Related clinical findings revealed a high positive correlation between AGR2 and FPN1 expression in pancreatic cancer specimens independent the statues of p53. Survival analysis demonstrated significantly reduced overall survival in patients exhibiting dual high expression of AGR2/FPN1 compared to those with low expression profiles. Moreover, TCGA data was used and analyzed. In total, the mRNA expression of AGR2 was positively correlated with mRNA expression of SLC40A1, and subgroup analysis also showed positive correlation between two genes. This result indicated that AGR2 is correlated with FPN1 (SLC40A1). Despite the key role of p53 in the AGR2/FPN1 axis, there might be potential other mechanisms need further investigation in the future. Mechanistically, our study identified the AGR2/p53/FPN1 regulatory axis as a critical modulator of ferroptosis susceptibility in pancreatic cancer. This novel pathway provides potential therapeutic targets for developing ferroptosis-based interventions against PDAC. And whether the activation of AGR2/p53/FPN1 regulatory axis in other cancers need further investigation.

## Conclusion

In summary, our study demonstrates that inhibiting AGR2-dependent FPN1 expression contributes to ferroptosis resistance in PDAC cells in vitro and in vivo. The expression of AGR2 is positively correlated with FPN1 in pancreatic tumor tissues, which is related to the poor prognosis of pancreatic cancer patients. Therefore, targeting the AGR2/p53/FPN1 pathway may potentially tumor growth and enhance ferroptosis-based therapeutic strategies.

## Supplementary information

Material and Method

Supplementary figure legend

Check list

wb

WB revised

Supplementary figure 1

Supplementary figure 2

Supplementary figure 3

Supplementary figure 4

Supplementary figure 5

Supplementary figure 6

## Data Availability

The data that support the findings of this study are available from the corresponding author upon reasonable request, in accordance with standard procedures.
