# Supplementary material for: AGR2 suppresses ferroptosis via the p53/FPN1 regulatory axis and drives therapeutic vulnerabilities in pancreatic cancer
Source: Cell Death Dis. 2025 Dec 1;16(1):877. doi: 10.1038/s41419-025-08263-y (PMC12669619; doi:10.1038/s41419-025-08263-y)
Supplement: Supplementary file 1 — Material and Method [file 41419_2025_8263_MOESM1_ESM.docx]

**Materials and Methods**

**KEY RESOURCES TABLE**

| REAGENT or RESOURCE | SOURCE | IDENTIFIER |
| --- | --- | --- |
| Table 1 Antibodies | | |
| Primary antibodies | | |
| ACSL4 | Cell Signaling Technology | Cat#4047 |
| GPX4 | Cell Signaling Technology | Cat#52455 |
| xCT/SLC7A11 | Cell Signaling Technology | Cat#98051 |
| AGR2 Polyclonal | Proteintech | Cat#12275-1-AP |
| Beta Actin (AC-15) | SANTA CRUZ | Cat#sc-69879 |
| Anti-Human CD107a(H4A3) | Proteintech | Cat#65051-1-lg |
| GAPDH | Proteintech | Cat#60004-1-lg |
| p53 (1C12) | Cell Signaling Technology | Cat#2524 |
| SLC40A1/FPN1 | Proteintech | Cat#26601-1-AP |
| Ki-67 (D3B5) | Cell Signaling Technology | Cat#34330 |
| 4-Hydroxynonenal | Invitrogen | Cat# MA5-27570 |
| Secondary antibodies | | |
| HRP-Conjugated Goat Anti-Rabbit IgG(H+L) | NCM Biotech | Cat#P8002 |
| HRP-Conjugated Goat Anti-Mouse IgG(H+L) | NCM Biotech | Cat#P8001 |
| Table 2: List of chemicals used in the study | | |
| Liproxstatin-1 | MedChem Express | Cat#HY-12726 |
| Ferrostatin-1 | MedChem Express | Cat#HY-100579 |
| Z-VAD | MedChem Express | Cat#HY-164388 |
| Necrosulfonamide | MedChem Express | Cat#HY-100573 |
| Erastin | MedChem Express | Cat#HY-15763 |
| RSL3 | MedChem Express | Cat#HY-100218A |
| Imidazole ketone erastin | MedChem Express | Cat#HY-114481 |
| Hepcidin | MedChem Express | Cat#HY-P4373 |

| Table 3 Sequences of siRNA | | | | | | | | | |
| --- | --- | --- | --- | --- | --- | --- | --- | --- | --- |
| Name | | | Antisense (5’-3’) | | | | | | |
| AGR2-siRNA#1 | | | TTCTGAGTTAGCAACAAGTAA | | | | | | |
| AGR2-siRNA#2 | | | AAGCTCTATATAAATCCAAGA | | | | | | |
| FPN1-siRNA#1 | | | GCCTTGTTCGGACTGGTCTGTTC | | | | | | |
| FPN1-siRNA#2 | | | CCAGGCATGAACACGGAGATCAC | | | | | | |
| p53-siRNA#1 | | | CCACUACAAGUACAUGUGU | | | | | | |
| p53-siRNA#2 | | | ACACAUGUACUUGUAGUGG | | | | | | |
| Control-siRNA | | | AATTCTCCGAACTGTTCACGT | | | | | | |
| Sequences of sgRNA | | | | | | | | | |
| Name | | | Oligo sequence | | | | | | |
| sgRNA-1 F | | | CTTGATGATTATTCATCACT | | | | | | |
| sgRNA-1 R | | | CTCTATATAAATCCAAGACA | | | | | | |
| Table 4 Sequences of primers | | | | | | | | | |
| Name | | Sense (5’-3’) | | | | | Antisense (5’-3’) | | |
| Mouse | | | | | | | | | |
| Gapdh | | CATCACTGCCACCCAGAAGACTG | | | | | ATGCCAGTGAGCTTCCCGTTCAG | | |
| AGR2 | | GCAGTTTGTTCTCCTCAACCTGG | | | | | GTATCGTCCAGTGATGTCTGCC | | |
| PTGS2 | | GCGACATACTCAAGCAGGAGCA | | | | | AGTGGTAACCGCTCAGGTGTGTTG | | |
| SLC40A1 | | CCATAGTCTCTGTCAGCCTGCT | | | | | CTTGCAGCAACTGTGTCACCGT | | |
| P21 | | GAGCCTGGCTTTCCTCTCAACG | | | | | ACCTTCTCACACAGCCTCAGTC | | |
| HMGB1 | | CCAAGAAGTGCTCAGAGAGGTG | | | | | GTCCTTGAACTTCTTTGGGTCTCT | | |
| Human | | | | | | | | | |
| GAPDH | | GGAGCGAGATCCCTCCAAAAT | | | | | GGCTGTTGTCATACTTCTCATGG | | |
| AGR2 | | GGTGACCAACTCATCTGGACTC | | | | | TGACTGTGTGGGCACTCATCCA | | |
| PTGS2 | | CGGTGAAACTCTGGCTAGACAG | | | | | GCAAACCGTAGATGCTCAGGGA | | |
| SLC40A1 | | GAGACAAGTCCTGAATCTGTGCC | | | | | TTCTTGCAGCAACTGTGTCACAG | | |
| P21 | | GAGCCTGGTTTTCCACTTAACGC | | | | | CAGTCCAGTCTCTTGCCTTAGC | | |
| HMGB1 | | GCGAAGAAACTGGGAGAGATGTG | | | | | GCATCAGGCTTTCCTTTAGCTCG | | |
| Table 5 Gene list of ferroptosis-related genes | | | | | | | | | |
| SLC3A2 | GCLM | | SLC7A11 | | ACSL1 | GPX4 | | SLC39A14 | ACSL6 |
| GSS | ATG7 | | LPCAT3 | | MAP1LC3B | ALOX15 | | TP53 | ACSL3 |
| SAT1 | VDAC3 | | TF | | NCOA4 | VDAC2 | | TFRC | GCLC |
| HMOX1 | PCBP1 | | FTL | | ATG5 | SLC40A1 | | MAP1LC3A | FTH1 |
| ACSL4 |  | |  | |  |  | |  |  |
| Biological Samples | | | | | | | | | |
| Human Pancreatic ductal adenocarcinoma tissue | | | | Zhongda Hospital, Medical School, Southeast University | | | | | |
| Software and Algorithms | | | | | | | | | |
| GraphPad Prism 8 | | | | GraphPad Software | | | | | |
| Origin Lab software | | | | OriginLab | | | | | |
| Photoshop CS6 | | | | Adobe | | | | | |
| Image J | | | | NIH | | | | | |
| ImageLab | | | | ImageLab | | | | | |
| Leica Application Suite X | | | | Leica | | | | | |
| FlowJo | | | | TreeStar | | | | | |

**EXPERIMENTAL MODEL AND SUBJECT DETAILS**

Human samples

Human pancreatic samples were obtained from random individuals who underwent surgical resection at Zhongda Hospital, Medical School, Southeast University (Nanjing, China). The normal tissues were collected from the patients who received Whipple surgery, and the pancreatic ductal adenocarcinoma samples were collected from the eligible patients who received Whipple surgery from August 2015 to December 2022. All procedures that involved human sample collection were approved by the tissue bank of The Ethics Committee of Zhongda Hospital (2024ZDSYLL517-Y01), Southeast University (Nanjing, China). Informed consent was obtained from human participants or their family members.

Mice

C57BL/6 and BALB/c nude mice were purchased from Vital River. KC (LSL-KrasG12D Ptf1αCreERTM/þ) mouse lines were obtained from Jackson Laboratory (Bar Harbor, ME). Male mice were used for all experiments at 6-8 weeks of age. AGR2-/- mice kindly provided by Prof. Dr. Bo. Kong. Mouse breeding and collection of mouse tissue was performed at a specific pathogen-free mouse facility with autoclaved food and water in filter-topped cages and kept on a 12-hour light-dark cycle every day. Experiments were approved by the Animal Ethics Committee of Southeast University (20200407060) by the ethical requirements^37^.

Cell Culture

Capan2, HPAC, and PANC1 cell lines were obtained from ATCC, a reputable supplier of biological materials. All cellular cultures were maintained under standard conditions (37°C, 5% CO2) using DMEM (Wisent, Cat#319-005-CL) containing 10% FBS (Wisent, Cat#086-150) and antibiotic-antimycotic solution (100 U/mL penicillin/100 μg/mL streptomycin; ThermoFisher). Cryopreservation was performed with CELLSAVING freezing vials (NCM, Cat#C40100) at -80°C for long-term biobanking.

Stable cell line construction

siRNA Transfection in Cells

Human siRNA oligonucleotides for AGR2, FPN1, p53, and negative control siRNA were purchased from OriGene, Beijing, China. Transfections were conducted as per the manufacturer’s guidelines. In brief, siRNA was diluted in culture medium lacking serum. Lipofectamine® RNAiMAX (Invitrogen) transfection reagent was combined with the diluted siRNA and mixed by vortexing. Incubate for 5 minutes at room temperature. The complexes were then added to the cells. To evaluate the efficacy of siRNA, immunoblot analysis was performed 72 hours after transfection.

CRISPR/Cas9 System

Two single-guide RNAs (sgRNAs) targeting the human AGR2 gene were cloned into the GV392-CMV-hSpCas9-P2A-Puro lentiviral vector (GeneChem, Shanghai, China). Panc1, HPAC and Capan2 cell lines were transduced with lentiviral particles at an MOI of 100. Following 6 days of selection with 2 μg/mL Puromycin Dihydrochloride (beyotime, ST551), single-cell clones were derived through limiting dilution in 96-well plates to establish monoclonal AGR2 knockout cell lines. Successful gene knockout was confirmed through dual validation methods: Sanger sequencing for genomic DNA analysis and Western blotting for protein expression assessment.

RNA seq

For RNA sequencing of HPAC cells, total RNA was isolated from HPAC cells utilizing the RNeasy Kit (#74104, QIAGEN, Darmstadt, Germany). The integrity of the RNA samples was evaluated using an Agilent Bioanalyzer 4150 (Agilent Technologies, CA, USA). Only samples that met the quality criteria were selected for subsequent library construction. Paired-end libraries were constructed and sequenced on a Novaseq 6000 platform (Illumina, San Diego, CA, USA), producing 150 bp paired-end reads. The clean reads were aligned to the reference genome using HISAT2 in orientation mode. Read counting per gene was performed with FeatureCounts. FPKM values were calculated based on gene length and the number of mapped reads. Differential expression analysis was conducted using DESeq2. Genes with an absolute log2 fold change (|log2FC|) greater than 1 and an adjusted p-value (Padj) less than 0.05 were identified as significantly differentially expressed. And mRNA-seq data have been made publicly available and can be accessed at accessed at <https://github.com/zhangflya/AGR2.git>.

Cell viability assays and inhibitor studies

Cells were seeded onto 12-well plates at a density of 1 × 10^5 cells per well and subsequently treated separately with the medium containing 400nmol/L Hepcidin, 100uM IKE, 30uM lip-1 and peptide(1ug/mL) for 24 h. Cell death was quantified by measuring the activity of released LDH using the Cytotoxicity Detection Kit (LDH) in accordance with the manufacturer’s guidelines. (beyotime: C0019S).

Peptide preparation and cell treatment

The peptide (NTAIYY-Loaded Liposome) was custom-made by ChinaPeptides (Suzhou, China) as described previously^2^. The PDAC cell lines were treated with peptide(1ug/mL) for 24 h. Then, the protein, RNA and further functional experiment were performed.

Histology and immunohistochemistry

Hematoxylin & eosin staining

Staining were performed as described previousl^37^. Briefly, pancreatic specimens underwent immersion-fixation in 4% paraformaldehyde (RT, 24h) followed by standard paraffin embedding protocols. Tissue blocks were sectioned into 3-μm slices using a microtome. Prior to staining, sections were thermally equilibrated at 60°C (60min) and subjected to gradient dehydration reversal through sequential immersion in: Xylene (2×15min), Ethanol series (100%→50%, 5min/step), Distilled H_2_O (5min), Histochemical staining was executed as follows: hematoxylin immersion (3-8min), tap water rinse, acid-alcohol differentiation (1% HCl/EtOH, 10sec), ammonium hydroxide treatment (0.6% NH4OH) ,water bluing, eosin exposure (1-3min) , double distilled H2O wash; Final slide preparation involved progressive dehydration through ascending ethanol concentrations (70%→100%), xylene clearing, and mounting with neutral balsam^37^.

Immunohistochemistry

Staining was performed as described previously^37^. Paraffin sections preconditioned at 60°C underwent xylene-mediated dewaxing (2×15min), ethanol-gradient rehydration (100-50%), and antigen retrieval via microwave-accelerated citrate buffer (pH6.0). Post-cooling demarcation preceded Triton X-100 permeabilization (10min) combined with peroxidase blockade (3% H<sub>2</sub>O<sub>2</sub>). After serum blocking (goat, 60min), sequential immunostaining involved overnight primary Ab (4°C) and HRP-secondary Ab (RT, 60min), visualized by DAB/hematoxylin prior to ethanol-xylene dehydration and resin mounting.

Western Blot

Western blot was performed as described previously^37^. PBS-washed cells were lysed in RIPA buffer containing dual protease/phosphatase inhibitors, ultrasonicated (4°C), and quantified via BCA assay (Beyotime P0010). After normalization and loading buffer addition (Beyotime P0015), 20μg protein aliquots underwent discontinuous electrophoresis (10% gel, 60-120V) and PVDF transfer (300mA). Membranes blocked with 5% milk (2h) were probed sequentially with primary Ab (4°C O/N) and HRP-secondary Ab (RT 1h), visualized using NcmECL Ultra (P10100) on Tanon ChemiDoc MP. Protein bands were visualized with the ChemiDoc MP Imaging System (Tanon).

RNA isolation and Quantitative Polymerase Chain Reaction

Total RNA was isolated from cells or mouse pancreas tissue using the RNeasy Mini Kit, in accordance with the manufacturer’s instructions (beyotime: R0077S). The synthesis of cDNA from total RNA was carried out using a reverse transcription kit, as per the manufacturer’s protocol (TIANGEN BIOTECH: KR118). Gene expression quantification was performed through SYBR Green-based qPCR assays (ChamQ Mix, Q711) employing optimized primer-cDNA amplification systems, with subsequent normalization of target transcripts to housekeeping genes and ΔΔCt computational modeling for differential expression determination.

Chromatin Immunoprecipitation followed by qPCR

Grow cells to the desired confluency and crosslink proteins to DNA by adding 1% formaldehyde (macklin, M813895) and incubating at ambient temperature for 10 minutes. Quench the reaction by incorporating 125 mM glycine, incubating at RT for 5 min. Wash cells with PBS on ice and collect them by centrifugation. ﻿Lyse cells in ChIP lysis buffer (beyotime: P2080S) containing protease inhibitors (beyotime: P1010) and incubate on ice for 10–20 minutes. Sonicate lysates to fragment chromatin to an average size of 200–500 bp. Centrifuge the sample at 12,000 × g for 10 minutes at 4°C, then collect the supernatant. Pre-clear chromatin by incubating with protein A/G magnetic beads (beyotime: P2080S) for 1 hour at 4°C.Incubate the pre-cleared chromatin with the target-specific antibody overnight at 4°C with mild rotation. Incubate for 4 hours at 4°C after adding fresh protein A/G beads. Wash beads sequentially with low-salt, high-salt, LiCl, and TE buffers to remove non-specific binding. Incubate the beads in elution buffer (1% SDS, 0.1 M NaHCO₃) at 65°C for 15 minutes to elute the DNA. Incubate the eluate at 65°C overnight after adding 5 M NaCl. Add RNase A and Proteinase K. Incubate at 45°C for 1 hour. Purify DNA using PCR purification kit (beyotime: D0033).

﻿

Evaluation of AGR2 and FPN1 expression in human tissues

Dual-blind histopathological assessments were conducted by independent pathologists unaware of clinical data. Immunostaining quantification employed a semi-quantitative grading system comprising: ‌Chromogenic Intensity‌ (0: Absent, 1: Faint, 2: Moderate, 3: Intense; ‌Cellular Distribution‌: 0: <5%, 1: 5-25%, 2: 26-50%, 3: 51-75%, 4: >75%.

The immunoreactivity index (IRI) was calculated as the mathematical product of intensity × distribution scores. Specimens were stratified into low-expression (IRI ≤4) and high-expression (IRI >4) cohorts based on cumulative scoring thresholds.

Animal experiments

Subcutaneous Tumor Implantation

Equal numbers of Capan2, Capan2 AGR2KO, Capan2 siFPN1, HPAC, HPAC AGR2KO, HPAC siFPN1 cells were injected into BALB/c nude mice at 6-8 weeks of age. After anesthetizing the mice with isoflurane, 1 × 10^6 cells resuspended in 50 µL of Matrigel were injected into the subcutaneous space of the left flanks. Ensure proper delivery by avoiding leakage and confirming the formation of a small bulge under the skin. Mice were observed on a regular basis and analyzed 21 days later.

In Vivo Treatments

Subcutaneous tumor-bearing mice were randomly allocated into experimental cohorts and subjected to intraperitoneal pharmacological interventions: IKE (35 mg·kg⁻¹), Lip-1 (10 mg·kg⁻¹), Peptide formulation (1 μg·ml⁻¹ in 100 μL physiological carrier). Administration initiated on day 7 post-implantation with dosing intervals of 48 hours between treatments. Terminal analyses were performed at postoperative day 24, encompassing therapeutic efficacy evaluation and molecular characterization.

Tumor volume measure

Tumor dimensions (L=maximum, W=perpendicular diameter) were serially measured for volumetric calculation (V=0.5L×W²). Murine biometrics including body mass trajectories were tracked triweekly. Humane experimental endpoint parameters (tumor volume ≥15% body weight or 20% weight loss) triggered CO₂ euthanasia per AAALAC standards.

Staining for lipid peroxidation in cells by flow cytometry

To analyze lipid peroxidation, cells were seeded in 10 cm dishes and cultured in DMEM supplemented with 10% FBS. They were then treated for specified durations. After incubation, the cells were washed, and BODIPY 581/591 C11 (Beyotime, S0043S) was added for 1 hour to stain for lipid peroxidation. The cells were trypsinized, washed, and resuspended in HBSS containing 2% FBS before being transferred to FACS tubes for flow cytometry analysis using the BD FACS Canto II.

Detection of Fe2+ using flow cytometry

We seeded cells in 6-well plates at a density of 200,000 cells per well. Once confluent, the cells were treated with SI and maintained under hypoxic conditions (3% O₂) for 8 hours. For Fe²⁺ quantification, we stained cell pellets with 10 µM Rhodamine B benzyl ester (Aladdin, 121393-15-5) for 12 minutes, washed them with PBS, and then incubated them in PBS for 15 minutes at 37°C. The mean fluorescence intensity (MFI) was detected by flow cytometry BD FACS Canto II). In brief, RPA fluorescence is quenched by Fe2+ allowing the determination of iron reduction.

Plasmid transfection and p53-luciferase reporter assay

The SLC40A1 promoter sequence was cloned into the pGL3-luc vector (Promega，Beijing) to generate the SLC40A1-promoter-luc reporter construct. Capan2 cells were maintained in DMEM with 10% FBS at 37°C with 5% CO₂. Cells were seeded into 24-well plates at approximately 70% confluency to ensure optimal transfection conditions. Transfection was carried out using Lipofectamine 3000 (Thermo Fisher Scientific) following the manufacturer’s protocol to achieve optimal results. Reporter plasmids (pGL3-luc or SLC40A1-promoter-luc), pCAG-control or pCAG-p53-Flag expression plasmids, The Renilla luciferase plasmid (pRL-TK, Promega) was used as an internal control. ﻿A total of 500 ng of DNA was used per well, with a 3:1 Lipofectamine-to-DNA ratio. We lysed the cells at 48 hours post-transfection with Passive Lysis Buffer (Promega). We measured the activities of Firefly and Renilla luciferase using the Dual-Luciferase Reporter Assay System (Promega) on a luminometer(Promega GloMax).

Determination of intracellular GSH/GSSH levels

Homogenize cells (1 × 10⁶) in 500 µL ice-cold PBS. Add 5% SSA (1:1 volume) to remove proteins. Vortex for 10 seconds to ensure uniform mixing, incubate on ice for 10 minutes to maintain low temperature conditions, and centrifuge at 12,000 × g for 10 minutes at 4°C to separate the components. Collect the supernatant for GSH/GSSG quantification. Prepare reaction mixture in a 96-well plate, incubate at room temperature for 5–10 min. Measure absorbance at 412 nm (colorimetric). Calculate total glutathione concentration using a standard curve. Add N-ethylmaleimide (NEM, 10 mM final concentration) to the sample and incubate at room temperature for 15 min to block GSH. Centrifuge and collect the supernatant. Prepare reaction mixture (same as above). Measure absorbance or fluorescence as described. Determine GSSG concentration using a standard curve. Prepare a series of GSH and GSSG dilutions (0–50 µM) in assay buffer. Plot absorbance vs. concentration to generate a standard curve. Use the equation C = (Absorbance - Blank) / Slope to determine sample concentrations. [GSH] = Total Glutathione - 2 × [GSSG], GSH/GSSG Ratio = [GSH] / [GSSG] (beyotime: S0053).

MDA

Resuspend 1 × 10⁶ cells in 500 µL PBS and sonicate. Centrifuge the sample at 12,000 × g for 10 minutes at 4°C to ensure proper separation, and then collect the supernatant for subsequent MDA measurement. Prepare MDA standard by hydrolyzing 1,1,3,3-Tetraethoxypropane (TEP) in HCl (0.6 M) for 10 min at 60°C. Prepare reaction mixture in a 96-well plate, dilute hydrolyzed MDA in PBS to prepare standards (0–20 µM). Incubate at 95°C for 60 minutes in a water bath. Cool the samples on ice to stabilize the reaction products, then centrifuge at 12,000 × g for 5 minutes. Transfer the supernatant to a cuvette or 96-well plate for subsequent analysis. Measure absorbance at 532 nm (colorimetric) or fluorescence at Ex/Em = 530/550 nm. Plot absorbance vs. MDA concentration (µM). Use the equation C = (Absorbance - Blank) / Slope to determine sample MDA levels (beyotime: S0131S).

Lipid ROS

Seed cells into 6-well plates at 5 × 10⁵ cells/well. Incubate at 37°C, 5% CO₂ overnight until they reach 70–80% confluency. Control: Incubate cells in regular media. Lipid ROS Induction: Treat with TBHP (75 µM, 1–4 hours) or another oxidative stress inducer. Dilute C11-BODIPY 581/591 (1 mM stock in DMSO) to 2 µM final concentration in pre-warmed serum-free medium or HBSS. The culture medium was removed and replaced with the C11-BODIPY working solution. Incubate for 30 min at 37°C, 5% CO₂, protected from light. Wash cells twice with PBS to remove excess probe. Trypsinize cells resuspend in PBS, and transfer to FACS tubes. Set up flow cytometer: Red fluorescence (non-oxidized C11-BODIPY): 590–620 nm; Green fluorescence (oxidized lipid ROS): 510–530 nm. Acquire at least 10,000 events per sample. Data Analysis: Assess the ratio of green fluorescence (oxidized form) to red fluorescence (reduced form). A higher green/red ratio indicates increased lipid ROS levels (Thermo Fisher, Invitrogen D3861).

AGR2 and FPN1 correlation analysis

Obtain AGR2 and FPN1 expression data from publicly available databases: The Cancer Genome Atlas (TCGA) data is accessible via <https://portal.gdc.cancer.gov/>. Search for bulk RNA-seq or microarray datasets containing both AGR2 and FPN1 expression (GSE28735, GSE36924, GSE57495). Using R (4.0.2) to calculate correlation coefficients between AGR2 and FPN1 by Pearson correlation (linear relationship) and Spearman correlation (rank-based, non-parametric). Generate scatter plots with trend lines.

Venn Analysis

Venn analysis was performed using BioVenn, a web application for the comparison and visualization of biological lists using area-proportional Venn diagrams (http://www.biovenn.nl/). Microarray data were uploaded to BioVenn for analysis.

TCGA data analysis

Obtain survival data and gene expression values from TCGA (The Cancer Genome Atlas): <https://portal.gdc.cancer.gov/>. Retrieve FPN1 gene expression data for the pancreatic cancer. Ensure data includes both normal control (NC) and tumor (T) samples. Normalize the gene expression data and filter out lowly expressed genes or those with missing values.

Convert expression values to a suitable scale (TPM/FPKM). Perform differential expression analysis to identify genes with significant differences between NC and Tumor. Use statistical tests (e.g., t-test, Wilcoxon rank-sum test) to determine significance and use a box plot to visualize the distribution of FPN1 expression levels between NC and T samples.

Survival analysis

Obtain survival data and gene expression values from TCGA (The Cancer Genome Atlas): <https://portal.gdc.cancer.gov/>. Remove samples with missing survival time or expression data. Convert survival times into a standardized format (months). Samples were divided into high-expression and low-expression groups based on the median expression cutoff to analyze differential expression. The Kaplan-Meier (KM) method estimates survival probabilities for AGR2+FPN1+ and AGR2-FPN1- groups.

Data presentation and statistical analyses

Statistical computations were performed through GraphPad's analytical suite (v7.0) utilizing: Independent-sample t-distribution comparisons, predefined significance criterion (α=0.05, two-sided), central tendency presentation: M ± SEM.
