# Supplementary material for: AGR2 suppresses ferroptosis via the p53/FPN1 regulatory axis and drives therapeutic vulnerabilities in pancreatic cancer
Source: Cell Death Dis. 2025 Dec 1;16(1):877. doi: 10.1038/s41419-025-08263-y (PMC12669619; doi:10.1038/s41419-025-08263-y)
Supplement: Supplementary file 2 — Supplementary figure legend [file 41419_2025_8263_MOESM2_ESM.docx]

**Supplementary Figure 1: AGR2 knockout induces ferroptosis.**

1. Bar charts show the cell death percentages in PANC1, Miapaca2 and AsPC-1 cells treated with Erastin under different concentrations.
2. Bar charts show the cell death percentages in PANC1, Miapaca2 and AsPC-1 cells treated with RSL3 under different concentrations.
3. Quantitative analysis showed the AGR2 mRNA expression of PANC1, Miapaca2 and AsPC-1 cells treated with different concentrations of Erastin.
4. Quantitative analysis showed the AGR2 mRNA expression of PANC1, Miapaca2 and AsPC-1 cells treated with different concentrations of RSL3.
5. Protein expression levels of ACSL4, GPX4, SLC7A11 and AGR2 in Miapaca2,PANC1 and AsPC-1 cells treated with Erastin, RSL3 and Lip-1.
6. Protein expression levels of AGR2 in NC, KC14d, KC and KPC cells .
7. Representative immunohistochemical staining shows the expression of Agr2 in KC 14d and KPC tissues. Scale bar=100 µm.
8. Quantitative analysis showed the relative AGR2 expression levels of different p53 mutation statuses in TCGA dataset.

Data are presented as the mean of 3 independent experiments ± SEM. *P<0.1, **P<0.01, ***P<0.001.

**Supplementary Figure 2: AGR2 knockout induces ferroptosis.**

1. Western blot analysis demonstrated expression of AGR2 protein in AGR2 knockout groups compared to control cells in Miapaca2 and AsPC-1 cells;
2. Bar charts show the cell death percentages in PANC1, Miapaca2 and AsPC-1 cells treated with Erastin under different concentrations.
3. Bar charts show the cell death percentages in PANC1, Miapaca2 and AsPC-1 cells treated with RSL3 under different concentrations.
4. Bar charts show the ratio of GSH and GSSG in AGR2 knockout groups compared to control cells in HPAC, Capan2, PANC1, Miapaca2 and AsPC-1 cells.
5. Bar charts show the MDA levels in AGR2 knockout groups compared to control cells in HPAC, Capan2, PANC1, Miapaca2 and AsPC-1 cells.
6. Bar charts show the PTGS2 mRNA expression in AGR2 knockout groups compared to control cells in HPAC, Capan2, PANC1, Miapaca2 and AsPC-1 cells.
7. Bar charts show the Liqid ROS levels in AGR2 knockout groups compared to control cells in HPAC, Capan2, PANC1, Miapaca2 and AsPC-1 cells.

Data are presented as the mean of 3 independent experiments ± SEM. *P<0.1, **P<0.01, ***P<0.001.

**Supplementary Figure3: AGR2 knockout induce ferroptosis.**

1. H&E staining of histological sections from KC (wild type) and KC;Agr2^-/-^ (Agr2 knockout) pancreatic tissues.Scale bar= 100 µm.
2. Protein expression levels of FPN1, GPX4, p53, SLC7A11 and GAPDH in KC and KC;Agr2^-/-^ cells.
3. Representative immunohistochemical staining of FPN1 in KC and KC;Agr2^-/-^ pancreatic tissues.Scale bar=100 µm.
4. Bar charts show the MDA levels in KC and KC;Agr2^-/-^ cells.
5. Bar charts show the PTGS2 mRNA expression in KC and KC;Agr2^-/-^ cells.
6. Bar charts show the HMGB1 protein level in KC and KC;Agr2^-/-^ cells.

**Supplementary Figure 4: AGR2 knockout induces ferroptosis.**

(A-B) Representative immunohistochemical staining of 4-HNE and Ki67 in the control, siFPN1, Hepcidin, siFPN1+Lip-1, and Hepcidin+Lip-1 groups of HPAC and Capan2 cells. Scale bar=100 µm.

**Supplementary Figure 5.**

(A–C) Scatter plots show the correlation between AGR2 and SLC40A1 in PDAC patients from the GEO datasets GSE28735, GSE36924, and GSE57495.

(D-G) Correlation between AGR2 and SLC40A1 expression levels in pancreatic cancer samples from The Cancer Genome Atlas (TCGA) database, stratified by TP53 mutation status.

(H-K) Chi-squared tests summarize the association between AGR2 and FPN1 expression across different p53 mutation statuses in TCGA samples.

**Supplementary Figure 6:**

(A-D) Tumor volume and weight measurements in HPAC and Capan2 cells xenografts under different conditions.

(E-F) Representative immunohistochemical staining of 4-HNE and Ki67 in HPAC and Capan2 cells xenograft tumors under different treatment conditions. Scale bar=100 µm.
