# Supplementary material for: AGR2 suppresses ferroptosis via the p53/FPN1 regulatory axis and drives therapeutic vulnerabilities in pancreatic cancer
Source: Cell Death Dis. 2025 Dec 1;16(1):877. doi: 10.1038/s41419-025-08263-y (PMC12669619; doi:10.1038/s41419-025-08263-y)
Supplement: Supplementary file 4 — wb [file 41419_2025_8263_MOESM4_ESM.docx]

Fig1


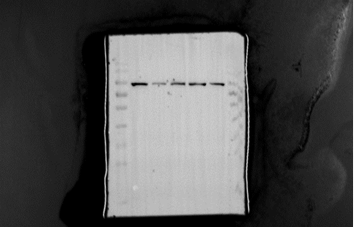


HPAC（ACSL4）


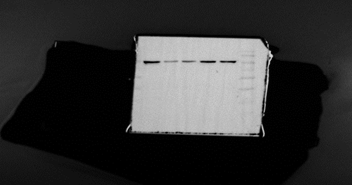


Capan2（ACSL4）


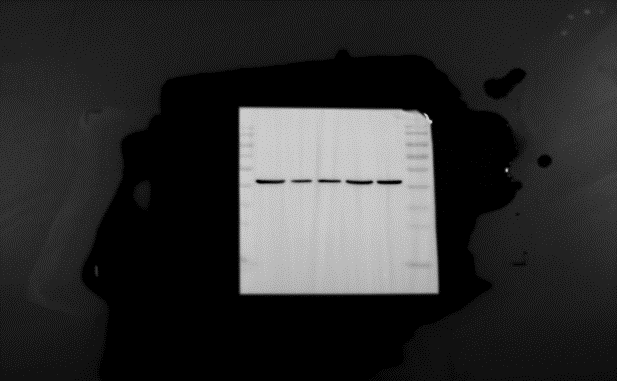


HPAC（GPX4）


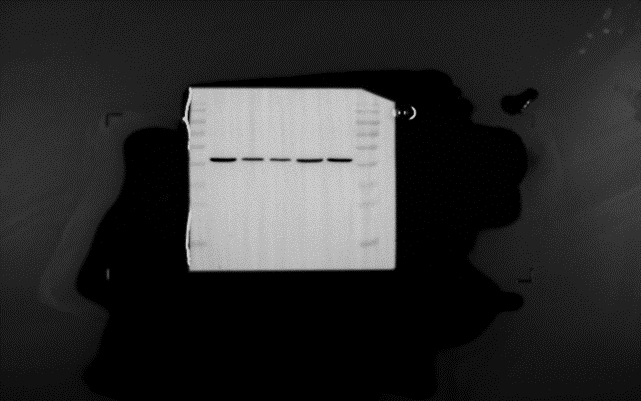


Capan2（GPX4）


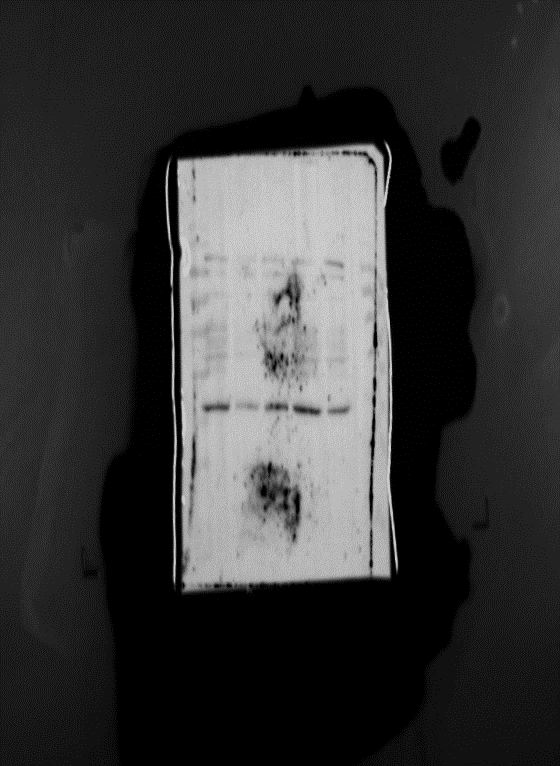


HPAC（SLC7A11）


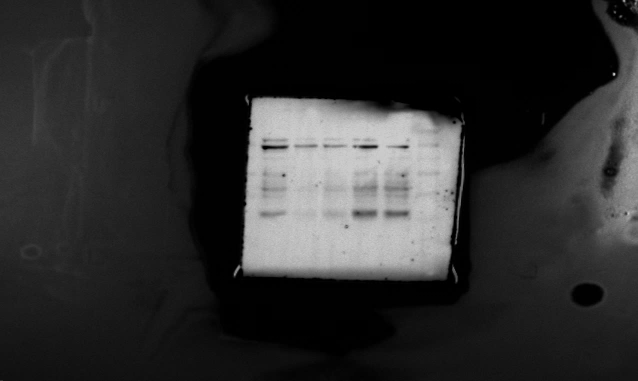


Capan2（SLC7A11）


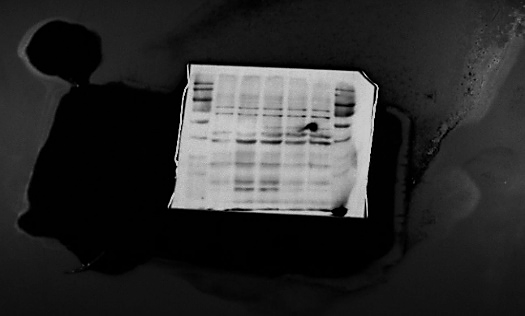


HPAC（AGR2）


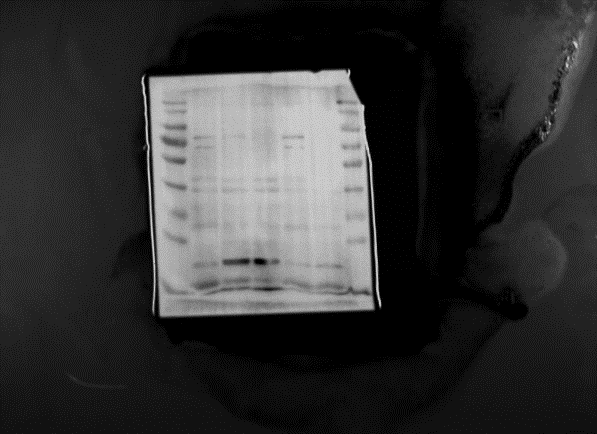


Capan2（AGR2）


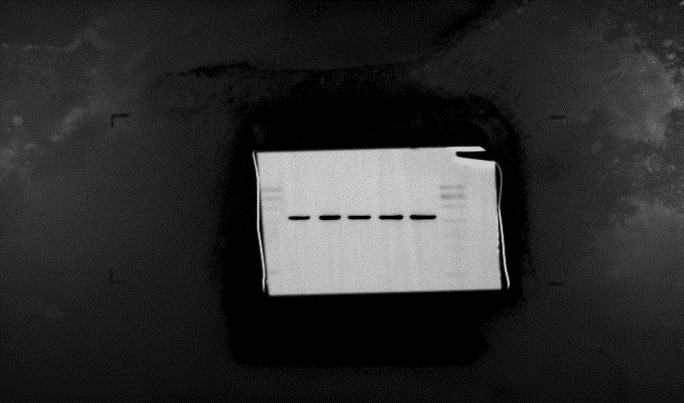


HPAC（β-actin）


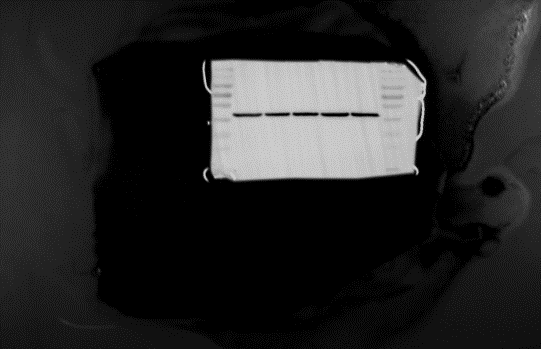


Capan2（β-actin）

Fig2


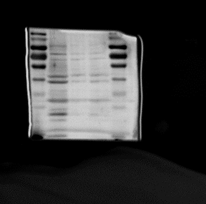


Capan2（AGR2）


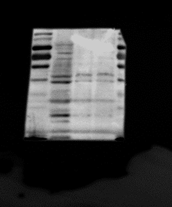


HPAC（AGR2）


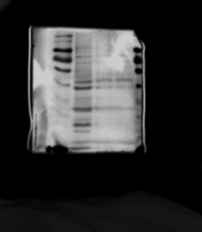


Panc1（AGR2）


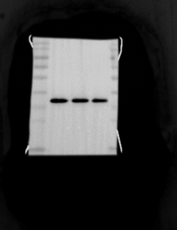


Capan2（GAPDH）


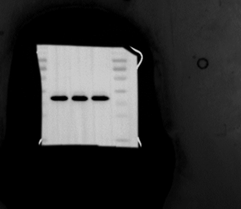


HPAC（GAPDH）


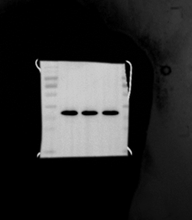


Panc1（GAPDH）

Fig4


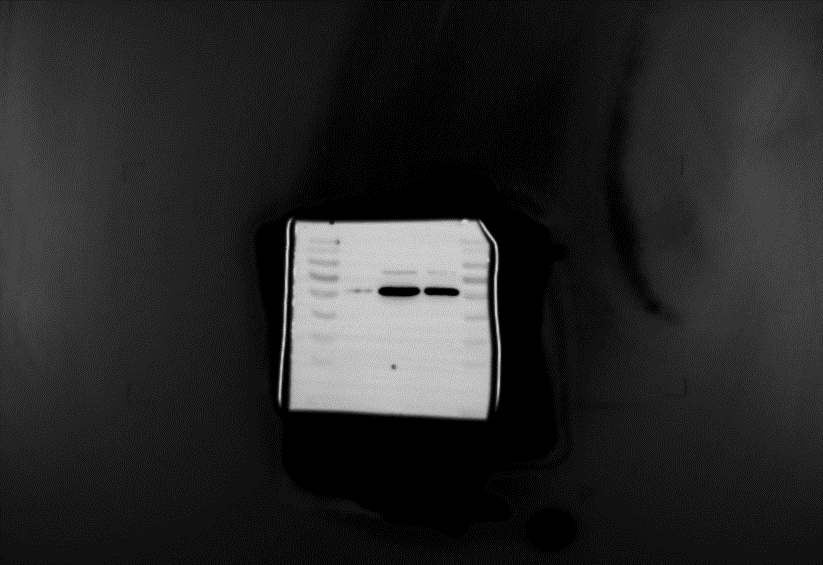


Capan2（p53）


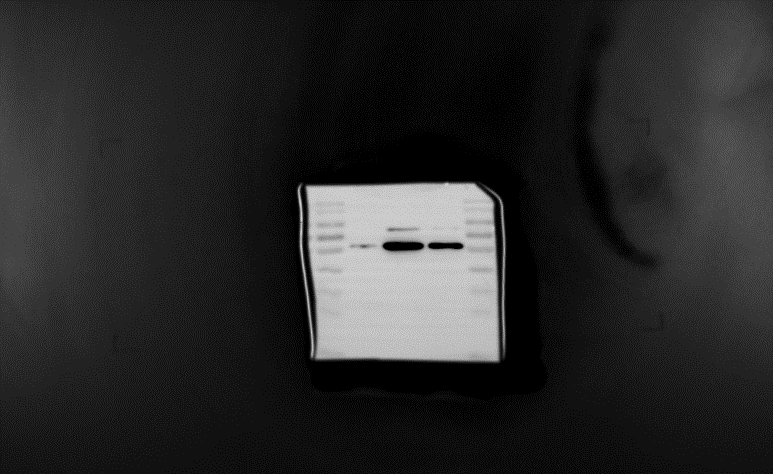


HPAC（p53）


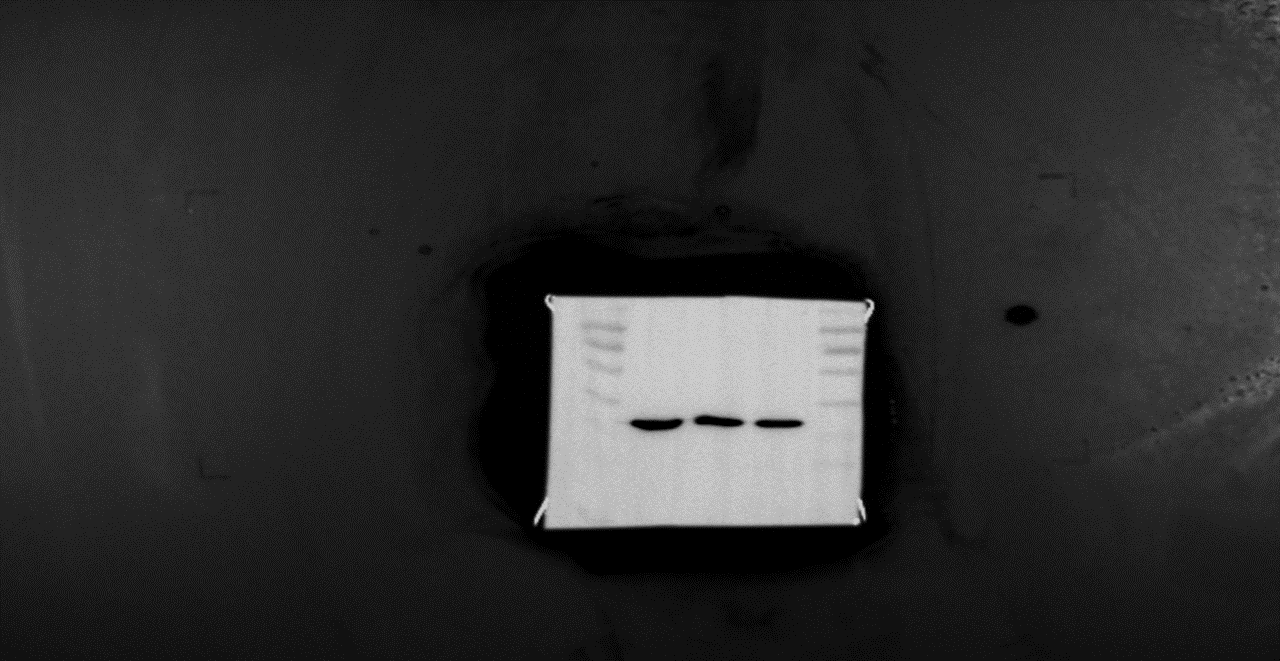


Capan2（GAPDH）


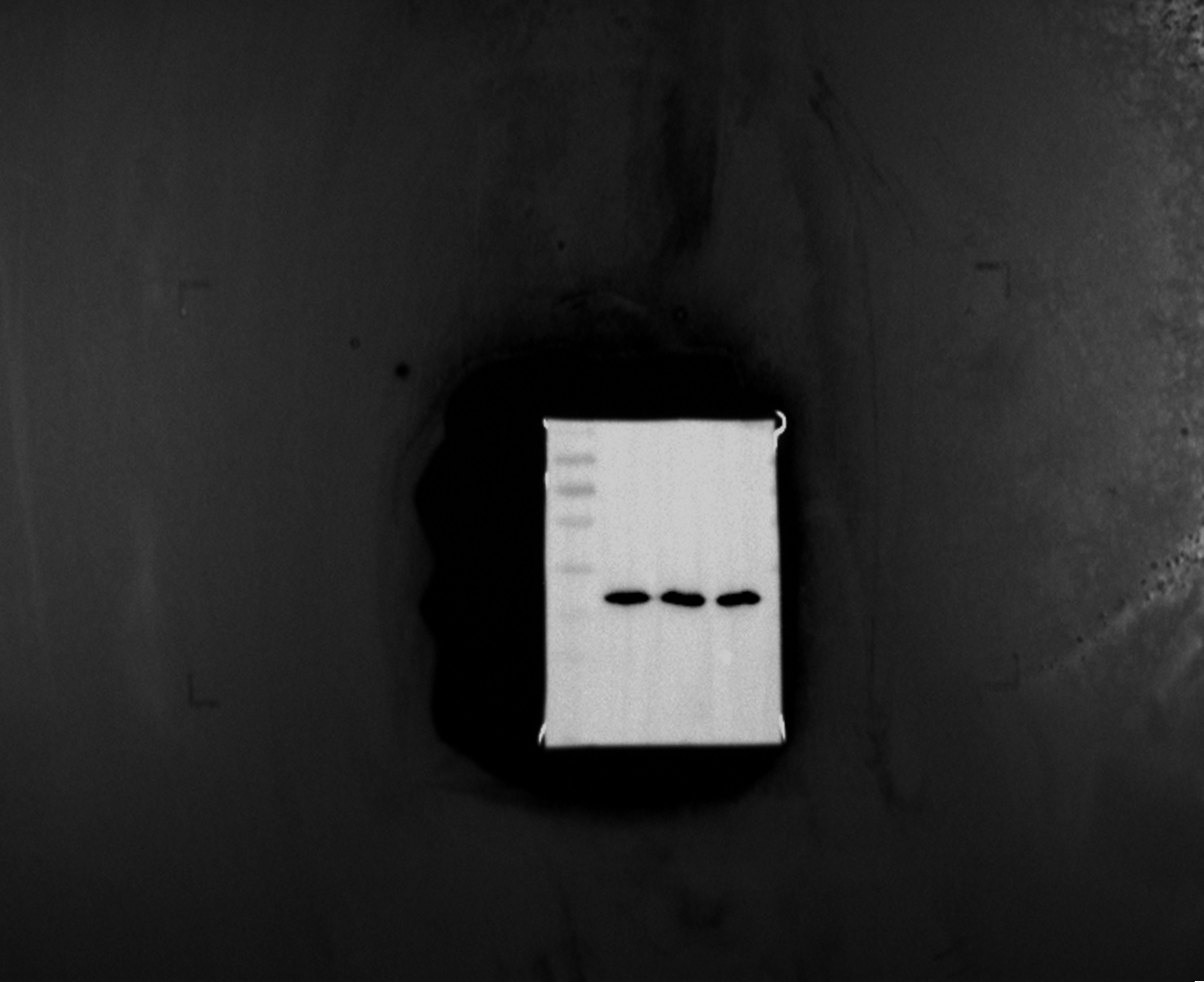


HPAC（GAPDH）

Fig5


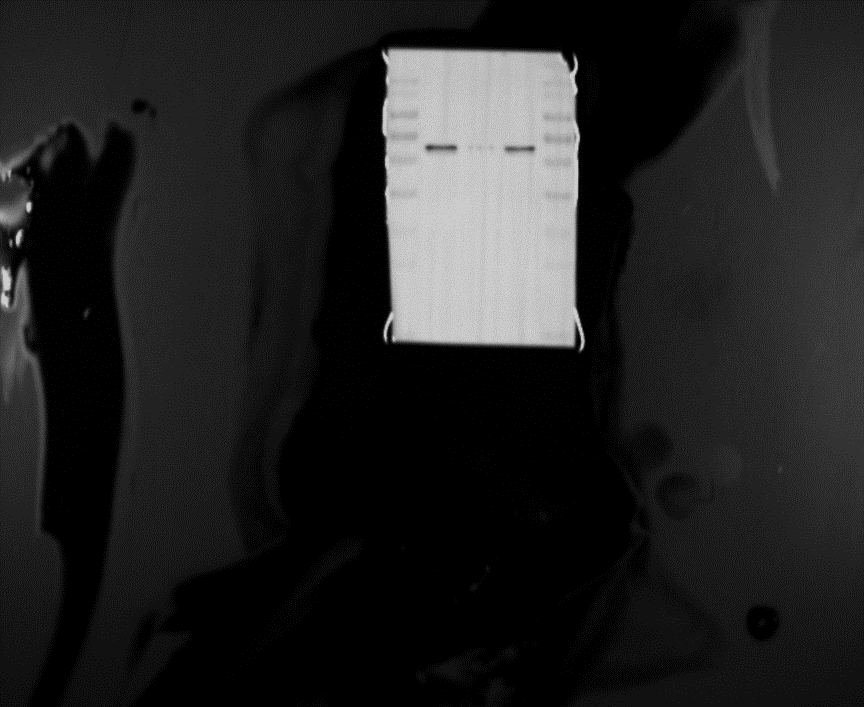


HPAC（FPN1）

）


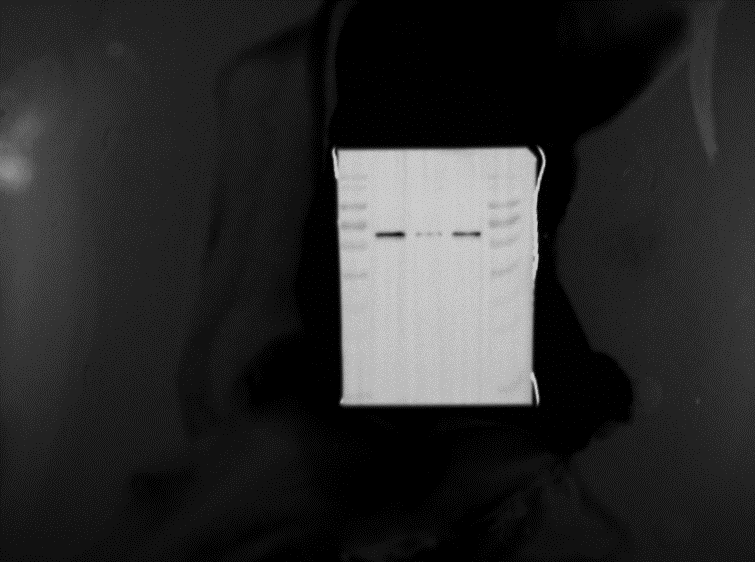


Capan2（FPN1）

）


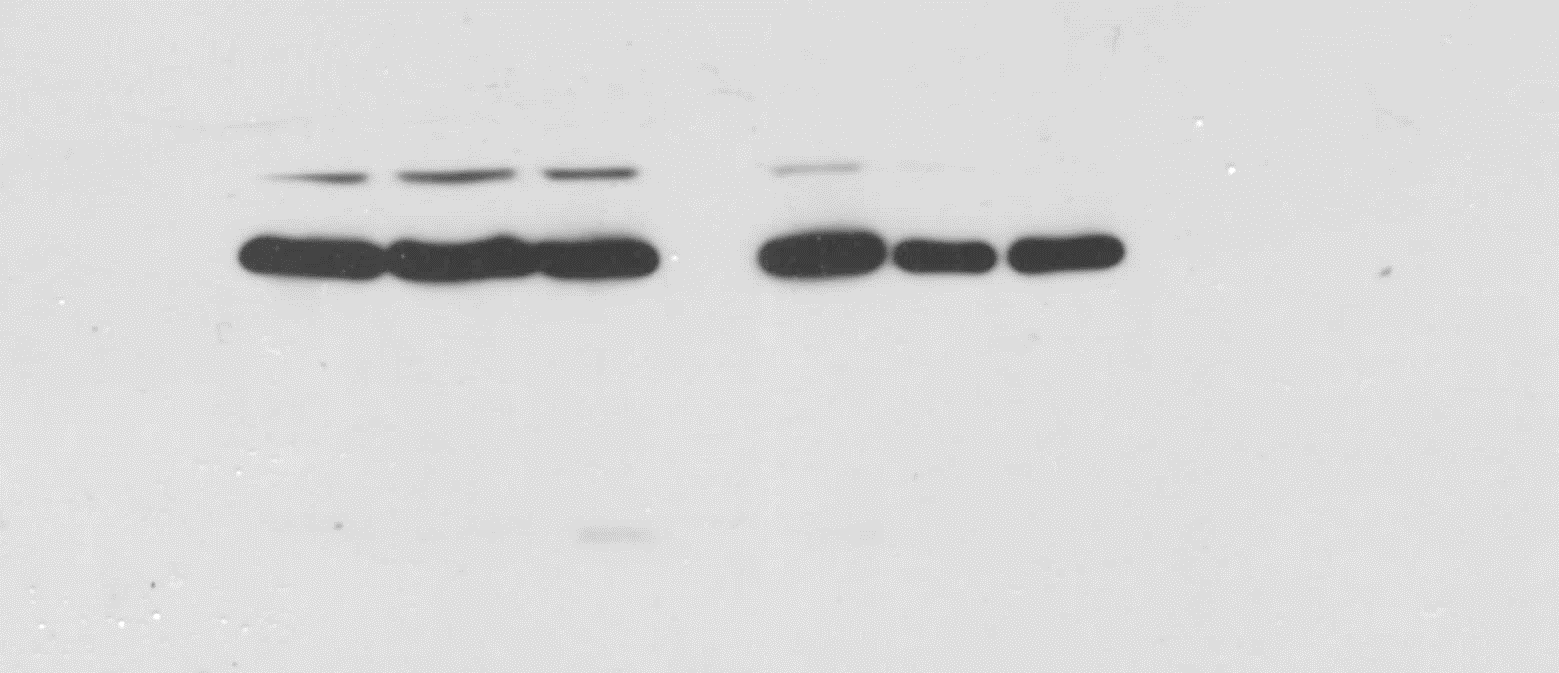


GAPDH

）


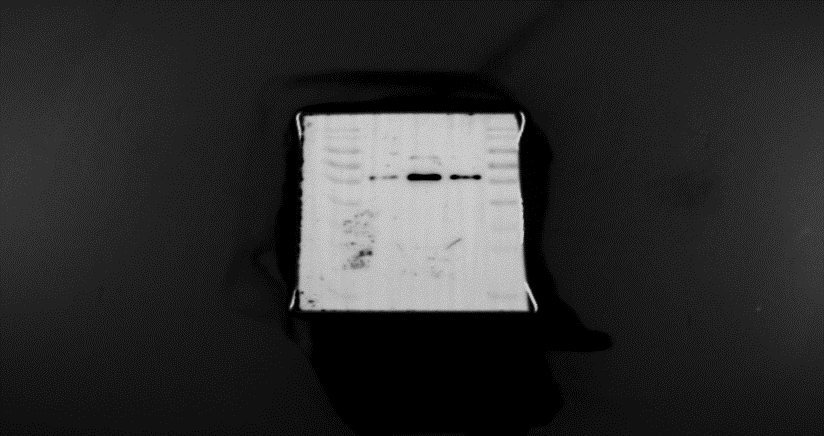


HPAC（p53）

）


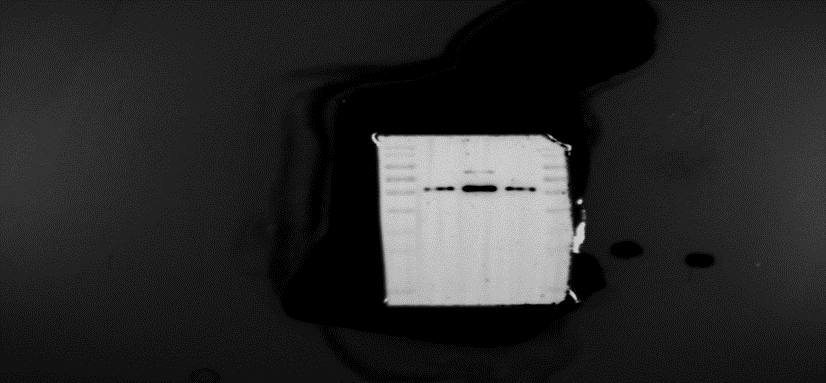


Capan2（p53）

）


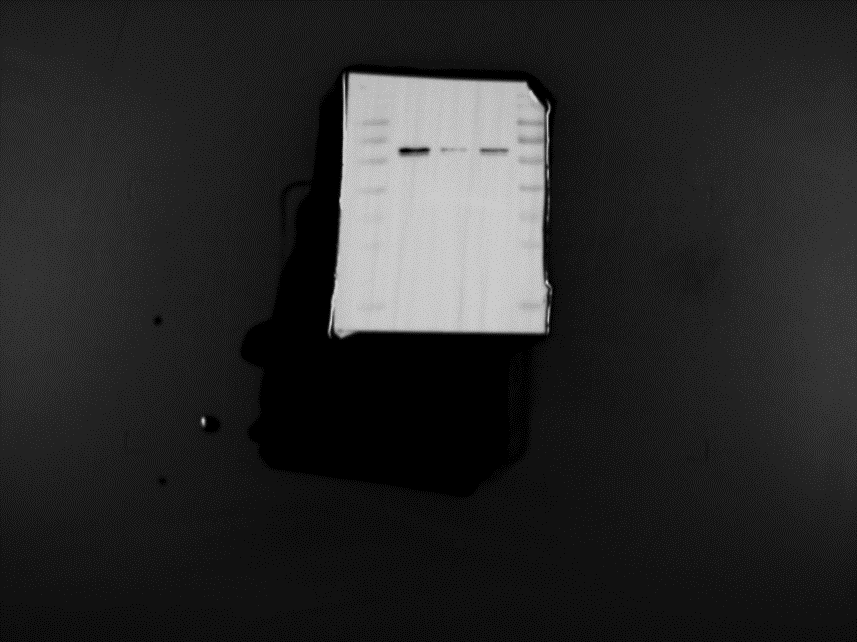


HPAC（FPN1）

）


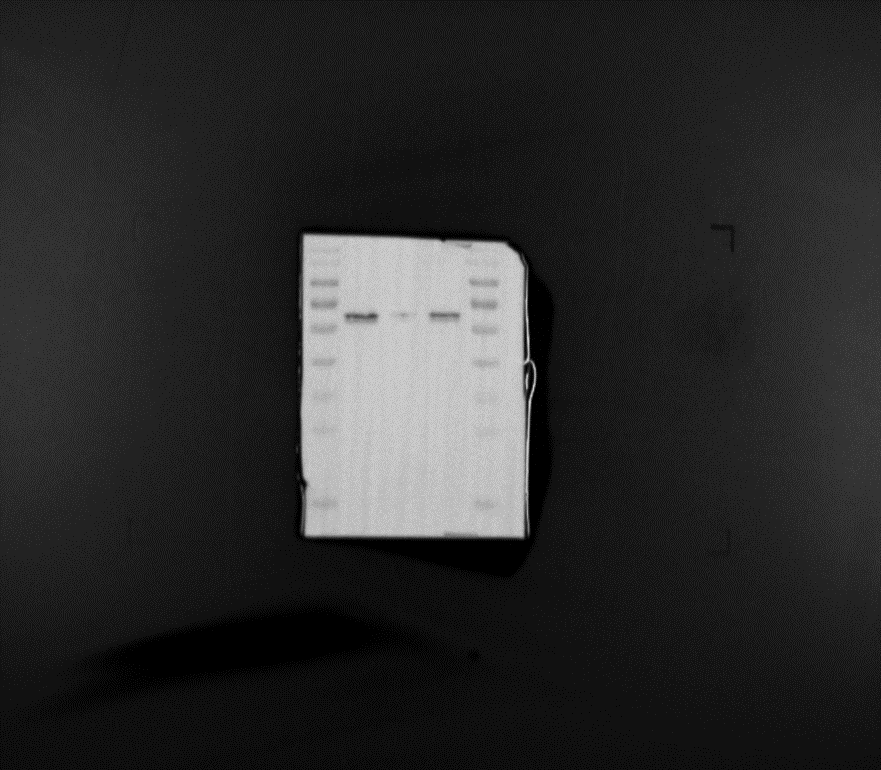


Capan2（FPN1）

）


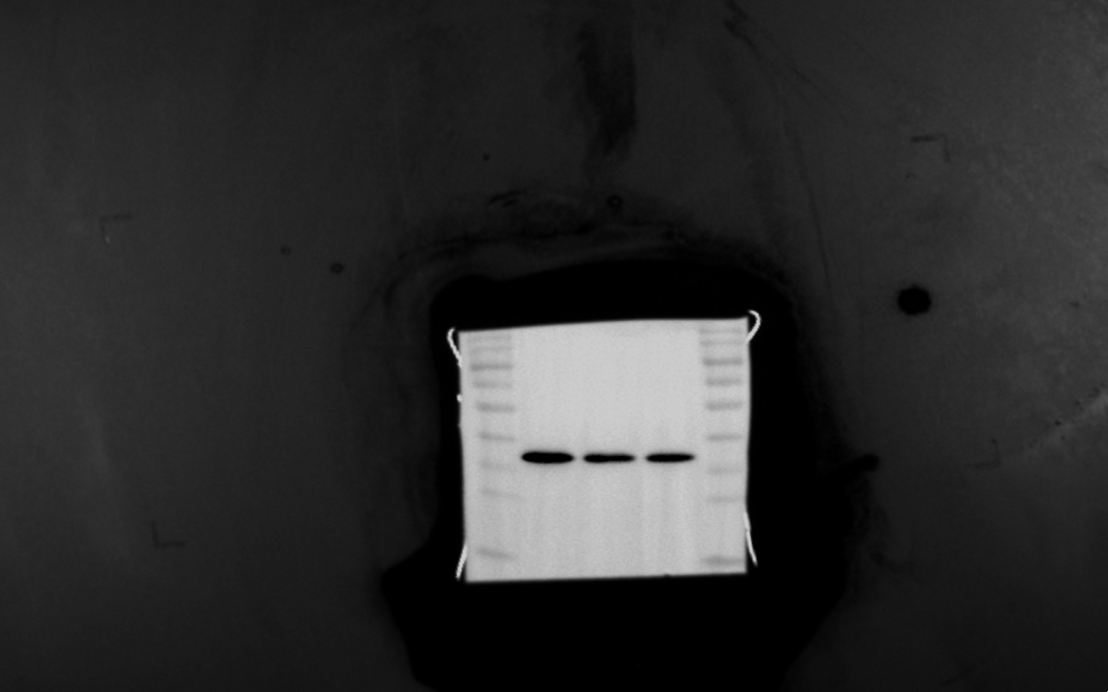


HPAC (GAPDH)

）


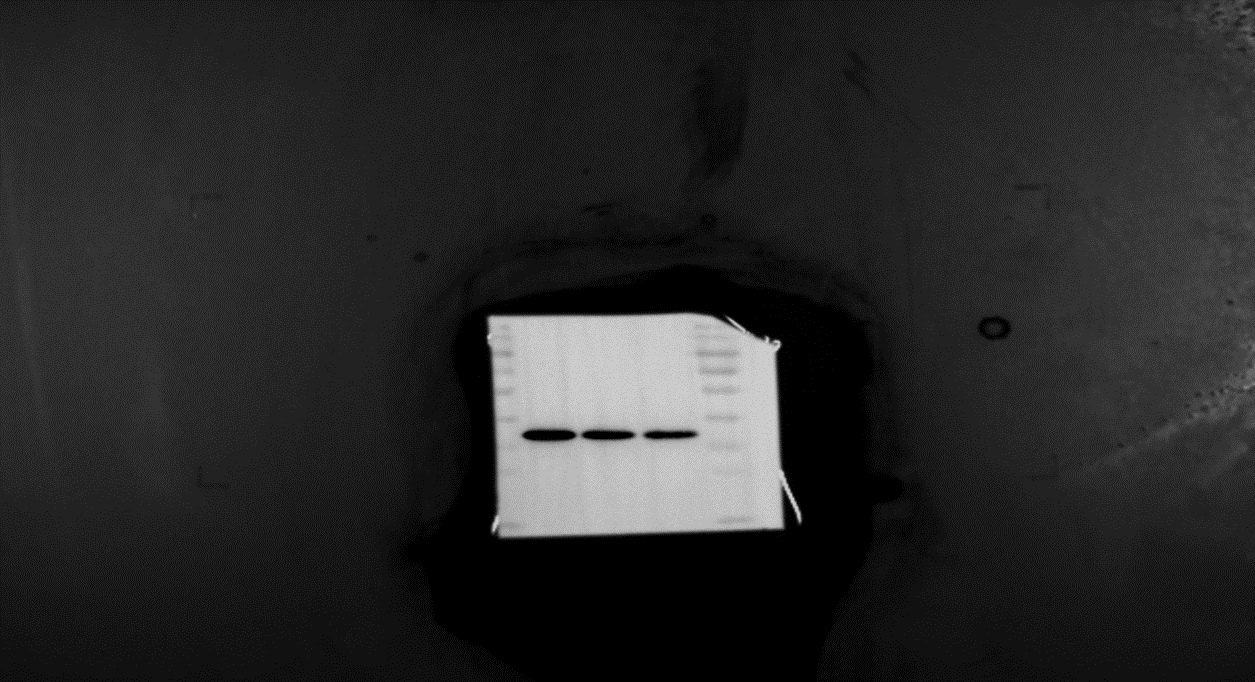


Capan2 (GAPDH)

）

Sup Fig2


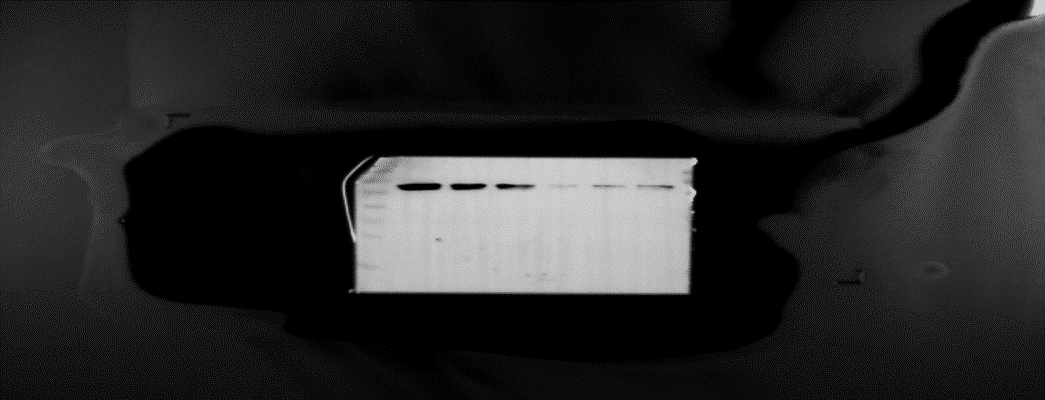


KC&KC Agr2^-/-^ (FPN1)

）


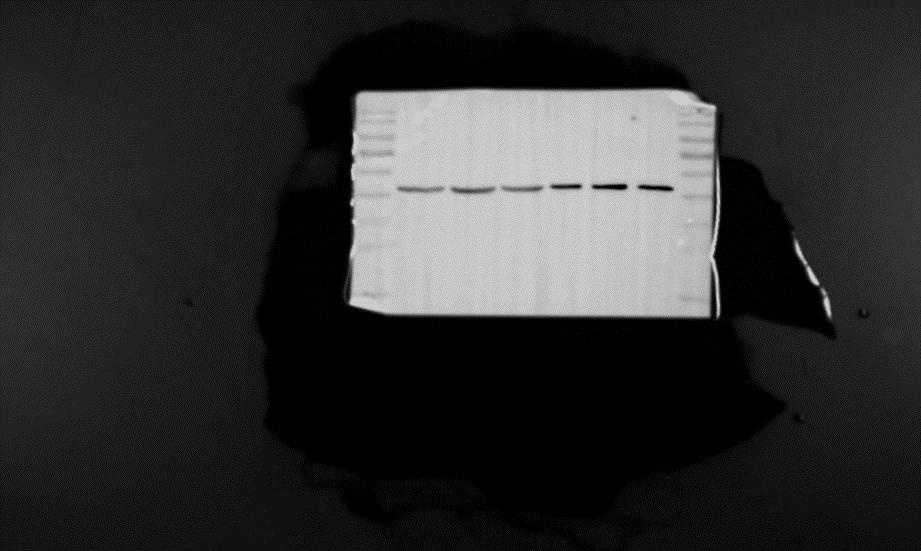


KC&KC Agr2^-/-^ (GPX4)

）


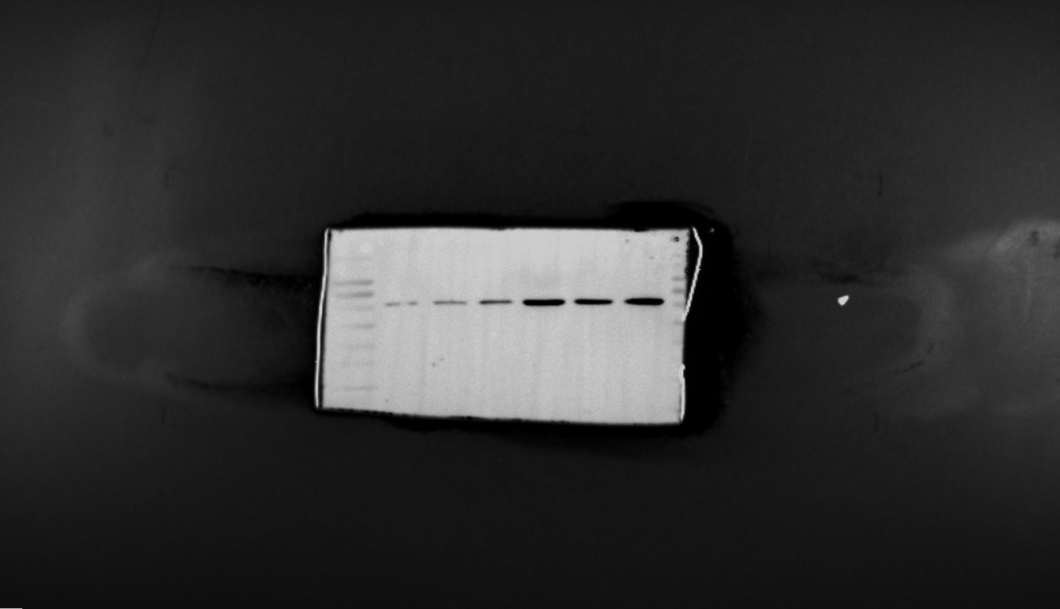


KC&KC Agr2^-/-^ (p53)

）


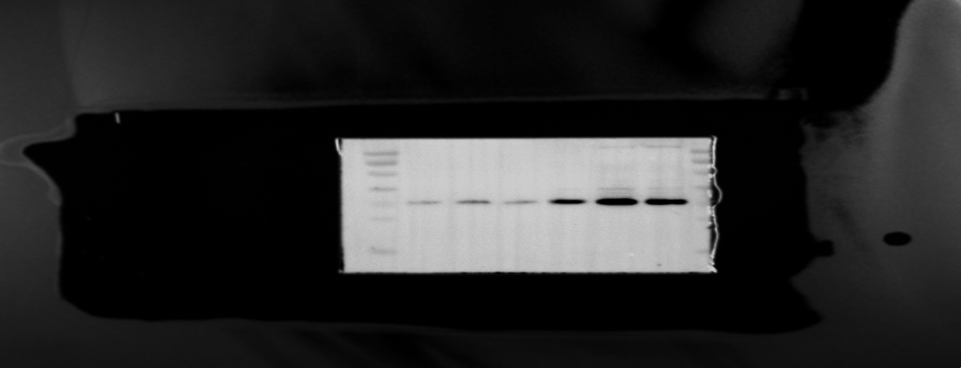


KC&KC Agr2^-/-^ (SLC7A11)

）


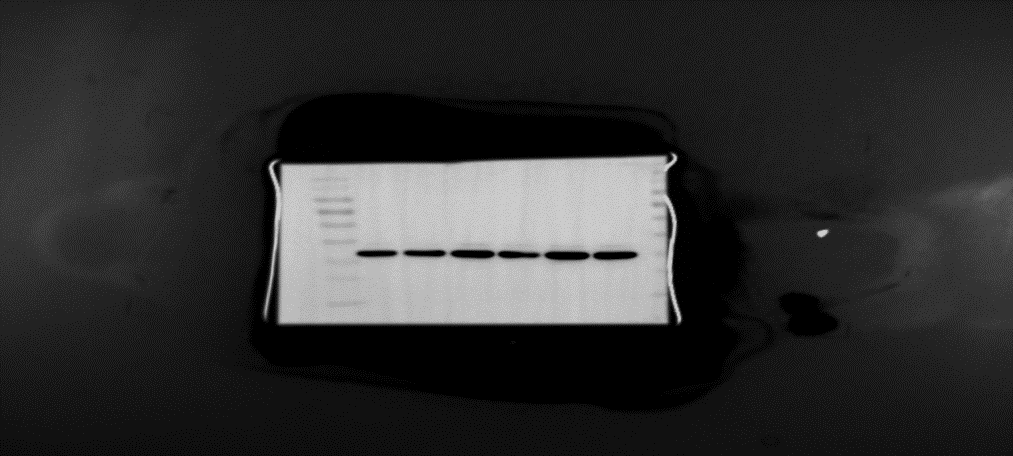


KC&KC Agr2^-/-^ (GAPDH)

）

Fig6


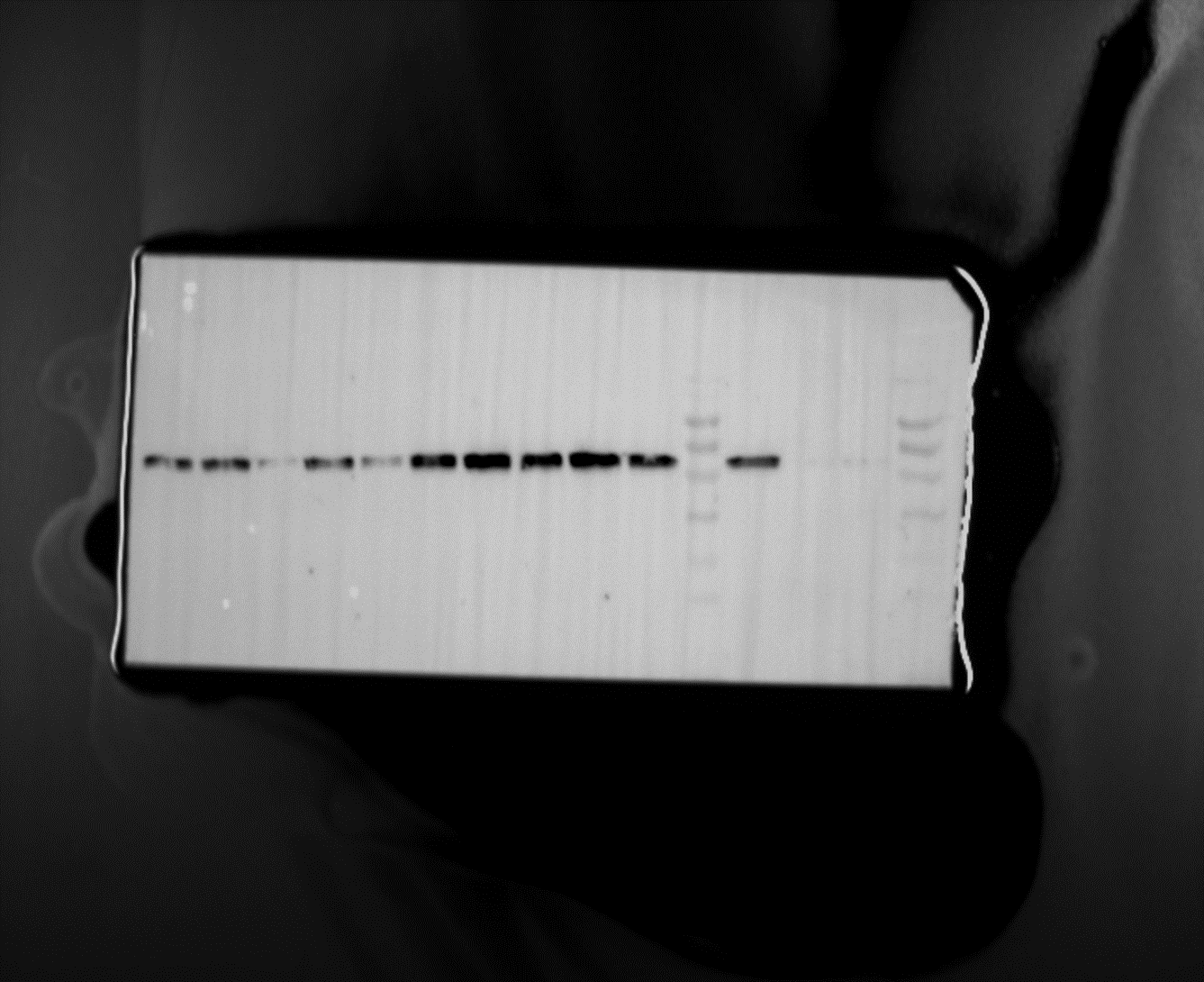


HPAC（FPN1）

）


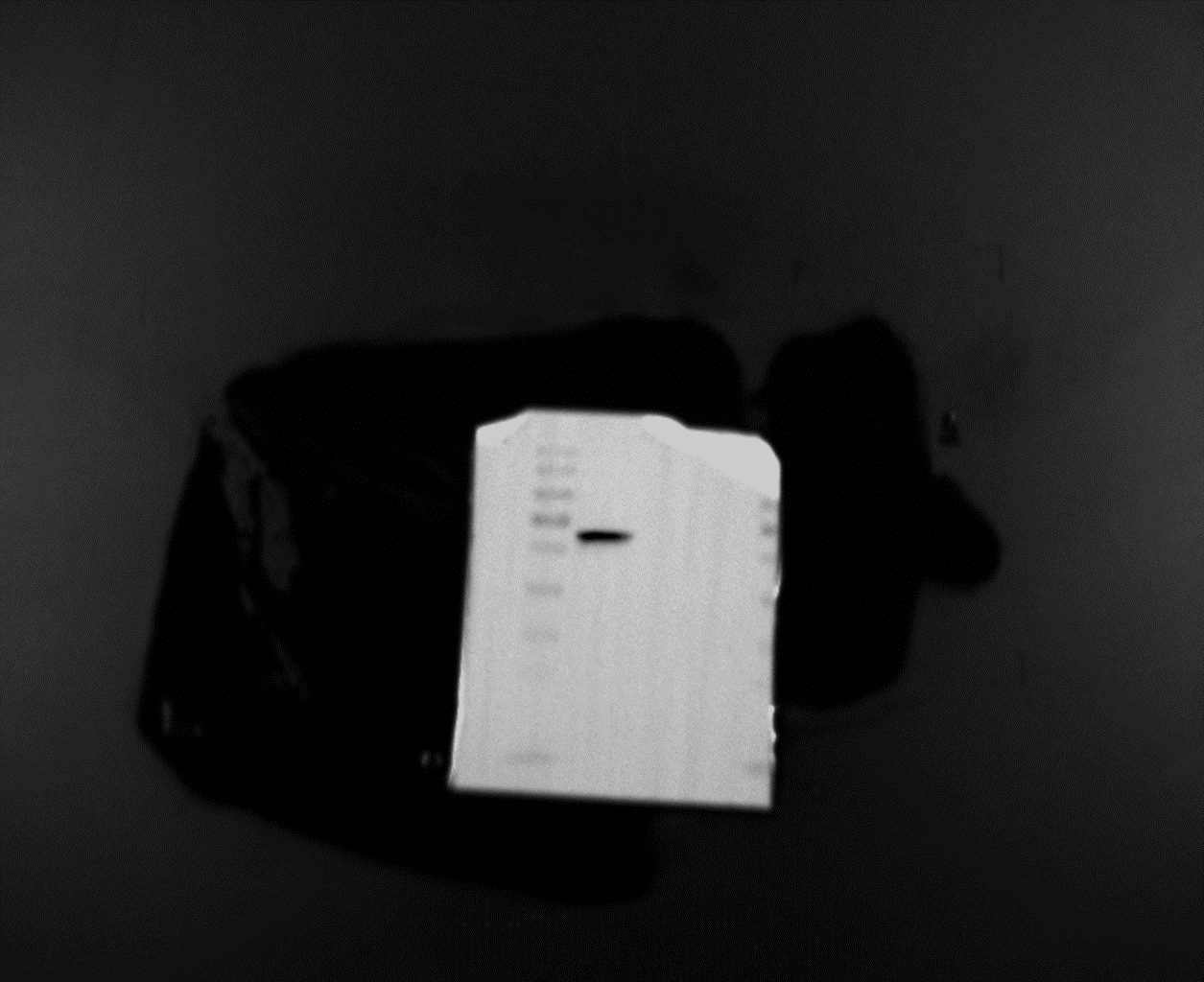


Capan2（FPN1）

）


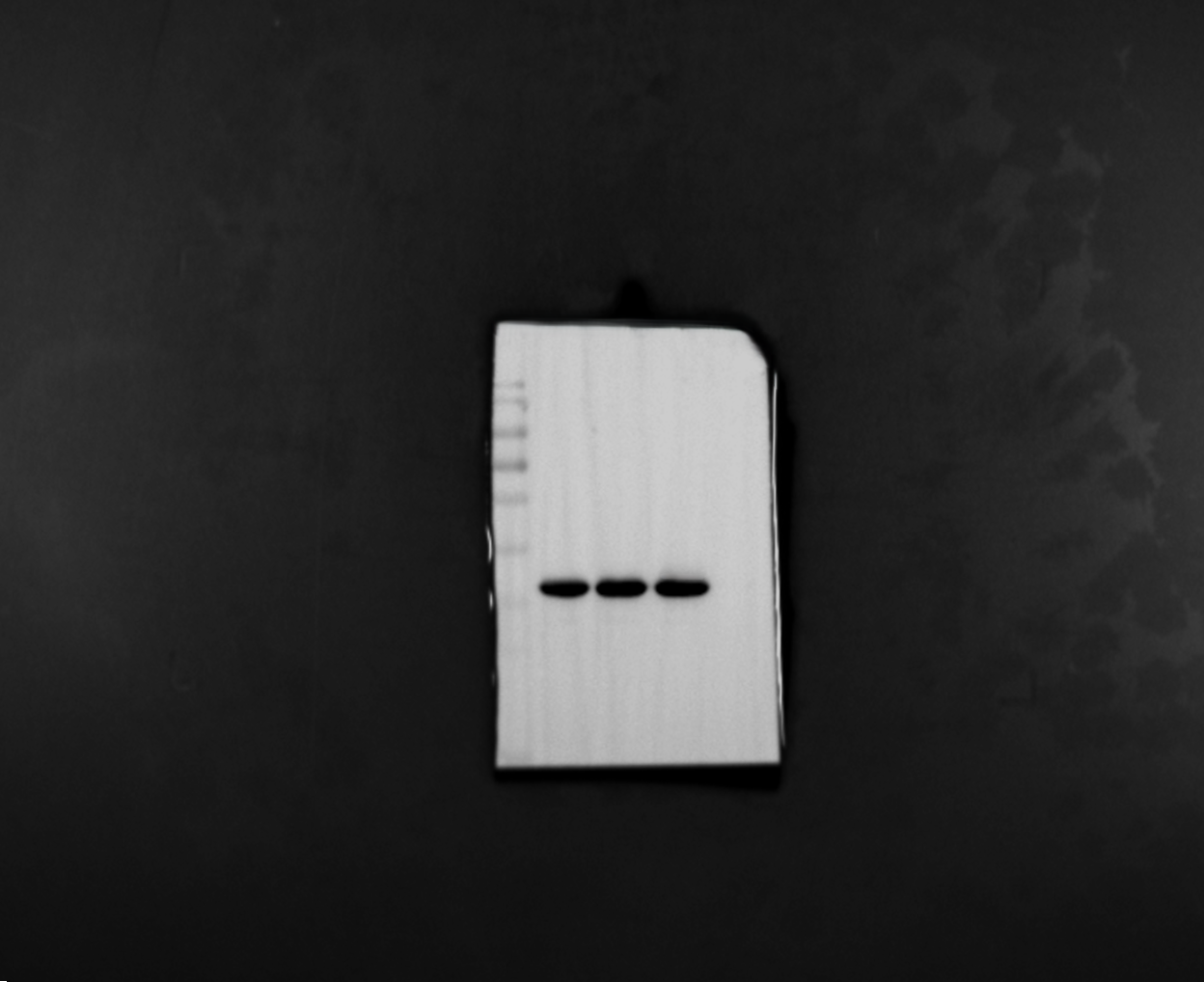


HPAC (GAPDH)

）


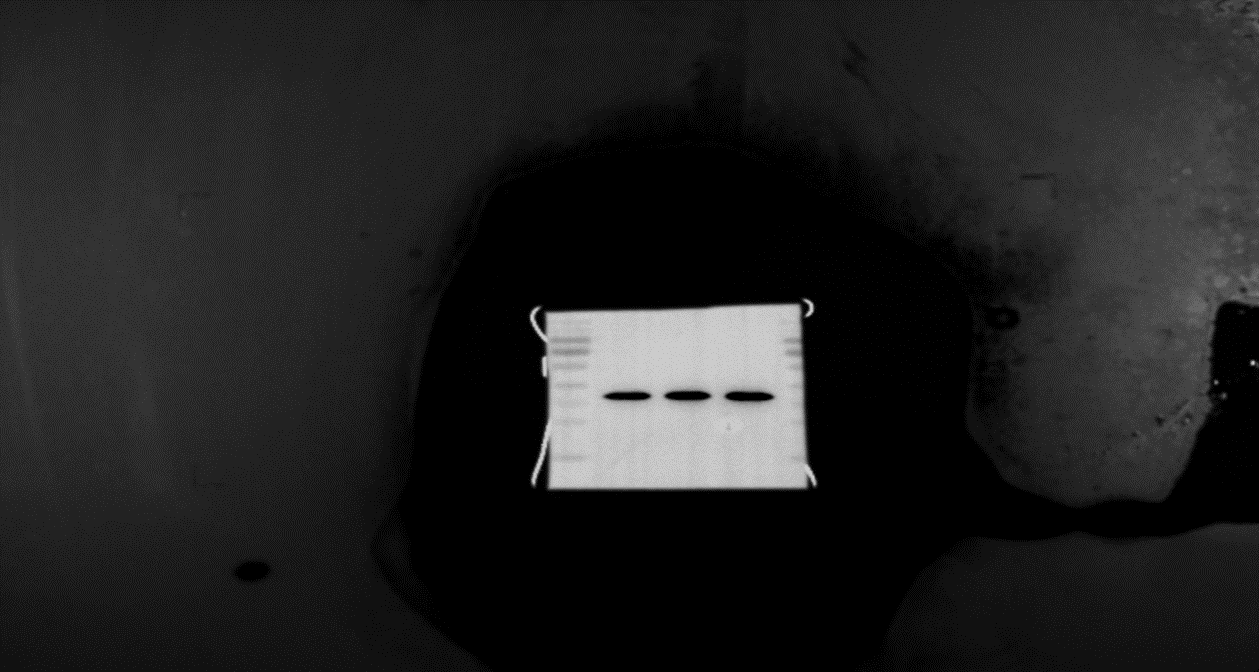


Capan2 (GAPDH)

）

Fig7


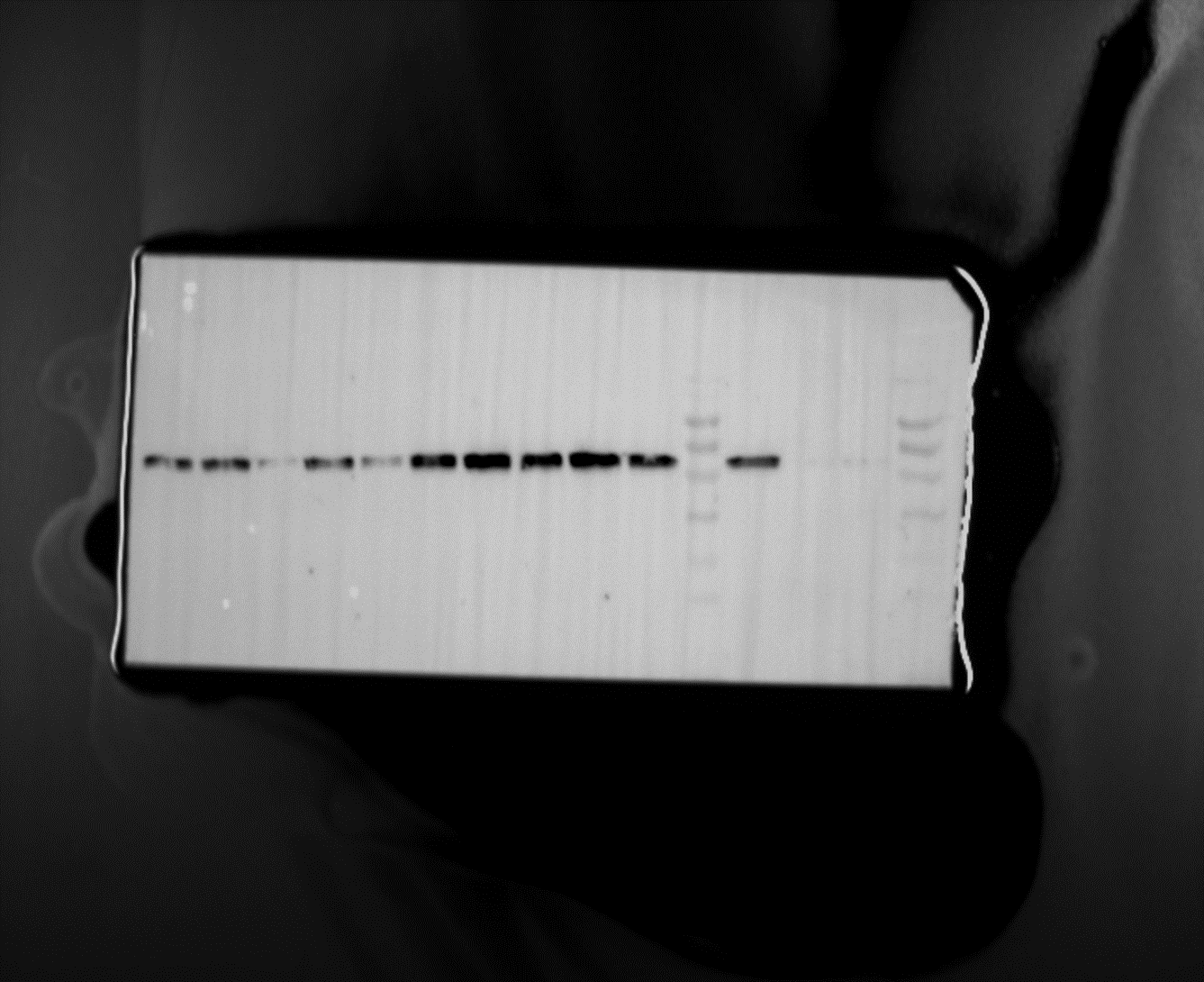


pancreas PDAC(FPN1)

）


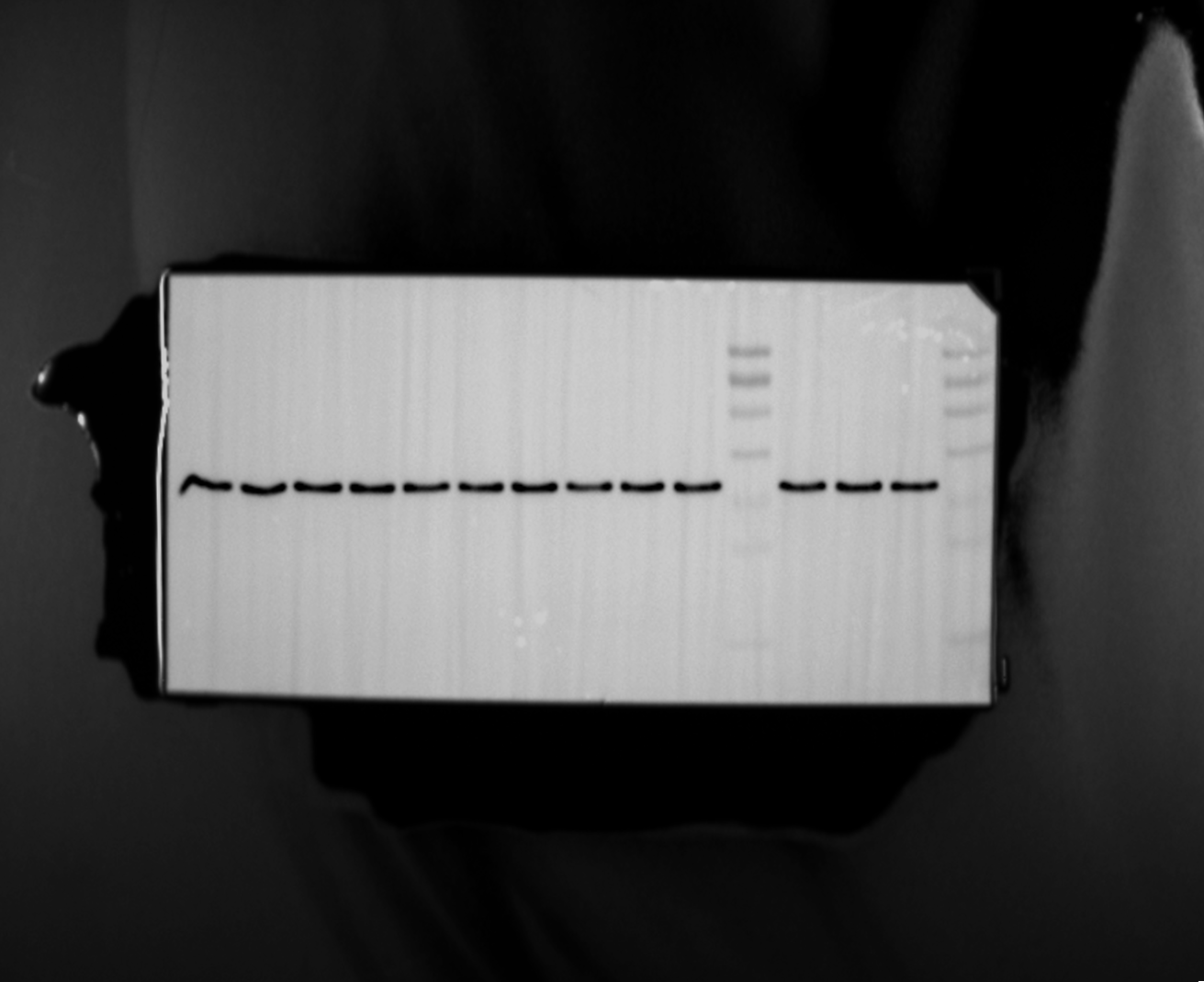


pancreas PDAC(GAPDH)

Fig8


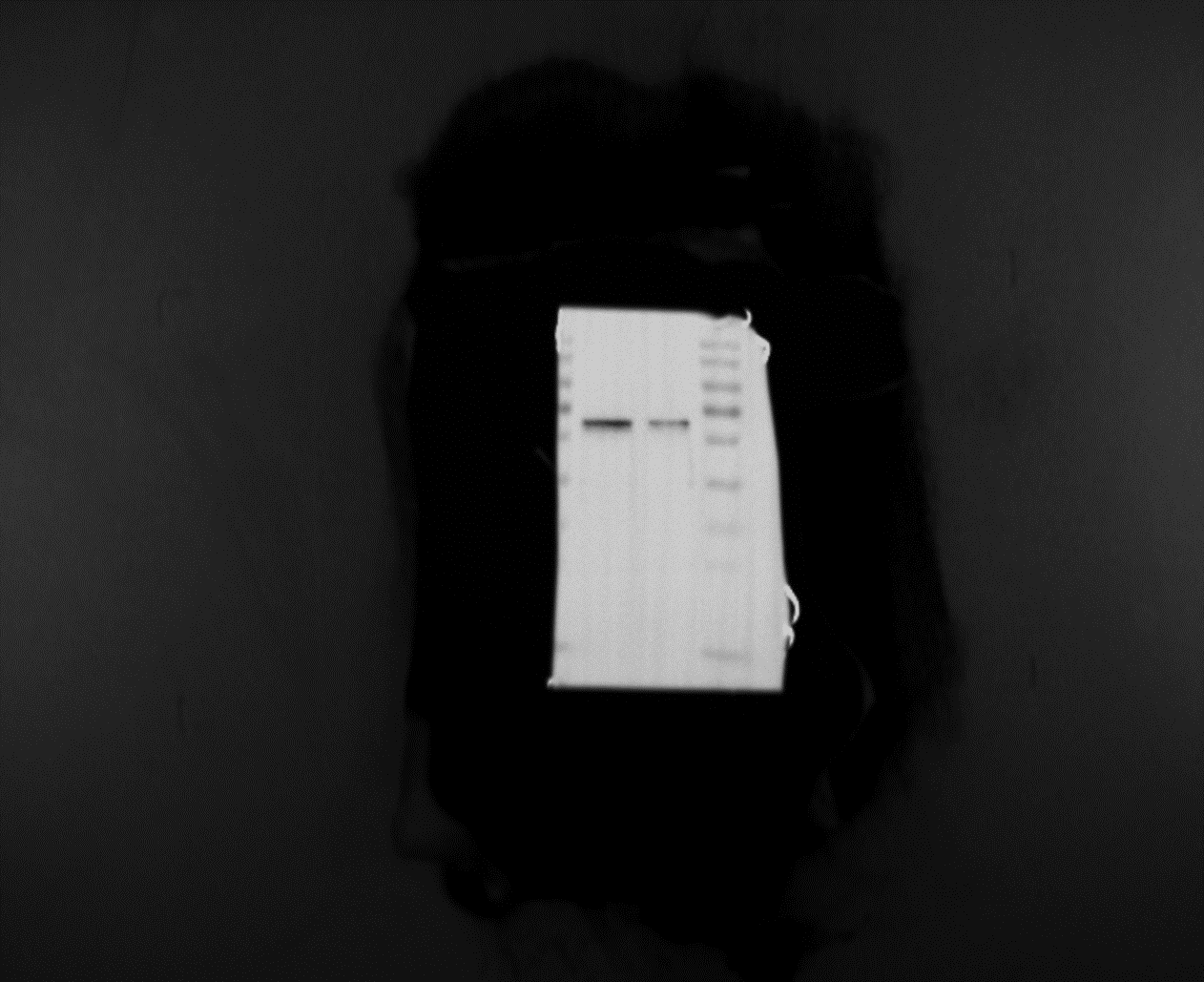


HPAC（FPN1）

）


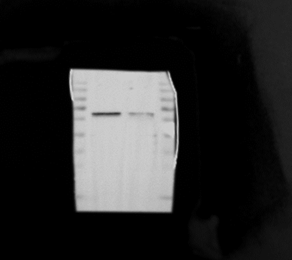


Capan2（FPN1）

）


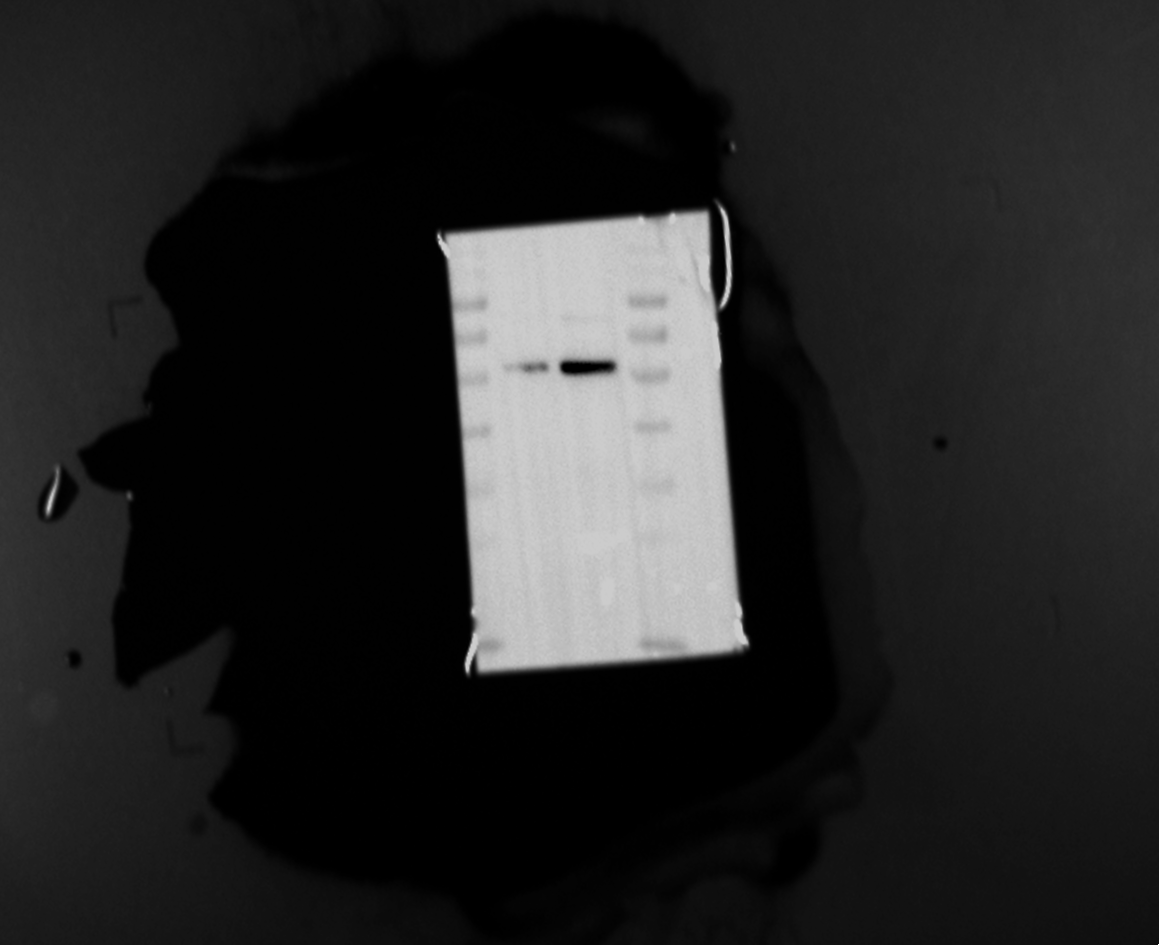


HPAC（p53）

）


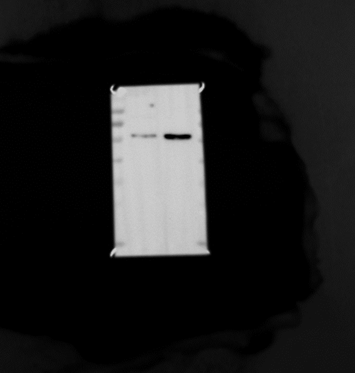


Capan2（p53）

）


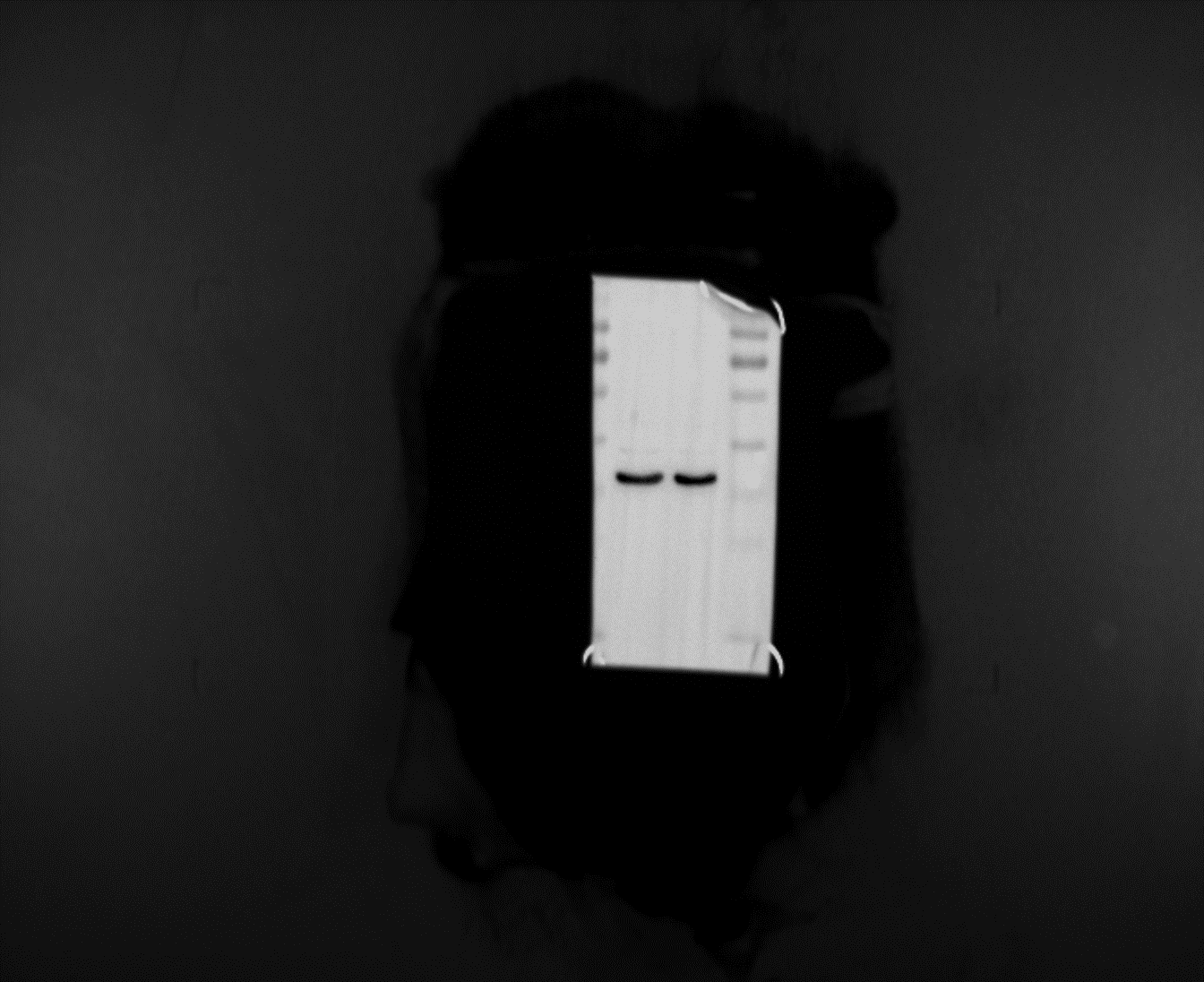


HPAC（GAPDH）

）


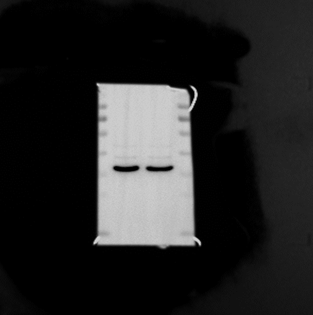


Capan2（GAPDH）

）
