# Supplementary figures and images for: AGR2 suppresses ferroptosis via the p53/FPN1 regulatory axis and drives therapeutic vulnerabilities in pancreatic cancer
Source: Cell Death Dis. 2025 Dec 1;16(1):877. doi: 10.1038/s41419-025-08263-y (PMC12669619; doi:10.1038/s41419-025-08263-y)

Supplementary Figure 1

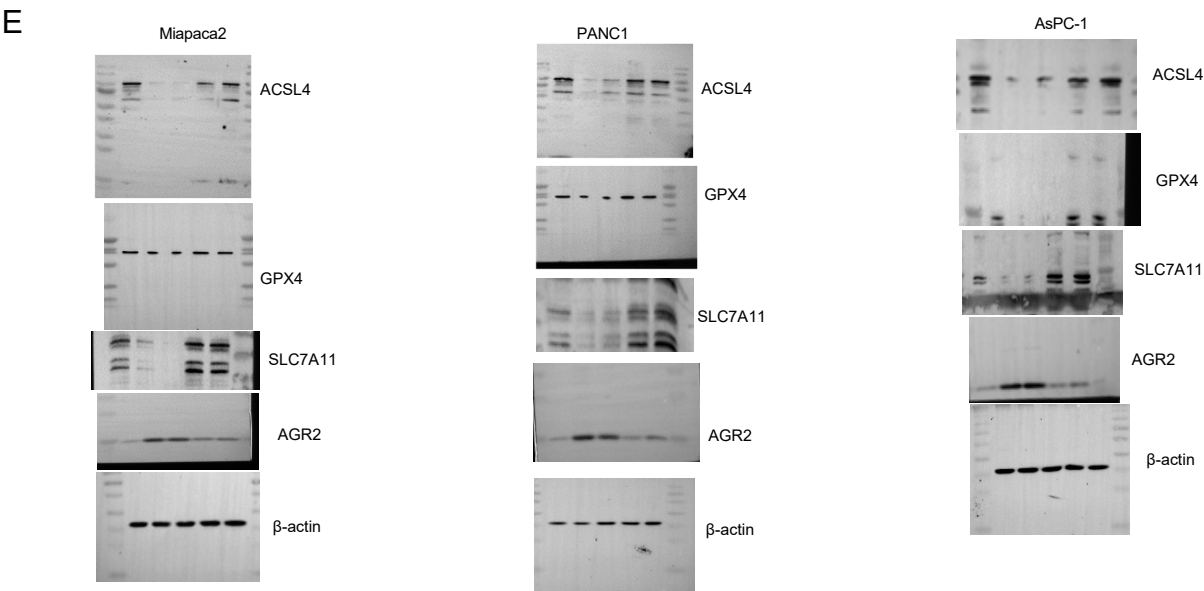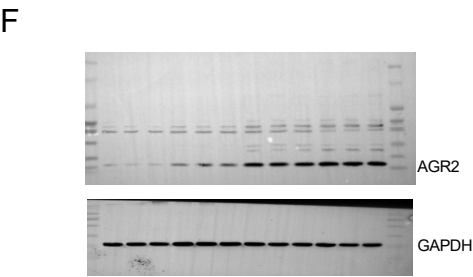

Supplementary Figure 2

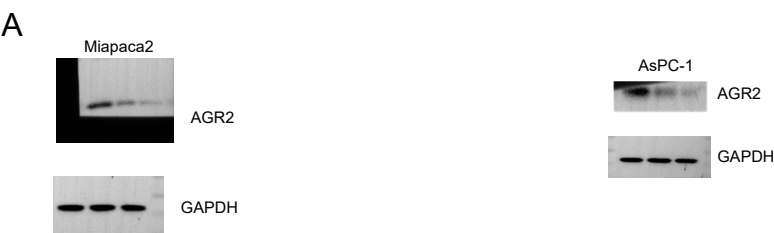

Supplement: Supplementary file 5 — WB revised [file 41419_2025_8263_MOESM5_ESM.pdf]

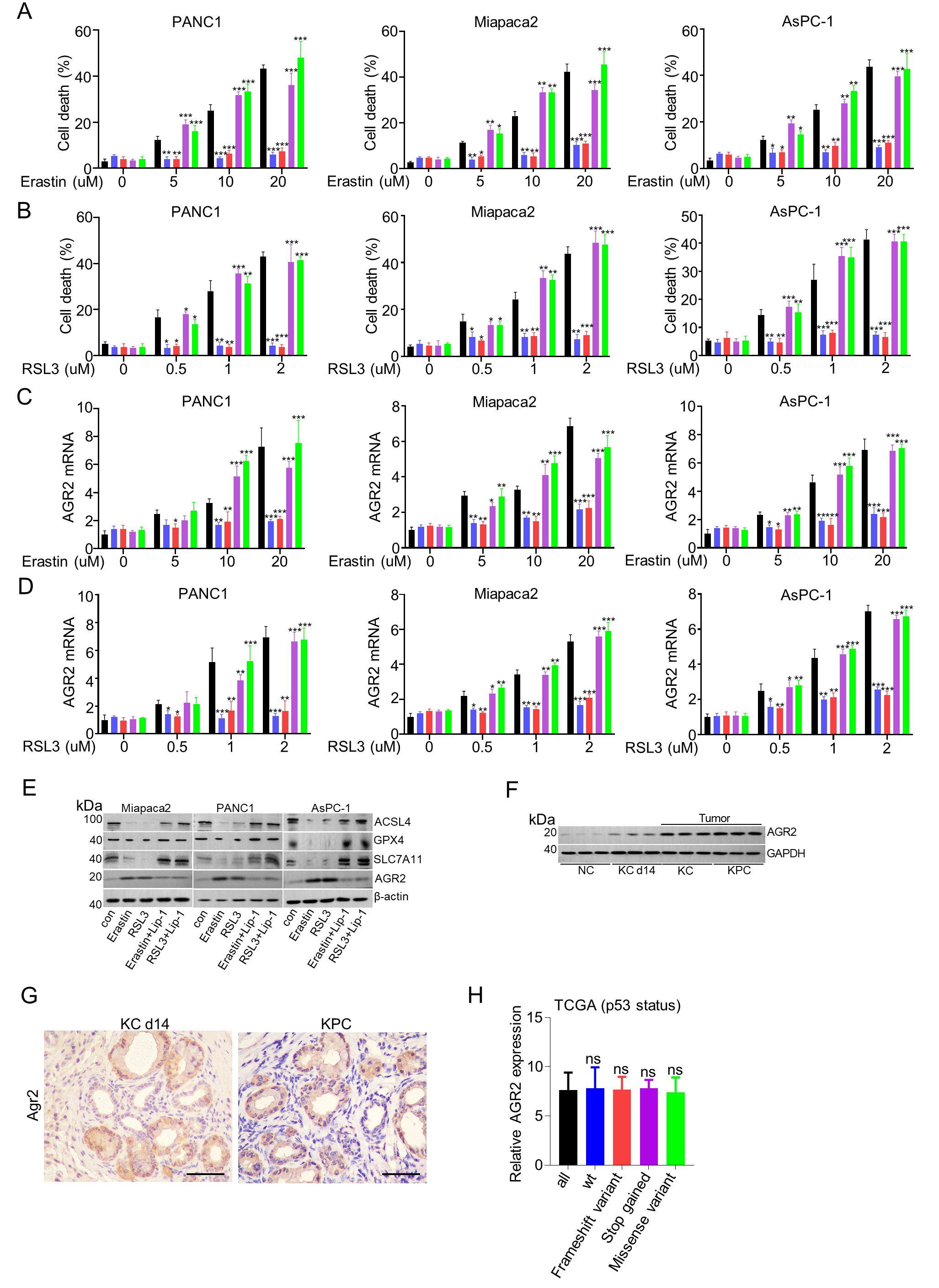

Supplement: Supplementary file 6 — Supplementary figure 1 [file 41419_2025_8263_MOESM6_ESM.png]

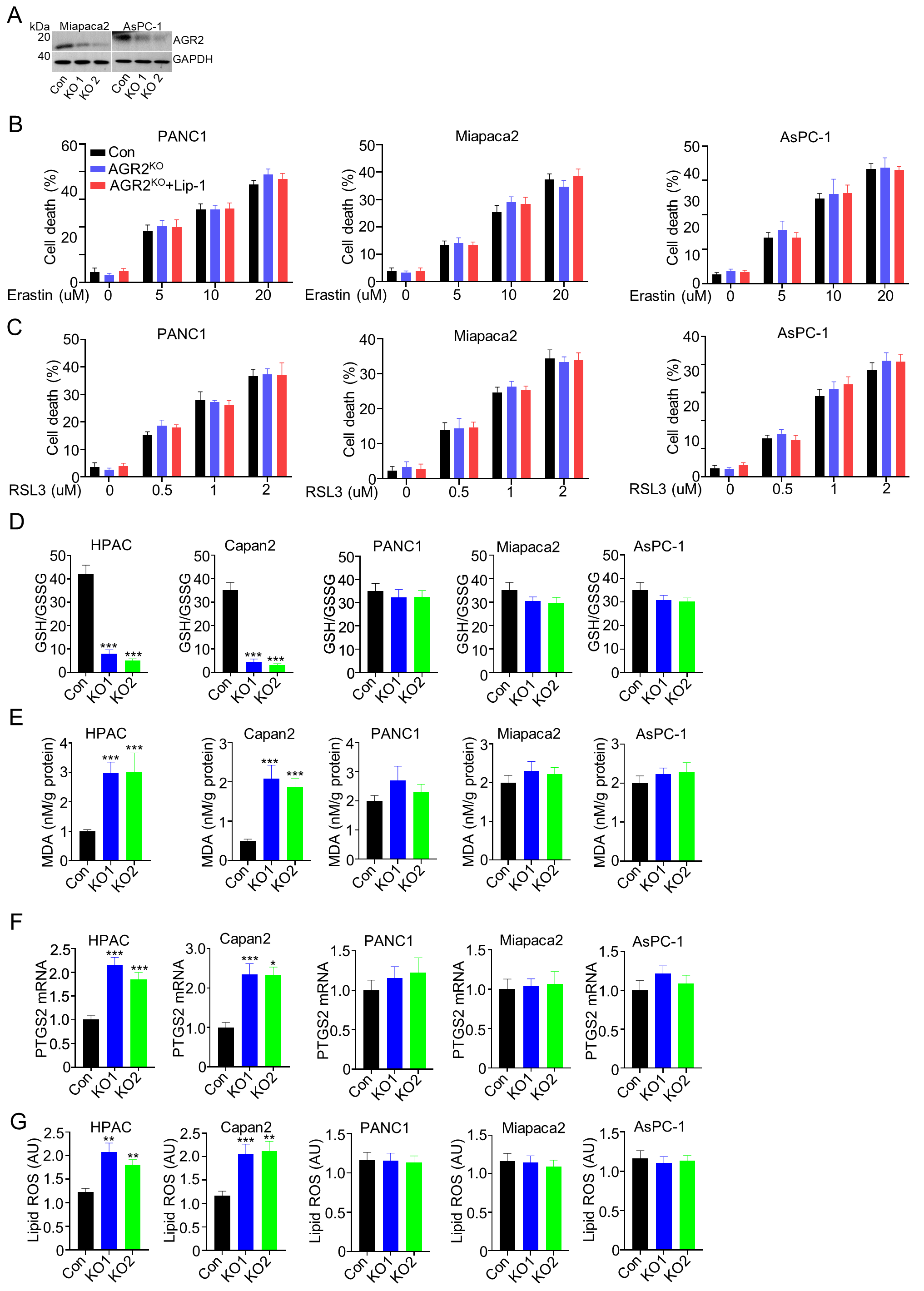

Supplement: Supplementary file 7 — Supplementary figure 2 [file 41419_2025_8263_MOESM7_ESM.png]

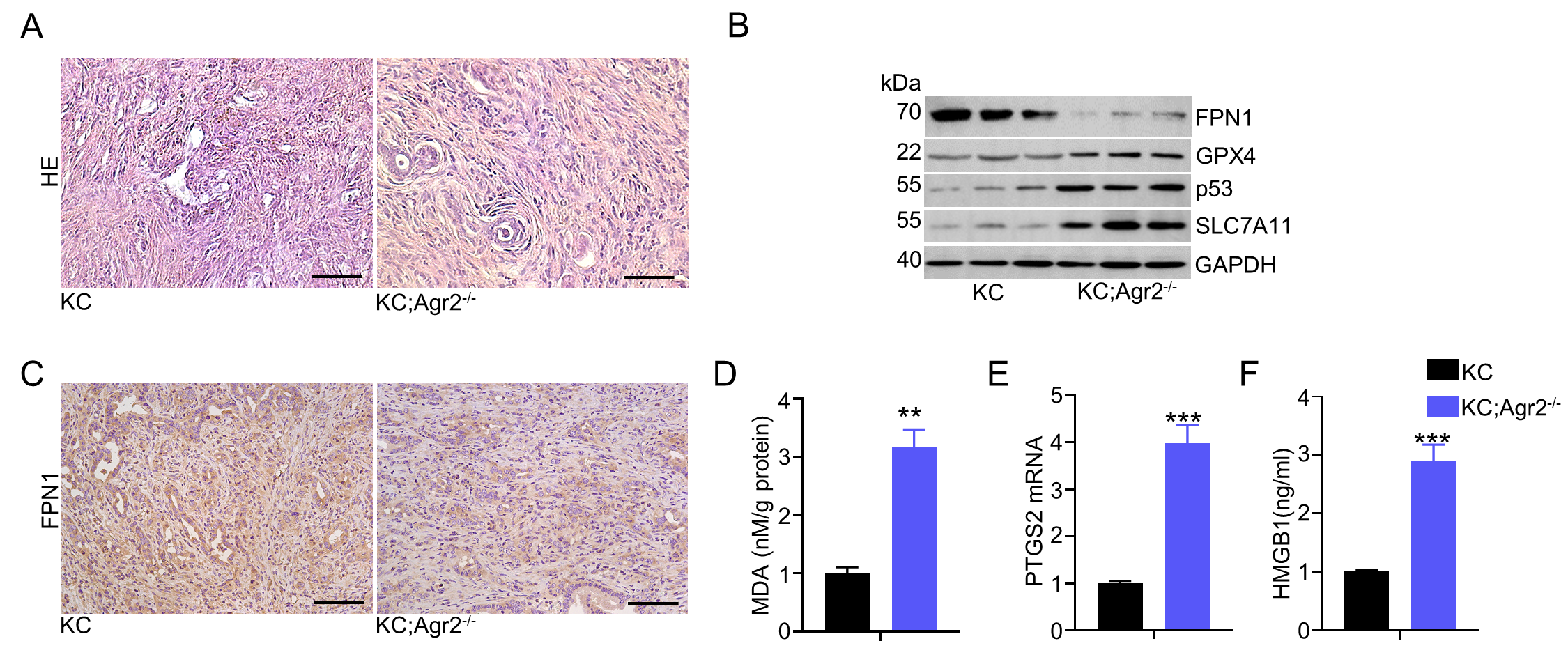

Supplement: Supplementary file 8 — Supplementary figure 3 [file 41419_2025_8263_MOESM8_ESM.png]

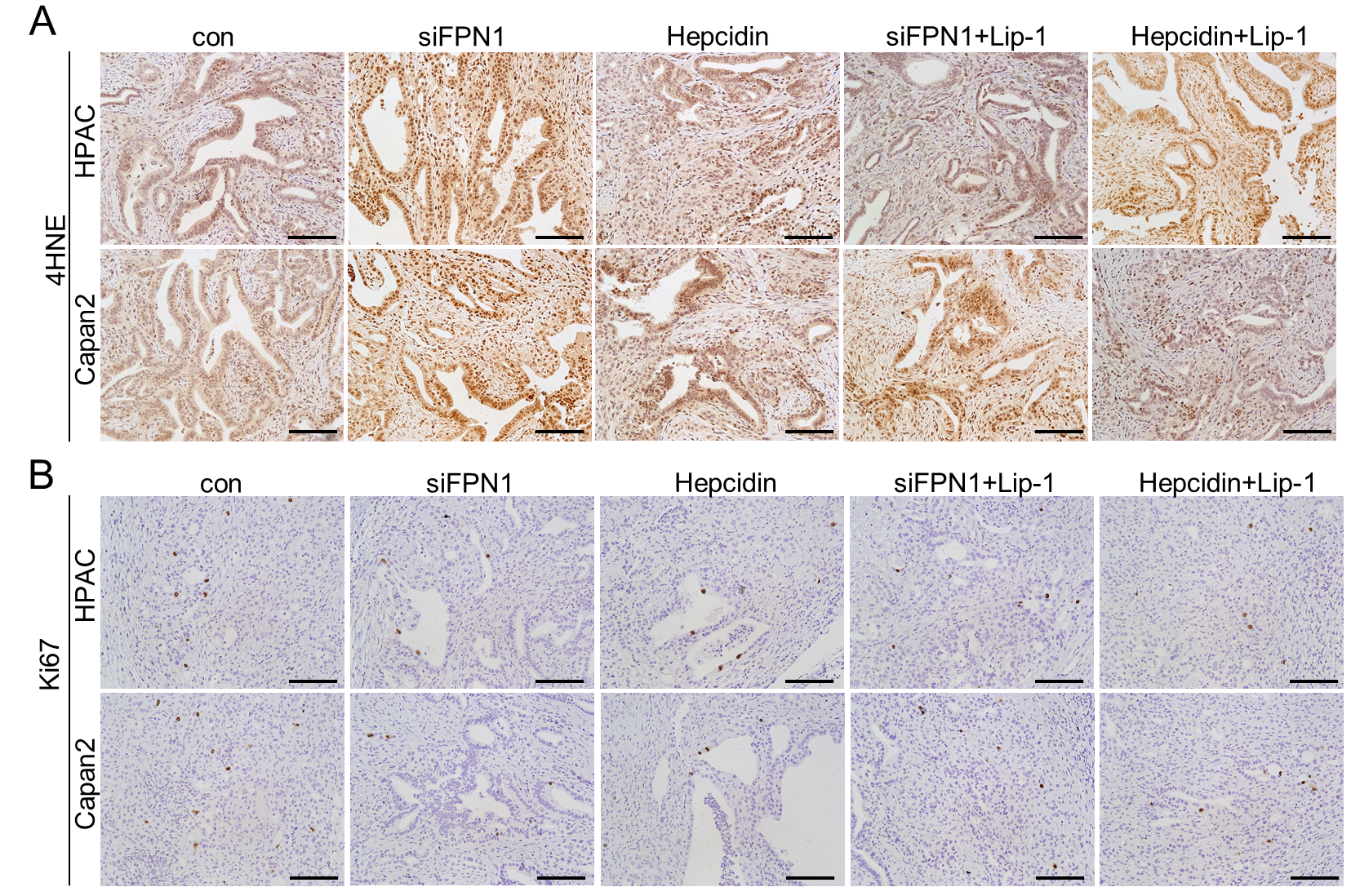

Supplement: Supplementary file 9 — Supplementary figure 4 [file 41419_2025_8263_MOESM9_ESM.png]

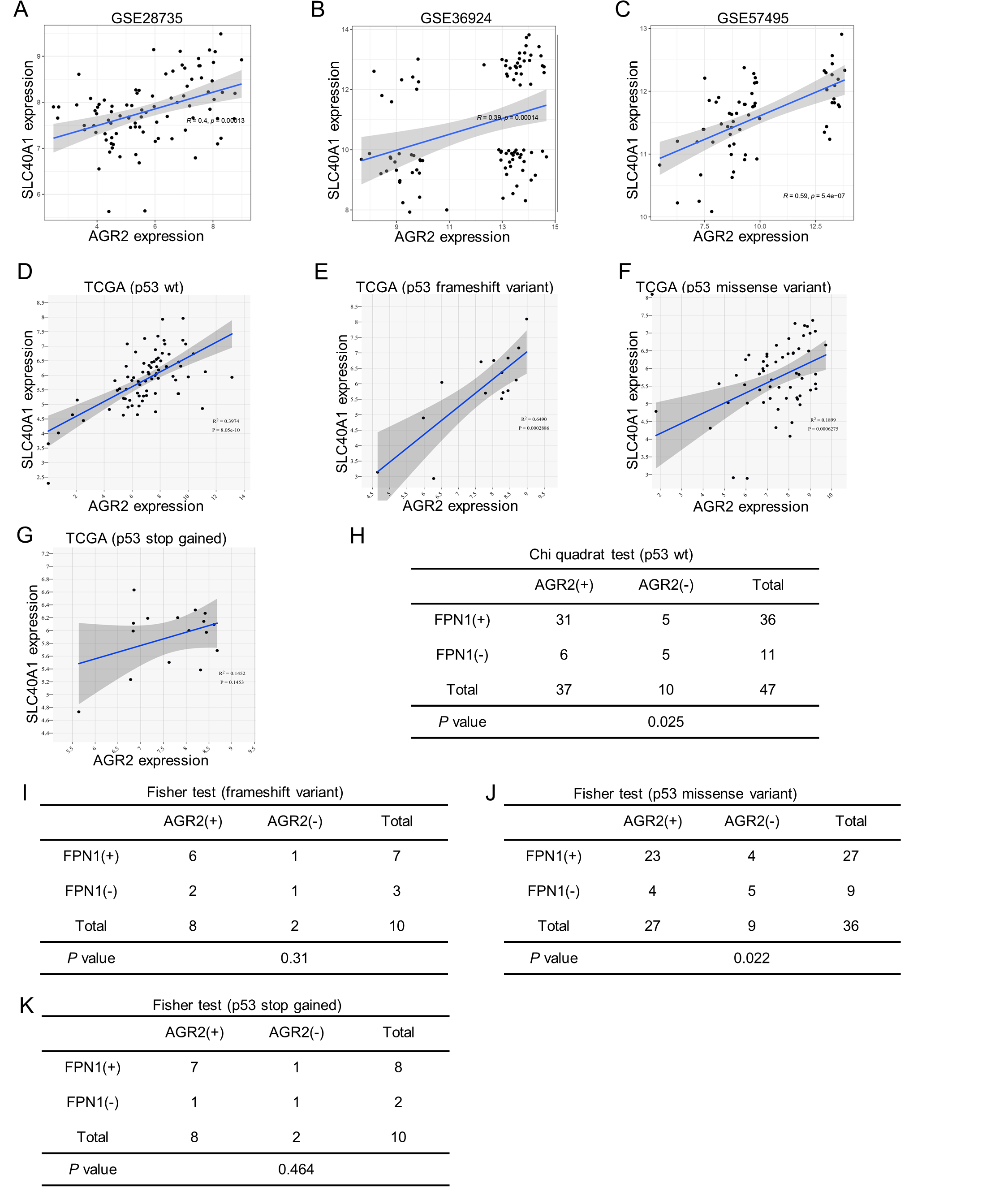

Supplement: Supplementary file 10 — Supplementary figure 5 [file 41419_2025_8263_MOESM10_ESM.png]

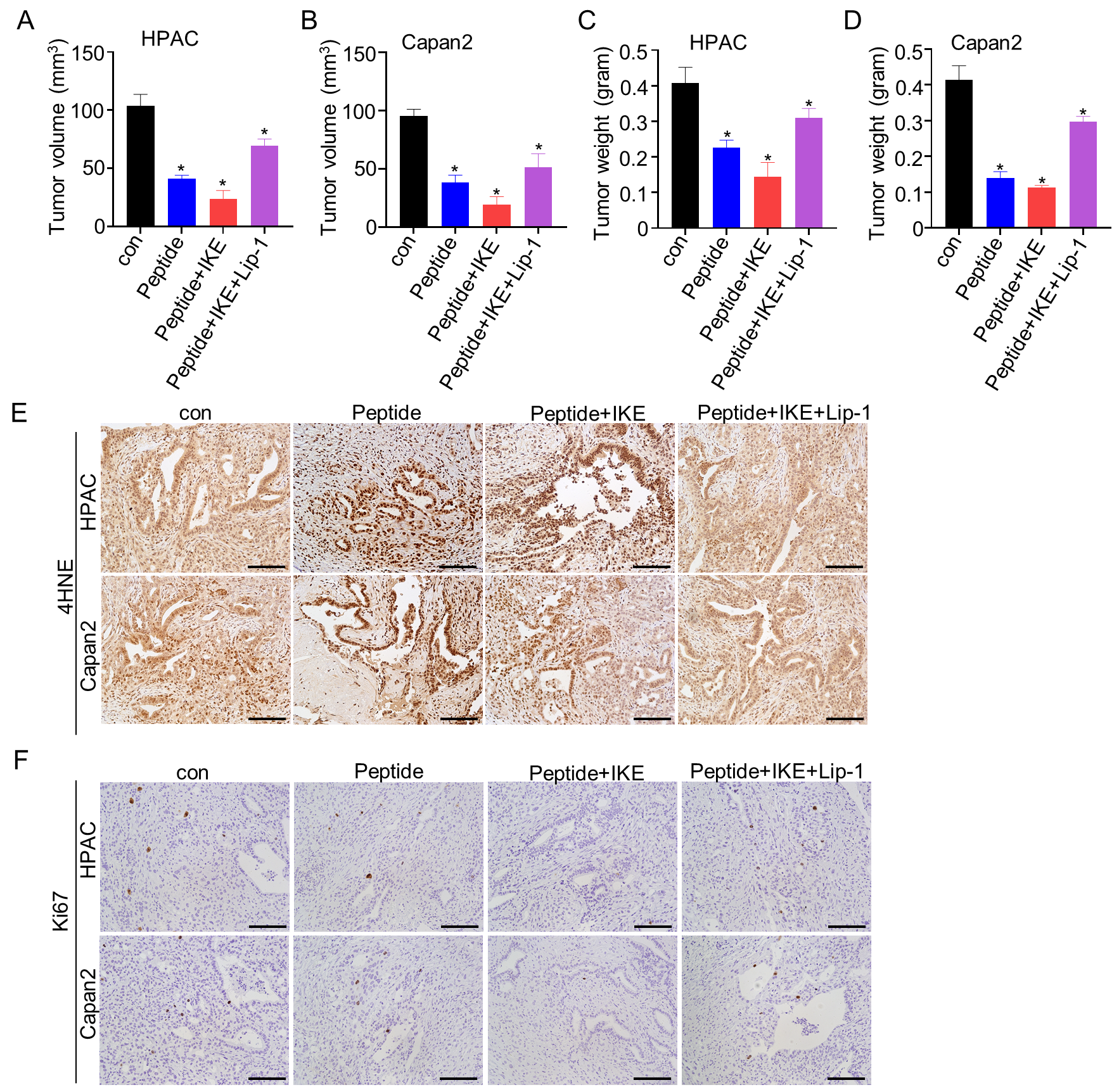

Supplement: Supplementary file 11 — Supplementary figure 6 [file 41419_2025_8263_MOESM11_ESM.png]
